# Along the Axis between Type 1 and Type 2 Immunity; Principles Conserved in Evolution from Fish to Mammals

**DOI:** 10.3390/biology4040814

**Published:** 2015-11-17

**Authors:** Takuya Yamaguchi, Fumio Takizawa, Uwe Fischer, Johannes M. Dijkstra

**Affiliations:** 1 Laboratory of Fish Immunology, Institute of Infectology, Friedrich-Loeffler-Institut, Südufer 10, Greifswald-Insel Riems 17493, Germany; E-Mails: Takuya.Yamaguchi@fli.bund.de (T.Y.); Uwe.Fischer@fli.bund.de (U.F.); 2 Department of Pathobiology, School of Veterinary Medicine, University of Pennsylvania, Philadelphia, PA 19104, USA; E-Mail: fam64tak@hotmail.com; 3 Institute for Comprehensive Medical Science, Fujita Health University, Dengakugakubo 1-98, Toyoake, Aichi 470-1192, Japan

**Keywords:** immunology, evolution, fish, T_H_1, T_H_17, T_reg_, T_H_2, i1-i2 axis, cytokines, IL-5

## Abstract

A phenomenon already discovered more than 25 years ago is the possibility of naïve helper T cells to polarize into T_H_1 or T_H_2 populations. In a simplified model, these polarizations occur at opposite ends of an “immune 1-2 axis” (i1-i2 axis) of possible conditions. Additional polarizations of helper/regulatory T cells were discovered later, such as for example T_H_17 and T_reg_ phenotypes; although these polarizations are not selected by the axis-end conditions, they are affected by i1-i2 axis factors, and may retain more potential for change than the relatively stable T_H_1 and T_H_2 phenotypes. I1-i2 axis conditions are also relevant for polarizations of other types of leukocytes, such as for example macrophages. Tissue milieus with “type 1 immunity” (“i1”) are biased towards cell-mediated cytotoxicity, while the term “type 2 immunity” (“i2”) is used for a variety of conditions which have in common that they inhibit type 1 immunity. The immune milieus of some tissues, like the gills in fish and the uterus in pregnant mammals, probably are skewed towards type 2 immunity. An i2-skewed milieu is also created by many tumors, which allows them to escape eradication by type 1 immunity. In this review we compare a number of i1-i2 axis factors between fish and mammals, and conclude that several principles of the i1-i2 axis system seem to be ancient and shared between all classes of jawed vertebrates. Furthermore, the present study is the first to identify a canonical T_H_2 cytokine locus in a bony fish, namely spotted gar, in the sense that it includes *RAD50* and bona fide genes of both *IL-4/13* and *IL-3/IL-5/GM-CSF* families.

## 1. Introduction

### General Principles of the i1-i2 Axis as Exemplified by Major Polarizations of Mammalian Helper and Regulatory T Cells

Depending on the stimuli, largely in primary immune organs, hematopoietic stem cells can develop into a large array of morphologically and functionally different leukocyte populations [1,2,3]. At the sites of activation, these mature but “naïve” immune cells can then further polarize towards phenotypically distinct cell populations depending on the conditions. The polarized phenotypes can be more or less fixed by epigenetic changes, including chromatin folding, DNA methylation and histone modification [4,5,6]. Very important for polarization of immune cells is a loosely defined “axis” of conditions that favor type 1 or type 2 immunity, and which we call here the “i1-i2 axis”. In helper T (T_H_) cells the i1-i2 axis end conditions induce pronounced polarizations, T_H_1 and T_H_2 respectively, which are rather stably imprinted in the cell clones by heritable epigenetic changes [5,7,8,9,10,11]. The pronounced and stable character of T_H_1 and T_H_2 polarizations allowed their discovery already more than 25 years ago [7,8,9,12]. The type 1 end of the i1-i2 axis is represented by conditions which stimulate expression of interferon γ (IFNγ) and are enhanced by this cytokine, while for the type 2 end of the i1-i2 axis a self-stimulatory marker cytokine is interleukin 4 (IL-4; [5]). Important transcription factors for T_H_1 cells are T-bet and STAT4 [5,13,14], and important transcription factors for T_H_2 cells are GATA-3 and STAT6 [5,15,16,17,18,19]. The i1-i2 axis affects polarization of various types of leukocytes, and shifts along the axis are not only determined by IFNγ and IL-4 concentrations, but are also affected by other cytokines, pathogen-associated molecular patterns (PAMPs), danger-associated molecular patterns (DAMPs), the strength and nature of cell-cell interactions, and physiochemical variables such as the concentrations of nucleotides and their derivatives, glucocorticoids, and oxygen [5,20,21,22,23,24]. In this review we will only discuss a few relevant factors, mainly concentrating on several important cytokines and transcription factors.

**Figure 1:**
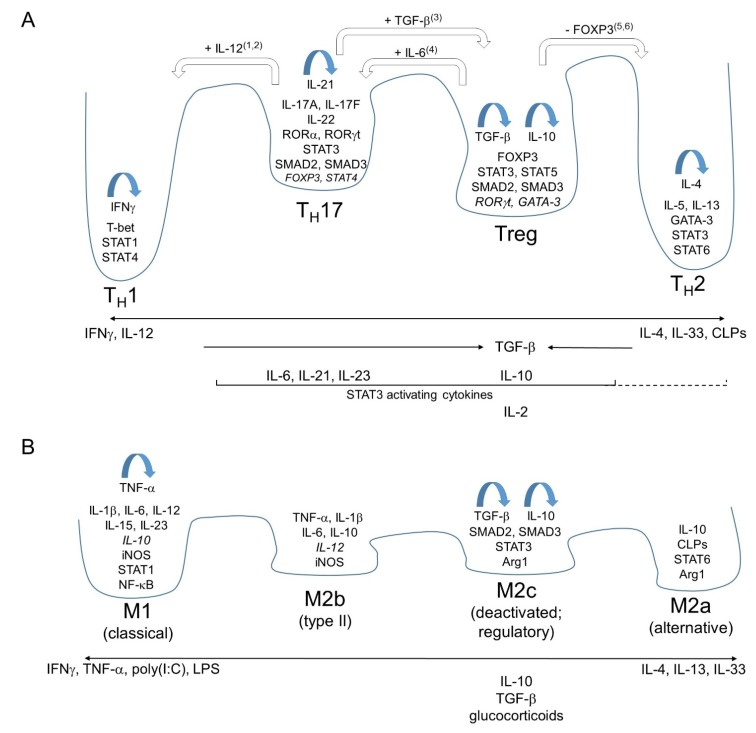
Schematic depiction of the i1-i2 axis affecting the polarizations of mammalian T_H_/T_reg_ cells (**A**) and macrophages (**B**). Only some relevant factors, and not all known polarizations, are summarized. The figure organization and purpose is explained in the main text. Italic font relates to molecules that have been described as molecules especially expressed by that respective polarized cell population, but are present in lesser amounts than in other polarized populations for which they are more characteristic. Our depictions of factors relevant to T_H_/T_reg_ cell polarizations are the summaries of mainstream ideas, with most references given in the main text. For GATA-3 expression in T_reg_ cells see [57]. For RORγt expression in early T_reg_, and FOXP3 expression in early T_H_17, see review [58]. For STAT4 expression in T_H_17 cells see [59]. The macrophage polarization figure (**B**) is importantly based on a figure by Mantovani *et al.* [60], while modifications were made based on additional literature as referenced in the main text. The names between brackets are alternative designations that have been used for the respective macrophage polarizations. The blue arrows relate to self-amplifying loops as described by: For interferon γ (IFNγ) in T_H_1 cells, see [5,13]; for IL-21 in T_H_17 cells see [45]; for TGF-β in T_reg_ see [44]; for IL-10 in T_reg_ see [43,46]; for IL-4 in T_H_2 see [5]; for TNF-α in M1 macrophages see [61,62]; for IL-10 and TGF-β in M2c macrophages see [63] and [64], respectively. The white arrows relate to experiments that described how addition (+) or repression (−) of factors could push already polarized T_H_/T_reg_ cells into another polarization state, with superscript numbers indicating the respective literature: 1, [49]; 2, [50]; 3, [53]; 4, [48]; 5, [47]; 6, [51].

The term “type 1 immunity” relates to a milieu skewed towards cytotoxic functions including enhanced natural killer (NK), T_H_1, and CD8^+^ T cell activities. The major function of type 1 immunity is to kill cancer cells or cells with intracellular pathogens. Cell killing processes can be expected to be in relative disregard of damaging host tissue, but many tissue damaging inflammations for which originally T_H_1 cells were blamed are actually mediated by T_H_17 cells [25]. T_H_17 cells are only partially shifted towards the i1-end of the i1-i2 axis (Figure 1A), and are representative for what can be called “type 3 immunity” (“i3”) [26]. Characteristic for type 3 immunity is the involvement of transcription factors RORα and/or RORγt, secretion of the cytokines IL-17A, IL-17F and IL-22, and the activation of neutrophils [26,27,28]. Type 3 immunity has an important function in protection against extracellular bacteria and some fungi. In the healthy intestine, T_H_17 cells form an important role in a complex network of interactions between commensal bacteria and immune cells and help to maintain tissue homeostasis and barrier integrity [29,30,31]. Although phenotypically T_H_17 polarizations are often considered to be more plastic than T_H_1 and T_H_2 polarizations, some epigenetic modifications acquired during T_H_17 polarization are rather stable [32].

The use of the term “type 2 immunity” can somewhat differ between researchers and research fields, but tends to encompass both milieus with dominant immunosuppressive functions, for which TGF-β and IL-10 are marker molecules, and inflammatory milieus with dominant functions of cytokines IL-4, IL-5 and/or IL-13. Characteristic for type 2 inflammation are anti-parasite activities involving the activation of mast cells and eosinophils, and the secretion of IgE by B cells. In allergy diseases, these types of reactions are triggered by allergens. Generally, i2-skewed immune milieus may be more protective of tissues than i1-skewed milieus [33,34], which also agrees with type 2 immunity being important in wound healing [23,35]. However, also type 2 immune reactions can cause considerable tissue damages (e.g., [36]), amongst which tissue fibrosis [37]. The stimulation of T_H_2 polarization by the alarmin IL-33, which is released from damaged tissue, can be understood from the importance of type 2 immunity in tissue regeneration and wound healing [23,35,38]. Similarly, the expression of chitinase-like proteins (CLPs), which is enhanced by helminth infection or injury, also induces T_H_2 responses, although CLPs can also stimulate IL-17 release [39,40,41,42].

Figure 1 is our attempt to summarize some principles in cell polarization as they have been described for mammals. The horizontal axis relates to the concentrations of some important factors, whereas the vertical depiction of “energy valleys” relates to the relative stability of a cell polarization; the depiction with energy valleys only serves to explain a model, and the depicted valley depths have no absolute meanings. Some of the molecules characteristically expressed by the respective polarized cells are listed within those valleys, with blue arrows highlighting the cytokines that help fixing the cell phenotype as part of self-stimulatory loops [5,13,43,44,45,46]. White arrows refer to studies that described how some already polarized phenotypes are plastic in that they can be modified towards other polarizations; our figure is a simplification in the sense that the cell types produced by this type of route can be somewhat different from those directly produced from naïve T cells [47,48,49,50,51,52,53]. Our choice of the white arrows in Figure 1, with numbers for references described in the figure legend, represents our attempt to summarize major literature, and these possible conversions highlight similarities between polarizations in the order that they are depicted as “neighbors” in Figure 1. Readers should, however, realize that also conversions between “non-neighboring” (defined by Figure 1) polarizations have been reported possible (not shown in Figure 1; e.g., [54]), underlining that the Figure 1 depiction is only a model which explains some but not all principles of immune polarization. Importantly, though, the continuous axis-nature of the polarizations as depicted in Figure 1 is also supported by shared expressions of some marker molecules between “neighbors”, with typically in one of the populations the expression being considerably lower or restricted to subpopulations (indicated by italic font in Figure 1). Naturally, Figure 1 is an enormous simplification, describing only a few major factors and categorizing only a few major cell populations. Especially T lymphocyte types with regulatory functions are a complex set of cells widely distributed along the i1-i2 axis [55], and the single regulatory T cell valley in Figure 1A is only representative for major sets of T_reg_ cells. The stability of T_reg_ polarizations is believed to differ between subtypes [56].

In Figure 1, the actual biological situation of polarizations would probably be better represented by a three-dimensional energy landscape with many hills, ridges and valleys [65,66], where the 1-2 axis might be something like the East-West axis and with multiple possible routes between the East and West sides, and further addition of dimensions would further improve the figure; however, that would need a lot more information than currently available.

In Figure 1A we did not include T_H_9 and T_FH_ cells because currently it is impossible to discuss their possible presence in the context of fish because relevant genes (e.g., IL-9) or tissues (e.g., functional equivalents of lymph nodes) have not been found/clarified in fish yet. It is very likely that fish do not have the exact same immune cell polarizations as found in mammals, although the major principles of the i1-i2 axis appear to be the same as we argue in this article. If we somewhat freely interpret the work by Kaplan and co-workers [67], those authors arranged mammalian T_H_9 and T_FH_ cells in the order T_H_1-T_FH_-T_H_17-T_reg_-T_H_9-T_H_2 along the i1-i2 axis. However, others have found similarities between T_FH_ and T_H_2 cells [68] or stressed the heterogeneity and the existence of subpopulations among T_FH_ cells [69]; probably the adaptation of cells to T_FH_ function should not be understood as a unique polarization along the i1-i2 axis.

TGF-β limits how far cells polarize along the i1-i2 axis in either direction, and it is an important cytokine for the development of T_H_17 and T_reg_ cells. A higher concentration of TGF-β favors development of T_reg_ over that of T_H_17 [70,71]. Whereas STAT3 activation in T_H_17 cells is especially enhanced by IL-6, IL-21 and IL-23, the STAT3 activation in T_reg_ cells is especially enhanced by IL-10 [46]. It is tempting to speculate that the relatively common transcription factor STAT3 [72] blocks development of the “more extreme” axis-end phenotypes T_H_1 and T_H_2. However, although STAT3 is known to suppress expression of T_H_1 marker genes [73], it was reported necessary for T_H_2 development [74]. Because the dependency of T_H_2 cells on STAT3 has not been studied intensively, we used a dashed line in Figure 1A for the yet better to be clarified main factors that stimulate STAT3 in T_H_2 cell development.

Although STAT5 activity can stimulate survival and proliferation of different sets of lymphocytes [75], it represses T_H_17 differentiation and shifts the development of common T_H_17/T_reg_ precursor cells towards T_reg_ [76]; the important inducer of STAT5 activity in T_reg_ is IL-2, a cytokine which can also stimulate other lymphocyte populations [77]. The Figure 1 model does not include the STAT5 activity enhancers IL-9 and thymic stromal lymphopoietin (TSLP), which both stimulate type 2 immunity [38], because these two genes have not been found in fish (yet). STAT5 activities in NK cells and CD8^+^ T cells (not shown in Figure 1), important for type 1 immunity, can be induced by IL-15 that is expressed by dendritic cells or monocytes/macrophages [77].

Although most researchers will agree that polarizations of immune cells depend both on various gradients of factors as well as on more discrete sets of conditions, to try to catch that in a figure with only a single axis as in Figure 1 could be righteously considered presumptuous, overly simplified, and misleading. However, we argue that in such it doesn’t stand out negatively from more popular figures trying to summarize leukocyte polarizations. Furthermore, we argue that if we wish to compare immune polarizations of different cells and tissues, of healthy *vs.* diseased conditions, and among species as diverged as mammals and fish, we need a kind of articulated bird-view of the i1-i2 axis as attempted in Figure 1. In the current study we use the Figure 1 model for analyzing published data in fish, and conclude that the immune systems of mammals and teleost fish seem to obey to at least some similar i1-i2 axis principles.

## 2. Polarizations along the i1-i2 Axis of Mammalian Leukocytes Other than Helper and Regulatory T Cells

Polarizations towards type 1, type 3 and type 2 immunity, which are very reminiscent of the ones found for T_H_ cells, have been described for innate lymphoid cells (ILCs) (reviews [26,78,79]). Marker molecules expressed by ILC1 cells are transcription factor T-bet and cytokine IFNγ, marker molecules expressed by ILC3 cells are transcription factor RORγt and cytokines IL-17 and IL-22, and marker molecules for ILC2 cells are transcription factor GATA-3 and cytokines IL-5 and IL-13. The intermediate position of ILC3 along the i1-i2 axis, similar to as found for T_H_17 cells, is supported by sharing of some marker transcription factors and cytokines with either ILC1 or ILC2 cells, while ILC1 and ILC2 cells appear to lack unique overlaps with each other [26]. ILC3 cells can be converted into ILC1 cells by stimulation with IL-12, resulting in downregulation of RORγt and upregulation of T-bet [80]. Some difficulties in classification of ILCs are caused by the existence of multiple ILC1-type populations, and by differences in their regulation between human and mouse [78]. Most researchers do not distinguish a separate “ILC_reg_” population, but besides aiding type 2 inflammation, ILC2 cells are known to have important functions in tissue homeostasis and tissue repair [81,82]. Very interestingly, recently also ILC3 subsets were found to have T_reg_-like functions in the sense that they could negatively select antigen-specific T cells [83]. Thus, like found among T cells, among ILCs there is an overlap between type 1 and type 3 immunity, between type 3 immunity and regulatory functions, and between regulatory functions and i2 inflammation.

Except for regulatory/helper T and ILC populations, i1-i2 polarizations similar to the ones listed above because involving at least several of the same marker molecules have been reported for CD8^+^ T cells [84], B cells [85], neutrophils [86] and dendritic cells [87]. However, it is beyond the scope of this article to discuss those polarizations. Macrophage populations, on the other hand, will be discussed here, because macrophage polarizations have been studied relatively intensively and are of major importance in the creation of immune milieus and in tissue modeling. Furthermore, there are some functional data on macrophage polarizations in teleost fish (see further below).

In Figure 1B we made an attempt to characterize major polarizations of mammalian macrophages along the i1-i2 axis. The figure is a modified version from a distribution figure by Mantovani *et al.* [60], and as in Figure 1A, the depths of the “energy valleys” only serve to explain a model and have no absolute meanings. Very importantly, what emerges as a general impression from literature is that macrophage populations have less pronounced self-amplifying loops (although for an autocrine TNF-loop see [61] and [62], for an autocrine IL-10 loop see [63], and for an autocrine TGF-β loop see [64]) than known for T_H_/T_reg_ polarizations, and that macrophage polarizations appear to be rather unstable and hence a rather direct reflection of their immune environment [88,89]. This makes sense since in contrast to T cells which are antigen-specific and whose epigenetic modifications contribute to immune memory [19,90], macrophages interact with a large number of antigens. Their plasticity probably is an important reason why macrophage polarizations were discovered later and remain poorer characterized than T_H_/T_reg_ polarizations. Many researchers only distinguish between M1 and M2 macrophages, without further subdivisions.

Macrophages are sensitive to DAMPS and PAMPs. LPS is an important PAMP for shifting macrophage polarizations towards the i1-end of the i1-i2 axis, and can stimulate the development of both M1 and M2b macrophages [60,89]. Viral dsRNA mimic poly(I:C) also induces M1 polarization [91,92]. M1-skewed macrophages express IL-12 which is important for the initiation of T_H_1 polarization [89], and they also express IL-15 [93,94] which is especially important for the stimulation of NK and CD8^+^ T cells [77,95]. Both M1 and M2b macrophages express inducible nitric oxide synthase (iNOS) and are active in clearance of bacteria through NO production [94,96]; the big difference of M1 *vs.* M2b cells is the abundant production of IL-12 *vs.* IL-10, so that M1 cells support type 1 immunity and M2b cells are able to support type 2 immunity [96,97,98]. The expression (-pathways) of iNOS and arginase affect each other negatively [99,100,101]. Expression of arginase in M2c and M2a cells leads to production of ornithine, a precursor of extracellular matrix components that contributes to wound healing [60,102]. M2a and M2c macrophages appear to participate in tissue regeneration following tissue injury [103], which explains why M2a polarization can be enhanced by alarmin IL-33 ([104,105]. We did not include alarmin IL-25 (alias IL-17E) in Figure 1, although it also supports type 2 immunity, because of difficulties to find an orthologue in fish [106,107]. M2a polarization involving i2 cytokines induces macrophages to express CLPs [40,108,109]. M2c macrophages have anti-inflammatory properties and are stimulated by glucocorticoids, IL-10 and TGF-β [110,111]. It is of note that in human, different from the mouse situation, some M2 polarizations may not be typified by high levels of arginase expression [112,113].

In many tumors the tumor cells attract monocytes/macrophages and skew their development towards type 2 immunity; the M2 macrophages then in reciprocal interaction with the tumor cells remodel the tumor microenvironment, which aids the tumor cells and protects them from type 1 immunity [114,115]. The current successes in cancer immunotherapies are largely based on shifting the tumor milieu from type 2 towards type 1 immunity, and one of the focuses of investigation concerns macrophage polarizations (e.g., [116,117]).

## 3. Fish Orthologues of Mammalian genes for i1-i2 Axis Functions

**Figure 2:**
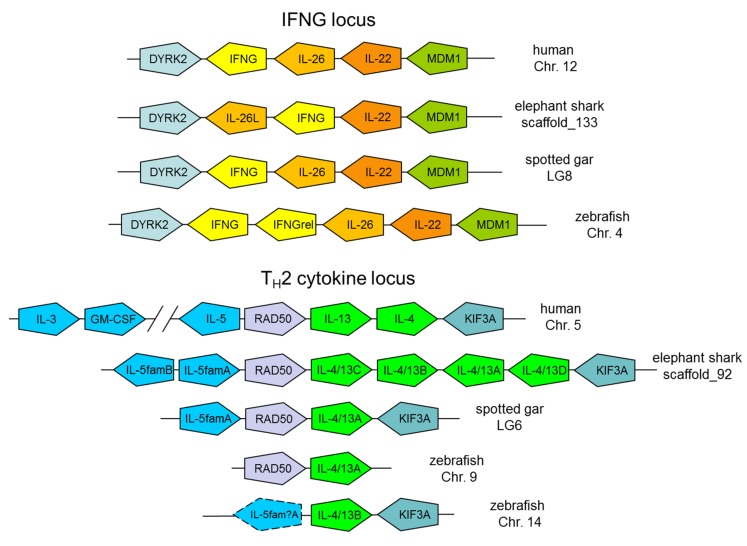
Schematic depiction of the conservation of the IFNG and T_H_2 cytokine loci in fish and mammals. The pentagon orientations correspond with gene directions. Depicted gene organizations are based on analysis of genomic sequence information available for elephant shark (*Callorhinchus milii*) provided by the Elephant Shark Genome Project ([118] and GenBank accession number AAVX02000000) and for the other species in the following datasets of the Ensembl database [119]: human (*Homo sapiens*), GRCh38.p2; spotted gar (*Lepisosteus oculatus*), LepOcu1; zebrafish (*Danio rerio*), GRCz10. Between human IL-5 and GM-CSF lays a 465 kb stretch with a number of genes which are not shown in this figure. Most of the depicted gene organizations have been described before [107,120,121,122,123,124,125]. The deduced elephant shark IL-26L amino acid sequence is MRCAAACLLVSLGVCVVRTSTATCKPKVSDRLIQDFIRCVGNVMNASQHYWGSSWSDGKGYRFLPKPVKMTKHGKCTVVKKALEFYLIFLKQYRPMPDGFKQDLIKVKHYLEEMYAKTRCDECKSSKDLNAERAIKRLEKEICKARCSKHTSVTKKSIIFQLYILRNLITNMA. For the deduced encoded elephant shark T_H_2 locus cytokine sequences see Table S3 (except for IL-4/13D these are also described in [124]). For the spotted gar IL-4/13A sequence we refer to [125]. The deduced spotted gar IL-5famA sequence is MSMYLVLLILGVHYSGVRTQHYHFISEIISHIENAKQGVVHTILLTPQNVLNANCTASYSKIFLKGIKHLSVHSEHGSQEELKLIIHNMERMDVICPNLKHQVPDCEVQDTSTFQFLRQFTKFLQKIKRSDCFRLRSEYPFSA, which is compared with other sequences in Figure 3.

For all the mammalian genes encoding the proteins shown in Figure 1, homologues could be found in ray-finned and/or in cartilaginous fish, in most cases including probable orthologues. Examples are shown in Figure 2 plus supplementary file 1.

For teleost fish now a relatively large number of “whole genome” sequences have been published. However, for cartilaginous fish, the only species for which the sequence of a large part of the genome has been published is the chimaera elephant shark (*Callorhinchus milii*; [107]); hence, gaps in the published elephant shark genome can’t be “filled in” with information from other cartilaginous fish.

Nevertheless, for most of the molecules depicted in Figure 1 probable gene orthologues can be found in the published elephant shark genomic sequences, and for the exceptions the incomplete nature of the published sequences might be to blame. It has been argued that elephant shark does not have an *RAR-related orphan receptor* (*ROR*) *gamma* gene [107]. That may be true, although such conclusion would need full genome sequence information, and in our preliminary phylogenetic tree analyses (data not shown) the molecule encoded by the elephant shark “*RORA-like”* (*RORAL*) gene at scaffold 2358 (supplementary file 1) clusters with RORγ sequences. A serious analysis of the evolution of the ROR family of transcription factors would need a more serious effort than feasible within the scope of this article. Regardless, because in mammals not only *RORC* but also *RORA* can contribute to T_H_17 development [28], the question on *C* or *A* identity might not be so relevant across these wide species borders when addressing the possibility of T_H_17 polarization.

An early ancestor of all extant teleost fishes experienced a whole genome duplication event [126], and several teleost fish lineages experienced an additional genome duplication event (e.g., [127]). This was frequently followed by gene losses, lineage specific gene duplications and/or translocations, causing a tendency for the analysis of orthologous relationships between mammals and teleost fish to be more complicated than between tetrapods, cartilaginous fish and non-teleost primitive bony fish. However, overall and in principle, many gene organizations in teleost fish resemble those of mammals and elephant shark [107,126], and it was because of conserved syntenies that we and others could identify teleost fish genes for small cytokine genes despite their poorly conserved sequences (e.g., [120,121,122,128,129]). A number of the gene syntenies between fish and human depicted in Figure 2 and supplementary file 1 were already described earlier (e.g., [107,130]), but we feel it is convenient for the readers to have the data presented together.

Figure 1 and Figure 2, and supplementary file 1 do not provide information regarding the relevant receptors and neither regarding many of the pathway molecules, because we argue that the currently depicted molecules are sufficiently representative for their functional pathways. However, it is important to realize that also the relevant cytokine receptor and pathway molecules tend to be rather well conserved between fish and mammals (e.g., [107,131,132,133]). Furthermore, regarding the molecules depicted in Figure 1, we did not analyze the genomic locations of fish genes involved in glucocorticoid pathways and of fish CLP genes; for these genes we refer interested readers to references [134] and [135], respectively.

As a negative exception among the proteins depicted in Figure 1, for IL-33, which is a highly diverged member of the IL-1 family with poor sequence conservation even between mammals and birds (see Ensembl accession ENSGALG00000020558), we could not find a likely gene candidate in any of the investigated fish species. However, in teleost fish multiple IL-1 family members have been found [136,137], and teleost genes have been annotated as *IL1RL1* (alias *ST2* or *ST2L*; [136,138,139]) which in mammals encodes the receptor for IL-33. Since *IL1RL1* maps to a locus with multiple similar genes of the IL-1 receptor family [140], this *IL1RL1* designation in fish probably would need a more intensive analysis than has been published to date or is feasible within the scope for our present study. In short, fish may have IL-33 (-receptor) function, but there is no real evidence to support that.

From the early days that we started to identify i1-i2 axis cytokine genes in fish despite of their very poorly conserved sequences with the help of gene syntenies (e.g., [120,122,129]), we have been fascinated by the high conservation of loci between fish and mammals, often even in simple 1:1 orthologies. The high level of evolutionary conservation of the genomic organization of many of the i1-i2 axis gene loci, as shown in Figure 2 and supplementary file 1, contrasts with the abundant locus turnovers and copy number differences found for other genes of the immune system, like for example genes encoding MHC molecules [141,142], chemokines [143], and type I interferons [144]. For several NK cell receptor families there may not be close relatives in fish at all [145]. That many of the gene loci important for the i1-i2 axis are so well conserved between jawed fish and mammals strengthens the idea that in all jawed vertebrates major principles of the i1-i2 axis system have been conserved as core mechanics of their immune system. Our attempts to find i1-i2 axis genes in published sequences of jawless fish (lampreys, hagfish) and invertebrates proved to be difficult/impossible (data not shown), and future careful analyses should determine if to any extent some principles of the i1-i2 axis might be present in those species. For reviews on the immune systems of jawless fish, which are fundamentally different from those in jawed vertebrates, we refer to [146,147,148].

## 4. Conservation of the IFNG and T_H_2 Cytokine Loci

Very important in the T_H_1 and T_H_2 polarizations are their divergent epigenetic modifications of the IFNG and T_H_2 cytokine loci [5,149]. Especially the pronounced modifications of the T_H_2 cytokine locus, including chromatin refolding, have received a lot of attention [4,18,150,151,152,153]. Binding of transcription factor GATA-3 and STAT6 induces chromatin refolding by inducing interactions between (inter-) gene regions of *IL-5*, *RAD50*, *IL-4* and *IL-13* [18,19]. It is fascinating to see how well the IFNG and T_H_2 cytokine loci have been conserved between fish and mammals (Figure 2). The name *IL-4/13* is used for genes related to tetrapod *IL-4* and *IL-13*, because it can’t be decided to which of the two tetrapod genes the fish genes are closer related, and the gene duplication leading to *IL-4 vs. IL-13* may have occurred after the separation between the ancestors of tetrapods and ray-finned fish [122].

Most of the gene organizations shown in Figure 2 have been reported before [107,120,121,122,123,124,125], but different from previous publications [107,124,125] we found (i) *IL-26-like* (*IL-26L*; for the encoded sequence see the Figure 2 legend) in the elephant shark IFNG locus, (ii) an extra *IL-4/13* copy, *IL-4/13D*, that we had missed [124] but which was properly annotated as an *IL-4/13* gene by automated database gene prediction (XM_007902044) in the elephant shark T_H_2 cytokine locus, and (iii) an *IL-5 family* (*IL-5fam*) member in the spotted gar T_H_2 cytokine locus (for the encoded sequence see the Figure 2 legend). Our analysis of the spotted gar T_H_2 locus constitutes the first identification of a bony fish T_H_2 cytokine locus that includes *RAD50* and seemingly bona fide genes of both *IL-4/13* and *IL-3/IL-5/GM-CSF* families.

Expression of elephant shark *IL-26L* was confirmed by sequence read archive (SRA) database reports (data not shown), and the gene has an intron-exon organization typical of the IL-10 family (to which also IL-22 and IL-26 belong). Phyre^2^ software [154] predicts that elephant shark IL-26L protein has multiple α-helices, and that its structure is similar to IL-10 (confidence 52%). Although the deduced molecule does not have a typical IL-10 signature motif in the carboxy-terminal α-helix, it shares some specific cysteines with IL-26 (data not shown), and despite the minimal similarity we considered “IL-26L” to be the best possible name.

**Figure 3:**
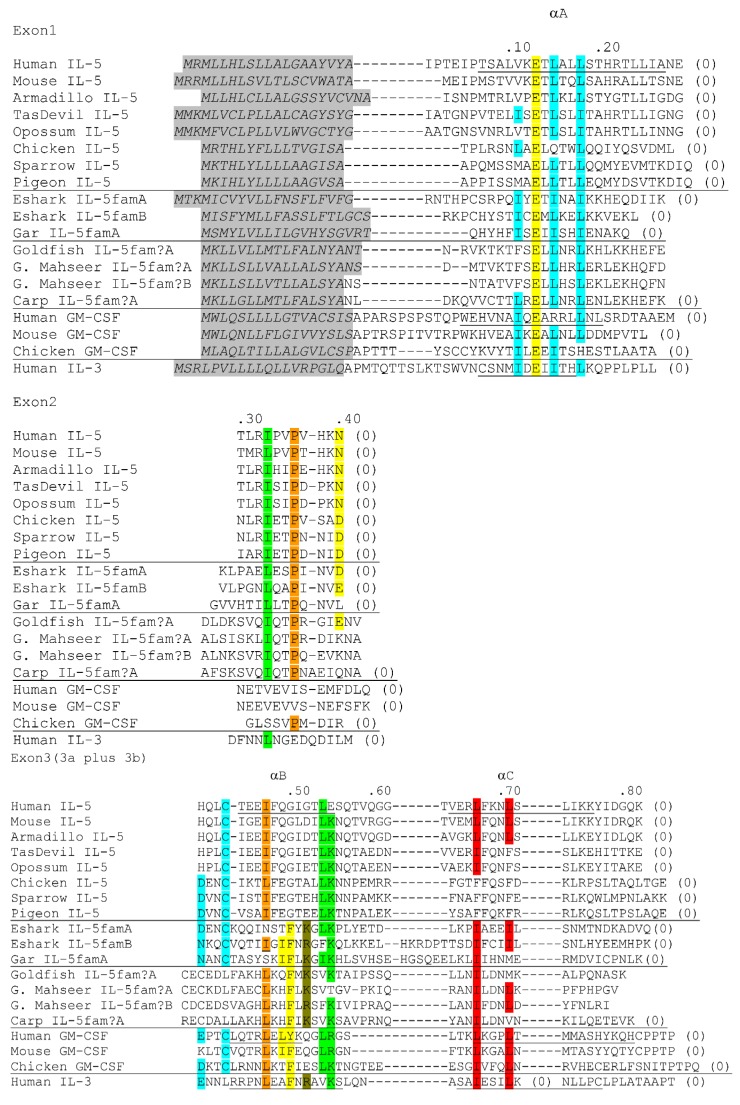

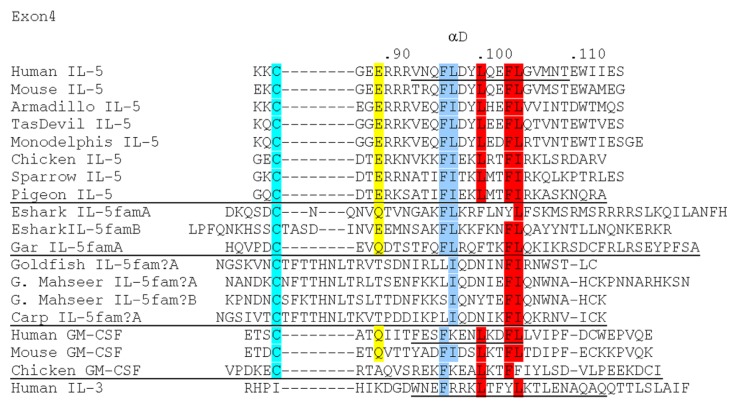
Alignment of (deduced) IL-3/IL-5/GM-CSF family member amino acid sequences. (Predicted) leader peptides are indicated with gray shading; for predictions SignalP software was used (http://www.cbs.dtu.dk/services/SignalP/). The alignment is organized according to the matching exons, and brackets relate to intron positions with the number indicating the intron phase. The α-helices αA-to-αD, of human IL-5, GM-CSF and IL-3 are indicated by underlining following [157], [158], and [159], respectively. Sequences were aligned by hand, based on considerations regarding structure and evolution (as in [122], although we made some different choices now). For the alignment among the core regions of human IL-3, IL-5 and GM-CSF we mostly followed the structural alignments by [159] and [160], with as notable exception the α-helix B sequences of IL-5 in which we introduced a gap for a better match of sequence identities with the other cytokines. Readers should realize that alignments of such highly differentiated sequences remain discussable. Conserved motifs are highlighted by different color shading in a somewhat instinctive and random manner. Some of the highlighted motifs can also be found in cytokines not belonging to the IL-3/IL-5/GM-CSF family, but not in this combination (compare with [122,160,161]. The yellow shaded glutamic acid in α-helix A is important for function [162,163,164], and, at least for GM-CSF, for binding the common β receptor chain [165]. Aligned, in that order, are the following sequences: Human (*Homo sapiens*) IL-5, GenBank accession NP_000870; mouse (*Mus musculus*) IL-5, NP_034688; nine-banded armadillo (*Dasypus novemcinctus*), XP_004456511; Tasmanian devil (*Sarcophilus harrisii*) IL-5, XP_003756529; gray short-tailed opossum (*Monodelphis domestica*) IL-5, XP_001371840; chicken (*Gallus gallus*) IL-5, ADL28818; white-throated sparrow (*Zonotrichia albicollis*) IL-5, XP_005483812; rock pigeon (*Columba livia*) IL-5, EMC79983; elephant shark (*Callorhinchus milii*) IL-5famA and IL-5famB, see supplementary file 2 and [124]; spotted gar (*Lepisosteus oculatus*) IL-5famA, see the Figure 2 legend; goldfish (*Carassius auratus*) IL-5fam?A, GBZM01010380; golden mahseer (*Tor putitora*) IL-5fam?A and IL-5fam?B, see supplementary file 2; common carp (*Cyprinus carpio*) IL-5fam?A, compare LN591230 or LHQP01003280 with the goldfish or golden mahseer sequences; human (*Homo sapiens*) GM-CSF, NP_000749; mouse (*Mus musculus*) GM-CSF, CAA26192; chicken (*Gallus gallus*) GM-CSF, NP_001007079; human (*Homo sapiens*) IL-3, AAH66275.

In a previous paper we depicted the elephant shark *IL-5famA* and *IL-5famB* genes as “*IL-5A*” and “*IL-5B*”, upon request of the respective journal who considered the “fam” indication (for family) to be confusing for a general audience ([124]; the “fam” designations were only given as optional in the supplement of that publication). But, although the genes are related to *IL-5*, and are situated at the expected *IL-5* location, we have no strong arguments for their closer relation to *IL-5* than to *IL-3* or *GM-CSF* (for discussion of the evolution of the T_H_2 cytokine locus see also [122]). So in the current article we like to use the nomenclature including “*fam*”, although both nomenclatures are defendable. The cytokine family including IL-3, IL-5 and GM-CSF is characterized by extremely poorly conserved sequences, especially among the IL-3 molecules [155], with only a few typical and conserved sequence motifs (Figure 3). Whereas hitherto for bony fish no convincing IL-5-family candidates were reported, we now found such gene at the expected *IL-5* site in the spotted gar T_H_2 cytokine locus which we designated *IL-5famA* (Figure 2 and Figure 3). It has the family-typical intron-exon organization, and in contrast to the other detected IL-5fam molecules in fish, gar IL-5famA has a cytokine as top-match upon blastp comparison with the NCBI database (Genbank accession KFO26617); the relevant unknown cytokine gene appears to be correctly predicted for cattle (GenBank accession XP_010796657), maps directly downstream of mammalian *IL-3* and *GM-CSF*, is predicted to encode multiple α-helices according to Phyre^2^ software, and appears to have pseudogene identity in humans (data not shown). We may discuss this hitherto unknown mammalian cytokine in more detail in a future publication, and only mention it here as additional evidence that the fish IL-5fam molecules truly belong to the IL-3/IL-5/GM-CSF family.

In teleost fish gene candidates for the common β receptor chain (alias IL-3Rβ) have been known for a long time [131], and accordingly the finding of IL-3/IL-5/GM-CSF family genes has been anticipated. However, the best that we could do so far was a zebrafish gene with unclear signature which we designated “*IL-5?*” [122] and which we now in Figure 2 designate as “*IL-5fam?A*”. We actually are still insecure whether this somewhat peculiar zebrafish gene is an intact gene, as it may not have a normal exon1 sequence (data not shown), and therefore it is indicated by a dashed line in Figure 2. However, we are now confident that the zebrafish gene is at least related to intact cytokine genes, as orthologous and apparently bona fide cytokine genes can be found in carps and goldfish. Like in zebrafish, in common carp the gene is also linked with *KIF3A* (GenBank accession LN591230). Figure 3 shows four cyprinid IL-5fam? sequences, namely the LN591230 encoded common carp sequence, a goldfish sequence encoded by GenBank TSA accession GBZM01010380, and two very similar golden mahseer sequences assembled from SRA reads (data not shown; we don’t show the individual SRA accessions). The question mark in the “IL-5fam?” nomenclature expresses our insecurity about the molecule identities, because whereas in our opinion the gar and elephant shark molecules have a convincing IL-3/IL-5/GM-CSF family signature, the only partially conserved signature in cyprinid sequences fails to convince us (Figure 3). Nevertheless, because of genomic location and lack of better matching candidates, the most likely hypothesis appears to us that these cyprinid sequences are highly diverged members of the IL-3/IL-5/GM-CSF family. To our frustration, even with the knowledge of the gar and cyprinid IL-5fam(?) sequences, we have been unable so far to find any IL-3/IL-5/GM-CSF family gene candidates in non-cyprinid teleosts. Although negative findings for gene members of these small cytokine families with poorly conserved sequences shouldn’t be overvalued (see [107] and [124]), it might be speculated based on the lack of convincing gene candidates that the importance of the IL-3/IL-5/GM-CSF family was reduced in teleost fish compared to other classes of vertebrates. Functional analyses of the fish molecules, including their possible interaction with the common β receptor chain, should clarify these matters.

As a general statement based on our many years of experience in identifying genes of the immune system, we feel that at the genetic level the immune systems of elephant shark and gar have more similarities with the mammalian immune system than found between teleost fish and mammals. Slow evolution towards the elephant shark genome and rapid evolution towards the extant teleost fish genomes have been noted before [107,126,156]. When fish research is performed with the aim to deduce the ancestral features of the human immune system, it might be worth considering to move research away from teleosts to for example gar (*Lepisosteus oculatus*).

## 5. Investigation of Tissue-Specific Co-Expressions of T_H_1, T_H_17, T_reg_ and T_H_2 Signature Genes in Fish; Gills Consistently Express High Levels of T_H_2 Signature Genes

Previously we showed that in salmonid fishes the expression of *GATA-3* and *IL-4/13A* are high in gill, skin and thymus, also in relation to other genes of the immune system, and we assumed that these tissues are skewed towards type 2 immunity [166]. Similar findings for high GATA-3 and IL-4/13A expression in teleost gill were also made by others [121,167,168]. In the current study we extended this type of investigation with database mining, in which we blasted sequences against tissue-specific single SRA datasets available for healthy individuals of several fish species (http://www.ncbi.nlm.nih.gov/sra) (data not shown) as summarized in Table 1. We found our previous observations of high *IL-4/13A* and *GATA-3* expression in rainbow trout and Atlantic salmon gills confirmed by transcriptome analyses for these two species, but also for pike (like salmonids member of *Protacanthopterygii*) and golden mahseer (a cyprinid fish) (Table 1). Excitingly, even in elephant shark the *GATA-3* and *IL-4/13* expressions appear to be particularly high in gill, coinciding with high *IL-5fam* gene expression, which suggests that the elephant shark IL-5fam/RAD50/IL-4/13 locus is a similar GATA-3 driven T_H_2 cytokine locus as present in mammals, and that the i2-skewage of the gill immune milieu is ancient. More analysis would be necessary to determine how the i2-skewage is distributed over the gill, and to what extent it maps to interbranchial lymphoid tissues (ILT; [169]). Interestingly, in Golden mahseer, the expressions of *IL-4/13B* and *IL-5fam?* genes are not tightly associated with those of *IL-4/13A* and *GATA-3*, and are not consistently although often high in gill (Table 1 and data not shown). This suggests that only one of the two teleost copies of the T_H_2 cytokine locus resulting from the teleost ancestral whole genome duplication, namely the *RAD50* + *IL-4/13A* locus (Figure 2; see also [122]), retained the expression mode of the ancestral T_H_2 cytokine locus. In accordance, in the promoter regions of teleost *IL-4/13A* genes and not in those of teleost *IL-4/13B* genes we found a rather well conserved GATA-3 binding motif [122]. In elephant shark *STAT6* expression is highest in gill, as expected from an i2-skewed tissue, but for unknown reason in teleost fish it does not tightly associate with the high *GATA-3* and *IL-4/13A* expression found in gill (Table 1).

Besides a clustering of high expressions of T_H_2 signature genes in gill, the investigated elephant shark individual displays such clustering for T_H_1 signature genes in its spleen and for T_H_17-signature genes in its intestine (Table 1). Although this appears very interesting, and may be indicative of ancient tissue-specific immune biases, these data do need confirmation in other cartilaginous fish individuals before allowing conclusions.

In the investigated teleost fish individuals, besides the consistent link between *GATA-3* and *IL-4/13A* expression, we also found a consistent link between high *STAT1* and *STAT4* expression. The highest *STAT1* and *STAT4* expressions correlated relatively well with the highest *T-bet* (alias *TBX21*) expression, but the tissue of highest expression differed among the investigated teleosts and there was no clear correlation with *IFNG* expression (Table 1). Whether the lack of consistencies seen in Table 1 represent genuine differences between species or are due to random differences between fish individuals or between sampling techniques, can’t be decided without further investigation. However, it is of note that, for example, in another study comparing among healthy trout and salmon individuals we also found considerable variation regarding the tissue of highest *IFNG* expression [166].

In the teleost fish turbot the highest expression of both *IL-17A/F* and *IL-22* was found in the intestine [170], which would agree with the findings in elephant shark shown in Table 1. However, before concluding that the fish intestinal immune milieu—or part of it—tends to be i3-skewed, more research is needed, and the data of the teleost fish individuals summarized in Table 1 argue against it.

For signature genes of regulatory functions such as *FOXP3*, *IL-10* and *TGF-*β, we could not distinguish any notable expression patterns among healthy tissues of either elephant shark or teleost fish (Table 1).

Table 1Expression levels of immune signature genes in various tissues determined by BLAST analysis against cartilaginous and teleost fish sequence read archive (SRA) datasets. Read numbers per 5 × 10^7^ reads of various immune signature genes of cartilaginous and teleost fish species were determined by similarity searches against tissue-specific SRA datasets (from http://www.ncbi.nlm.nih.gov/Traces/sra/ (data not shown); see Table 1C) using the BLAST search function at NCBI (http://blast.ncbi.nlm.nih.gov/Blast.cgi). ORF sequences (see supplementary file 2) were subjected to “Megablast” analysis (blastn) using default software settings except that the “max target sequences” number was changed to 20,000 and that the “word size” number was changed to 64. To ensure specificity of the Megablast analysis, only matches with “score” values ≥128 for elephant shark, ≥251 for golden mahseer, ≥168 for northern pike and Atlantic salmon, and ≥169 for rainbow trout, were counted. Colored backgrounds highlight the tissues with the highest relative expression of the respective gene, and the red frames highlight the consistent high expression of *IL-4/13A* and *GATA-3* in teleost gills.

**Table:** **A.** Cartilaginous fish

		Elephant Shark (*Callorhinchus milii*)			
		Gill	Kidney	Spleen	Intestine
T_H_1-signature	T-bet	4	1	121	3
	STAT1	6006	701	5263	2168
	STAT4	726	131	3873	738
	IFNγ	17	1	78	6
T_H_17-signature	IL17A/F1	1	0	0	7
	IL17A/F2	21	0	2	66
	IL-21L	0	0	3	2
	IL-22	4	0	0	15
T_reg_-signature	Foxp3	10	1	51	3
	IL-10	7	4	185	13
	TGFβ1	72	36	66	3
T_H_2-signature	GATA3	2287	92	575	95
	STAT6	290	41	199	102
	IL-4/13A	3	0	0	0
	IL-4/13B	8	0	0	1
	IL-4/13C	0	1	0	0
	IL-4/13D	0	0	0	0
	IL-5A	0	0	0	0
	IL-5B	3	0	0	0

**Table:** **B.** Teleost fish (bony fish)

		Golden mahseer (*Tor putitora*)			Northern pike (*Esox lucius*)					Rainbow trout (*Oncorhynchus mykiss*)				Atlantic salmon (*Salmo salar*)			
		Gill	Kidney	Spleen	Gill	Kidney	Head Kidney	Spleen	Intestine	Gill	Kidney	Spleen	Intestine	Gill	Kidney	Spleen	Intestine
T_H_1-signature	T-bet	4	47	54	91	114	663	899	28	12	20	22	11	134	320	1434	66
	STAT1_1	523	1174	42	2589	1555	3200	2579	930	2041	2219	5794	2422	5608	5386	10317	2530
	STAT1_2									1990	1870	3563	1878				
	STAT4_1	1525	1714	686	1866	1375	3702	2708	785	822	964	1122	679	1611	729	2273	1916
	STAT4_2									484	37	62	88				
	IFNγ_1	85	47	31	8	0	4	11	1	19	0	189	84	6	6	32	2
	IFNγ_2									17	0	211	99				
T_H_17-signature	IL-17A/F1a	14	21	13	13	15	2	0	2	14	5	1	9	0	5	0	0
	IL-17A/F1b									1	6	0	4	5	11	0	0
	IL-17A/F2a	11	6	4	10	0	0	0	0	4	5	0	2	8	39	0	0
	IL-17A/F2b									3	0	0	2	1	0	0	2
	IL-17A/F3	6	10	6	75	7	7	4	1	3	0	2	0	2	1	2	1
	IL-21	8	28	19	53	35	26	14	123	1	0	0	6	0	0	0	0
	IL-22	2	4	1	15	2	0	5	2	13	0	11	5	15	5	7	4
T_reg_-signature	Foxp3_1	11	12	9	100	62	257	138	32	61	11	28	35	230	143	666	149
	Foxp3_2									115	25	43	63				
	IL-10	32	179	130	23	127	111	109	8	0	2	7	1	1	2	2	0
	TGFβ1a	98	279	59	1374	891	1549	1330	270	177	659	888	211	66	122	125	7
	TGFβ1b									220	410	910	500				
T_H_2-signature	GATA3	>20590	472	113	10801	409	365	202	141	2669	98	13	24	16724	219	334	84
	STAT6	460	870	17	2354	2183	2650	2781	875	872	827	1274	827	1719	1313	1688	788
	IL-4/13A	1147	56	36	25	7	4	3	4	29	9	5	5	112	42	33	15
	IL-4/13B1	13	3	2	3	0	1	0	2	6	0	1	1	31	7	8	14
	IL-4/13B2				1	6	5	3	8	5	2	2	7	7	3	0	7
	IL-5fam?A	29	13	9	N.A.	N.A.	N.A.	N.A.	N.A.	N.A.	N.A.	N.A.	N.A.	N.A.	N.A.	N.A.	N.A.
	IL-5fam?B	6	29	12	N.A.	N.A.	N.A.	N.A.	N.A.	N.A.	N.A.	N.A.	N.A.	N.A.	N.A.	N.A.	N.A.

**Table:** **C.** Accession numbers of the SRA datasets and their number of total reads

	Elephant shark (*Callorhinchus milii*)				Golden mahseer (*Tor putitora*)			Northern pike (*Esox lucius*)					Rainbow trout (*Oncorhynchus mykiss*)				Atlantic salmon (*Salmo salar*)			
SRA dataset	SRX154852	SRX154856	SRX154860	SRX154855	SRX768559	SRX768561	SRX767362	SRX514237	SRX514263	SRX514240	SRX514270	SRX514238	ERX297522	ERX297511	ERX297523	ERX297509	SRX608399	SRX608574	SRX608599	SRX608567
Tissue	Gill	Kidney	Spleen	Intestine	Gill	Kidney	Spleen	Gill	Kidney	Head Kidney	Spleen	Intestine	Gill	Kidney	Spleen	Intestine	Gill	Kidney	Spleen	Intestine
No. of reads	71430454	118965654	83369382	147745918	41751362	34023336	51857480	58499888	60694314	61054936	61731442	60466858	39064840	32103778	41714660	40271788	59793962	61054936	60203316	59806348

## 6. Evidence Supporting the Existence of T_H_ Cells in Fish

In this and the following chapters with “fish” we refer to teleost fish if not mentioned otherwise. Readers should realize though that the relatively little information available for sharks suggests that in essence they have immune systems similar to those found in other jawed vertebrates [147,171]. It is of note, however, that despite the overall similarities, there are also some aspects of the fish immune system that importantly differ from the mammalian situation, such as the poikilothermic conditions and the absences of lymph nodes, of mammalian-type haematopoietic bone marrow, and of antibody class switching [147,171]. It is also of note that the general pattern of basic similarity does not involve all jawed fish species, like for example gadoid fish do not have an MHC class II presentation system [172]. In the below we only try to summarize the (teleost) fish consensus situation.

Formally, helper T cell function in fish probably cannot be considered proven. However, multiple lines of evidence indicate that fish T_H_ cells similar to their human counterparts do exist. Fish have B cells and macrophages, which like antigen presenting cells in mammals express MHC class II molecules [173,174,175,176], and fish have T cells which express somatically rearranged TCR-α and -β genes that are expressed and selected in a clonal manner [177,178]. Furthermore, teleost fish have CD4 molecules with a motif for signaling capacity (CD4-1 and CD4-2; [179,180,181,182]), as well as sets of CD3 and signaling pathway molecules necessary for T cell function [183,184]. Fish CD4 and MHC class II molecules are expressed at high levels in the thymus in a similar tissue organization as in mammals [174,180,185,186,187,188,189], suggesting that, like in mammals, the fish thymus generates T_H_ cells that have been negatively selected against self-antigens. Early thymectomy results in a decreased antibody response against “T-cell dependent antigens” [190], and anti-hapten B cell responses were found to be supported by carrier-specific aid of non-B cells in hapten-carrier immunized fish [191,192,193]. More recently, adaptive transfers of CD4-positive (CD4-1 positive) lymphocyte fractions of immunized ginbuna crucian carp to syngeneic non-immunized individuals were found to aid antigen-specific antibody and cell-mediated cytotoxic responses *in vivo* [194]. In a zebrafish model support of CD4-1 positive cells to an antigen-specific immune reaction was suggested by their enhanced cytokine gene expression profiles after zebrafish booster immunization [195]. Many teleost lymphocytes that express CD4-1 also express CD4-2 [182,194,195,196,197], but within the detection ranges of the applied assays it appears that teleost lymphocytes can also be single-positive for only CD4-1 or CD4-2 [182,196,197,198,199]. It is not sure yet which, if any, of the fish CD4 molecules can confer mammalian-type CD4 function. Definite evidence for T_H_ functions in fish may need experiments involving immunizations with different combinations of haptens and carriers (to reduce the chance of misleading results because of nonspecific immune stimulation by the antigens), purification of CD4-1^+^ and/or CD4-2^+^ lymphocytes, and the ability to manipulate MHC class II-presentation or -matching by antigen presenting cells; since recently those experiments appear to be possible, but they haven’t been done yet.

## 7. T_H_1-like Responses in Teleost Fish

In this paragraph and the following ones, we try to summarize principle similarities between data published for i1-i2 axis functions in fish and in mammals, and we will for example not discuss alternative functions encoded by fish-specific paralogous genes. We also try as much as possible to only reference those fish studies which allow straightforward conclusions in regard to polarization models, leaving out for example most studies that only investigated expression of i1 markers, or in which i1 markers were up-regulated together with markers of other types of immune responses as part of an inflammation reaction.

Important for the discussion of possible type 1 immunity in fish is that they appear to have perforin and granzyme containing *TCRαβ*^+^CD8α^+^ T cells that can kill virus-infected cells in a specific manner, as well as “natural killer” cells that display less specificity for their cellular targets (reviewed by [200,201]. Our definition of fish NK (-like) cells refers to perforin and granzyme containing non-B non-T lymphocytes with cell-killing ability, although their surface markers may be substantially different from those of mammalian NK cells [145]. Type 1 immunity pivots around the cytokine interferon γ (IFNγ), and T-bet is the most important transcription factor. Fish *T-bet* and *IFNG* can be expressed by CD4-1 positive cells [195,202]. The involvement of fish IFNγ in self-amplifying loops, as known in mammals, was suggested by the observation that in flounder systems recombinantly expressed IFNγ was able to induce *IFNG* expression in whole kidney leukocytes and in a permanent cell line [203].

In trout systems, the expression of *IFNG* by splenic leukocytes and head kidney cells could be stimulated by recombinant IL-15 [204] and recombinant IL-12 [205], respectively. It was speculated that the trout *IFNG* induced by IL-15 was expressed by NK and/or CD8^+^ T cells [204], because in mammals these cell types are known to be particularly stimulated by IL-15 and to be able to release considerable amounts of IFNγ [77,95,206,207]. In its turn, recombinant trout IFNγ was found to enhance the expression of the i1 cytokine genes *IL-15* and *IL-12A* in trout macrophage and fibroblast cell lines [204] and in Atlantic salmon head kidney cells [208], respectively. Because under similar conditions some other trout or Atlantic salmon cytokines were not upregulated [204,205,208], this suggests that also in fish the IFNγ, IL-15 and IL-12 molecules cooperate in type 1 immunity. Moreover, fish *IFNG* was found upregulated by viral dsRNA mimic polyI:C, under conditions in which some other cytokine genes were not upregulated [202]. In mammals poly(I:C) is known to specifically stimulate type 1 immunity [209]. In zebrafish spleen and head kidney poly(I:C) was found to enhance transcription of *T-bet*, while in the same experiments *STAT6* appeared to be downregulated [130]. Direct correlations between fish IFNγ addition and a transcription factor were reported for STAT1, with Zou *et al.* [120] reporting upregulation of *STAT1* expression in a trout macrophage cell line, and Yabu *et al.* [210] reporting the induction of human STAT1 phosphorylation in a human cell line transfected for expression of ginbuna crucian carp IFNγ receptor chain. STAT4 has hardly been investigated in fish. In summary, possible existence in fish of a mammalian-like T_H_1 transcriptional regulation network has not been clarified yet, but the available fragmentary data do agree with its existence.

A very important feature of type 1 immunity is that it suppresses the other types of immunity, and *vice versa*. Indirect indications for such suppression is that, similar as in mammals, expression of fish *IFNG* and *IL-4/13A* can be found up- or down-regulated in opposing manners [166,202]. Furthermore, under conditions in which recombinant trout IFNγ enhanced the expression of *IL-12A* in Atlantic salmon head kidney cells, the expression of some *IL-17A/F* family genes was slightly downregulated [208].

In conclusion, specific co-regulation of factors important for mammalian type 1 immunity suggests the existence of a basically similar i1-system in fish. However, because with the establishment of antibodies recognizing CD4 only recently it became more accessible to isolate (putative) fish helper T cells, available evidence supporting the existence of fish T_H_1 cells is still very limited. Definite evidence of T_H_1 cells will require the establishment of long-term proliferation assays for fish helper T cells and investigations of to which extent they can be polarized.

## 8. T_H_17-Like Responses in Teleost Fish

Important for the possibility of type 3 immunity is that fish do have heterophilic-neutrophilic granulocytes (neutrophils). In mammals, IL-17A and IL-17F molecules induce the release of abundant cytokines and chemokines from leukocytes and other cell types, which amongst others attract neutrophils [211]. Mammalian neutrophils form an important and first line of defense against infiltrating bacteria [212]. Like their mammalian counterparts, fish neutrophils have granules with the enzyme peroxidase, they can phagocytose bacteria, they rapidly and extensively migrate to bacterial infection sites, and have high bactericidal and respiratory burst capacities [213,214,215]. Furthermore, like mammalian neutrophils, after their stimulation fish neutrophils can release DNA in the form of neutrophil extracellular traps (NETs) [216].

Fish have several genes of the *IL-17A* + *IL-17F* family, which are called *IL-17A/F* followed by a number [217]. Fish *IL-17A/F* genes can be expressed by CD4-1 positive cells [195,202]. To our knowledge there is only one study that investigated fish IL-17A/F at the protein level, namely in grass carp by Du *et al.* [218]. Du *et al.* found that recombinant grass carp IL-17A/F stimulates expression of the genes for the cytokines IL-1β, IL-6 and TNF-α and the chemokine CXCL-8 (alias IL-8) in head kidney cells. Other studies have shown that in fish CXCL-8, like in humans, can recruit neutrophils [219,220]. Hence, it is likely that fish IL-17A/F can induce neutrophil recruitment, although there is no direct evidence at the protein level for that in fish yet.

However, a study by Ribeiro *et al.* [221] provides supportive evidence at the gene expression level that also in fish IL-17A/F probably is involved in neutrophil recruitment. Ribeiro *et al.* [221] compared the infections in common carp of the related protozoan parasites *Trypanoplasma borreli* and *Trypanoplasma carassii*, which cause quite different patterns of disease development. At a time-point of infection in which *T. borreli* induced higher levels of *IFNγ* expression than induced by *T. carassii*, only *T. carassii* induced enhanced *IL-17A/F* expression, which was accompanied by a marked neutrophil infiltration into the spleen of only *T. carassii*-infected fish. Ribeiro *et al.*, furthermore showed that factors derived from these parasites could *ex vivo* stimulate expression of *IL-23A* in head kidney leukocytes from parasite-infected fish, and that these same factors could efficiently interact with carp toll-like receptor 2 (TLR2) molecules expressed on a human cell line [221]. Hence, for their *T. carassii*-infected carp model, the authors postulated a “T_H_17-like immune response” model involving the stimulation cascade *T. carassii* -TLR2-IL23-T_H_17-IL17A/F-neutrophil. We are not aware of other studies in fish that suggest a correlation between IL-23 and IL-17A/F expression.

In Atlantic salmon head kidney cells, under conditions in which recombinant trout IL-1β induced large increases in expression of the pro-inflammatory cytokine genes *IL-1B* and *TNFA*, the expression of *IL-17A/F* genes remained unchanged [208]. In the same study recombinant trout IFNγ stimulated the expression of i1-signature gene *IL-12A*, while slightly reducing the expression of *IL-17A/F* genes [208]. In contrast, in this type of experiments, recombinant trout IL-21 was found to enhance expression of *IL-17A/F* genes [208], as well as of *IL-22* [222], suggesting specific involvement of IL-21 in T_H_17 polarization as known in mammals.

An antibacterial function of fish IL-22 was indicated by *IL-22* upregulation induced in several fish species through bacterial agents, by the stimulation through recombinant IL-22 of antimicrobial peptide synthesis in trout and mullet, by the protection of mullet and turbot against bacterial challenge after injection with recombinant IL-22, and by a decreased resistance against bacterial challenge after *IL-22* knockdown in zebrafish embryos [170,223,224,225,226]. In zebrafish *IL-22* can be expressed by CD4-1 positive cells [195], and at the tissue level there are a few studies suggesting some correlation between fish *IL-22* and *IL-17A/F* expression ([170]; Table 1).

In our opinion, there are no convincing reports in fish yet linking expression of T_H_17 signature cytokine genes to *RORC* expression.

In summary, from the available evidence it seems likely that also in fish the molecules IL-17A/F, IL-21 and IL-22 (and possibly IL-23) can be orchestrated in an anti-bacterial defense response that involves recruitment of neutrophils by IL-17A/F-induced chemokine expression. But it is unclear whether this response also involves RORγ(t) and/or TGF-β molecules, and/or the T_H_17-like polarization of helper T cells. We expect that research of fish T_H_ cell polarizations will first concentrate on the possibility of i1-i2 axis end polarizations, T_H_1 and T_H_2, because in mammals these are so pronounced and well-defined, before possible T_H_17 polarization will be convincingly addressed.

## 9. T_reg_-like Responses in Teleost Fish

Studies have shown that if fish are fed or otherwise treated with antigen preparations that lack PAMPS or DAMPS, their immune system can develop some level of tolerance against these antigens (e.g., [227,228]). In mammals immune tolerance is importantly mediated by natural or induced T_reg_ cells, for which FOXP3 is a master transcription factor and which are immunosuppressive by a variety of means, including the release of the cytokines IL-10 and TGF-β [229,230].

Fish *FOXP3*, *TGFB1* and *IL-10* genes can be expressed by CD4-1 positive cells [202]. Wen *et al.* [231] showed that an unknown percentage of the Tetraodon lymphocyte population positive for CD4-2 and IL-15Rα molecules also expressed *TCRα* and *FOXP3*. Fish IL-15Rα is a receptor chain that can bind IL-2 as well as IL-15 and, although it looks like mammalian IL-15Rα, it corresponds to the evolutionary precursor form of both mammalian IL-2Rα and IL-15Rα [161,231]. Under non-stimulated conditions, in mammals, T_reg_ cells are the cell type expressing the highest amount of IL-2Rα and are the most sensitive to IL-2 [232,233]. The fact that Wen *et al.* [231] didn’t detect *FOXP3* expression in the CD4-2^+^IL-15Rα^−^ cells suggests that also in fish IL-2 may have an important function in a negative feedback loop of immune reactions through activation of T_reg_ (-like) cells [161,234]. Wen *et al.* [231] reported immunosuppressive functions of Tetraodon CD4-2^+^IL-15Rα^+^ cells observed during *in vitro* and *in vivo* experiments. The *in vitro* experiments showed that if CD4-2^+^IL-15Rα^+^ cells were depleted from Tetraodon spleen and head kidney leukocytes, the remaining cell population became more effective in executing non-specific cell-mediated cytotoxicity and in inducing mixed lymphocyte reactions in the respective assays [231]. Repeated *in vivo* treatment of Tetraodon with rabbit antibodies binding to Tetraodon IL-15Rα resulted in bowel inflammation [231], which the authors interpreted as deriving from a depletion of T_reg_ cells, a model for which they provided some evidence but which may need more investigation.

Quintana *et al.* [235] found that zebrafish *FOXP3* (*FOXP3a*) was expressed by lymphocytes, and that in zebrafish embryos overexpression or knockdown of *FOXP3* resulted in decreased *vs.* increased *IL-17A/F* expression, respectively [235]; this perfectly agrees with FOXP3 involvement in immunosuppressive functions as known in mammals. Quintana *et al.* [235] also showed that zebrafish FOXP3 retained its capacity to induce T_reg_-like features upon expression in mammalian cells, because murine T cells transfected with zebrafish *FOXP3* were found to suppress activation of other murine T cells.

In goldfish monocytes recombinant IL-10 suppressed the immune response induced by bacterial agents as indicated by reduced increase of expression of genes for TNF-α, IL-10, CXCL-8, IFNγ and several NADPH oxidase subunits, as well as by a reduced increase in production of reactive oxygen intermediates [236]. Similar results were found for carp, where recombinant IL-10 was shown to reduce the expression increase induced by LPS in neutrophils and/or macrophages of genes for TNF-α, IL-1β, IL-6, IL-12A, MHC class I, and MHC class II molecules [237]. Piazzon *et al.* [237] also found that carp IL-10 could induce STAT3 phosphorylation, implying similar signaling cascades as in mammals. It is of note that Piazzon *et al.* [237] also revealed that carp IL-10 does not only have immunosuppressive functions, but, like known for multifunctional mammalian IL-10, also has some stimulatory and modifying effects on the immune system, like the stimulation of proliferation, differentiation, and antibody secretion by IgM^+^ B cells.

In regard to TGF-β, it is difficult to distinguish clear regulation patterns from the number of studies that investigated fish *TGFB1* expression. We therefore decided not to try to summarize those studies. However, it is important that an immunosuppressive function was found for recombinant goldfish TGF-β, as it was shown to down-regulate the nitric oxide response of TNF- α-activated macrophages [238].

In summary, FOXP3 in fish has been associated with immunosuppressive functions, and, at least in Tetraodon, FOXP3 is expressed by CD4 positive T cells that constitutively express high levels of IL-2 receptor chain. This suggests the existence of a T_reg_ system in fish similar to that found in mammals. Both fish IL-10 and TGF-β were found to have immunosuppressive functions. It will need further investigation whether in fish, as known in mammals, IL-10 and TGF-β are associated with FOXP3 positive cells.

## 10. T_H_2-like Responses in Teleost Fish

For the possibility of raising T_H_2-like responses, it is relevant that fish do have granulocytes other than neutrophils which have anti-parasite functions. Whether (some of) these cells can be called eosinophils, basophils and/or mast cells depends on the chosen definition and on the fish species. Our use of the terms eosinophils and mast cells in the text below is based on the definitions in the indicated references. Similar to mammalian eosinophils, fish eosinophils express transcription factor GATA-2, and can migrate to sites of parasite infection and release their peroxidase containing granules upon stimulation by parasite agents [239,240]. Fish mast cells, which are abundant in the gill and the intestine, can also accumulate and degranulate at the site of parasitic infection [239,240,241,242,243]. Similar but not identical to mammals, the granules of fish mast cells contain phosphatases, peroxidase, proteolytic enzymes, arylsulfatase, 5'-nucleotidase, lysozyme, antimicrobial peptides, and, depending on the species, they can contain serotonin or histamine [241,242,243,244].

Fish *GATA-3*, *IL-4/13-A* and *-B* genes can be expressed by CD4-1 positive cells [195,199,202]. Chettri *et al.*, found that if rainbow trout skin was infected with the parasitic flagellate *Ichthyobodo necator*, locally there was substantial increase in *GATA-3* but not of *FOXP3* or *T-bet* expression, concomitant with a substantial decrease in the number of CD8α^+^ cells and a substantial increase in IgM^+^ B cells [245]. This might represent a T_H_2 response, as suggested by the authors, although all investigated cytokine genes including *IFNG* were found upregulated. More convincing of a T_H_2 response are conditions in which *IL-4/13A* expression increases while *IFNG* expression decreases, as could be found in experiments analyzing (cells of) trout gill [166,246]. Very interestingly, because it suggests that fish T_H_2 responses are involved in anti-parasite immunity, the infection of salmon with the parasite *Paramoeba perurans* enhanced the expression in infected gill of T_H_2 signature genes *IL-4/13A* and *IL-4/13B* while the expression of signature genes for T_H_1, T_H_17 and T_reg_ like *IFNG*, *IL-17A/F*, *TGFB1* and *IL-10* were downregulated [246]. An opposite regulation of T_H_1 and T_H_2 signature genes was also found by Zhu *et al.* [247] who showed that injection into zebrafish of recombinant IL-4/13A resulted in an increase in expression of *GATA-3* and *STAT6* in the spleen, while concomitantly the expressions of *T-bet* and *IFNG* were decreased; curiously, the authors did not check the effect on *IL-4/13-A* or *-B* expression. Regarding opposite regulations an important observation is also that injection into zebrafish of recombinant zebrafish “IL-4” (probably IL-4/13A) induced expression of CD209 in blood leukocytes, while addition of LPS to the IL-4 preparation caused a reduction in the CD209 increase [248]. For carp we established a clonal (semi-) permanent *CD4-1*^+^*TCRα*β^+^ T cell line that expresses readily detectable amounts of *GATA-3* but not of *T-bet*, thus has a T_H_2-like profile in regard to its transcription factors [199]. In agreement with T_H_2-like polarization, this cell line lost the ability to increase its *IFNG* expression after suitable stimulation while it retained an ability for upregulation of *IL-4/13B*; curiously, we were unable to increase *IL-4/13A* expression in this cell line [199]. It remains to be determined whether the carp cell-line phenomenon represents an artefact introduced by prolonged *in vitro* culture, or that not all fish T_H_2-like cells can make significant amounts of IL-4/13A.

An important finding by Zhu *et al.* [247] analyzing recombinant zebrafish IL-4/13A was that the cytokine can bind to receptor chain IL-4Rα. The same study also provided evidence that zebrafish IgM^+^ B cells specifically express IL-4/13Rα and that they can be stimulated by recombinant zebrafish IL-4/13A, although the extent of the specificity was not investigated [247]. In mammals the stimulation of B cell activity is not restricted to i2-skewed conditions, but mammalian IL-4 is one of the molecules that can efficiently stimulate B cell proliferation and the molecule was originally named “B cell growth factor” [249]. Thus although the B cell stimulation by zebrafish IL-4/13A does not provide direct evidence of a T_H_2 function, it does provide additional evidence that IL-4/13 functions in fish are similar to those of their mammalian counterparts.

In summary, there is accumulating evidence that in fish the expressions of *GATA-3* and *IL-4/13A* are correlated, and that their expression suppresses the expression of T_H_1 signature genes. Although the regulation mechanisms in fish have not been elucidated yet, it seems likely that fish T cells can polarize into a T_H_2 phenotype by mechanisms similar to those in mammals. Fish IL-4/13A has been shown to stimulate B cells, but it still needs to be investigated whether fish IL-4/13A can stimulate typical i2 functions such as anti-parasite activities of eosinophils and mast cells.

## 11. M1-like *vs.* M2-Like Macrophage Polarizations in Fish

Like mammals, fish have macrophages with potent phagocytic and bactericidal abilities that make use of reactive oxygen and nitrogen intermediates (reviewed by [250]). Also, like in mammals, zebrafish macrophages are found in healing wounds [251,252,253] and are important for normal tissue regeneration [254]. The involvement in both M1-like and M2-like functions opens the possibility of differential polarization towards those functions. As listed below, there is some evidence that fish macrophages can polarize towards M1- or M2-like phenotypes through similar pathways as known in mammals. The best review on that has probably been published by Forlenza *et al.* [255], who importantly realized that also in studies on fish M2 macrophages it is necessary to conceptually distinguish between M2a (alias “alternatively activated”) and M2c (alias “deactivated”, or, as Forlenza *et al.*, not unreasonably call it, “regulatory”) polarizations. For lack of solid polarization data, Forlenza *et al.* [255] developed a working definition, based on how fish macrophages were stimulated, to divide them into four “polarization states”, akin to as how this has been accepted by some researchers studying mammalian macrophages (e.g., [92]) as a simplification of the classification system by Mantovani *et al.* [60]. Forlenza *et al.* [255] defined the above mentioned M2a and M2c polarizations as those deriving from stimulation with IL-4/13 and from stimulation with microbial agent + IL-10, respectively. At the far i1-end of the polarization spectrum, Forlenza *et al.* [255] conventionally considered macrophages stimulated by both microbial agents plus IFNγ as “classically activated” (M1). The definition by Forlenza *et al.*, which may not hold in the long term, but which is practically convenient and seems to define macrophages only somewhat shifted towards the i1-end (not unlike the mammalian M2b macrophages; [60,92]), concerns “innate activated macrophages” (“iaM”) which are stimulated with only microbial agents and not with IFNγ. Similar to mammalian M1 and M2b, fish M1 and iaM express iNOS (reviewed by [255]).

Although not in all cases studied as one among more possible polarizations, there is evidence that M1 phenotypes can be induced by similar agents as in mammals. In synergy with LPS, carp IFNγ was found to stimulate carp macrophages into expressing higher levels of *IL-12A* and *TNFA* [256]. Furthermore, recombinant rainbow trout IFNγ plus some LPS enhanced respiratory burst activity of rainbow trout macrophages [120], and recombinant goldfish IFNγ, said to be without LPS contamination, primed goldfish monocytes/macrophages for enhanced respiratory burst, phagocytic and nitric oxide responses, while it also stimulated their expression of genes for TNF-α, IL-1β, IL-12α, IL-12β and iNOS [257,258]. Likewise in agreement with M1 differentiation as known in mammals, recombinant goldfish TNF-α could also prime goldfish monocytes/macrophages for enhanced respiratory burst, phagocytic and nitric oxide responses [257,259].

There appears to be little direct evidence for the existence of M2c (“M_reg_”) polarization of fish macrophages, but recombinant IL-10 or TGF-β were found able to reduce M1-type macrophage activations [236,238].

In carp differential polarizations of macrophages using LPS *vs.* cAMP stimulation have been shown [260]: while neither LPS nor cAMP stimulated *IL-10* expression and both stimulated *IL-1B* expression, only LPS stimulated *NOS2* (the gene for iNOS) and only cAMP stimulated *ARG2* (the gene for arginase 2) expression. However, although in mammals cAMP is one of the factors contributing to an i2 environment [21], we are somewhat hesitant to accept the isolated addition of cAMP as an inducer of a natural M2 (-like) polarization. We would also like more research to be done before concluding that in fish arginase 2 and not arginase 1 is a major marker for M2 differentiation (for a discussion on fish arginase genes see [260] and [255]).

One of the tasks of macrophages is the removal of cell debris, and in mammals apoptotic bodies are known to stimulate an M2 phenotype [261,262]. Zymosan is a glucan-rich particle prepared of the surface of fungi, which in mammals can induce pro-inflammatory responses and in synergy with other factors can stimulate M1 polarization [263,264]. *In vitro* analysis of goldfish macrophages showed that their respiratory burst activity induced by PMA treatment was enhanced by incubation with zymosan and reduced by incubation with apoptotic bodies [265]. Injection of zymosan or apoptotic bodies in the goldfish peritoneal cavity, followed by isolation of myeloid cells and analysis of their ability to generate respiratory burst responses, showed that the *in vivo* treatment had a similar effect on priming for respiratory burst activity as the above described *in vitro* treatments [265].

An interesting study was recently published by Nguyen-Chi *et al.* [253], who used a double fluorescent labeling system for zebrafish macrophages (using the *MPEG1* promoter) and TNF-α expression (using the *TNFA* promoter). They found at the population level that *TNFA* expression by zebrafish macrophages positively correlated with their expression of *IL-1B* and *IL-6*, and negatively correlated with their expression of *TGFB1* and *CXCR4*. In mammals both *TGFB1* and *CXCR4* have been used as markers for M2 macrophages [266], and Nguyen-Chi designated the *TNFA*-high macrophages as “M1-like” and the *TNFA*-low macrophages as “M2-like”. Nguyen-Chi *et al.* [253] also found that in wounded fin of zebrafish larvae the *TNFA*-high macrophages tended to display a flattened and lobulated morphology, whereas the *TNFA*-low macrophages tended to be elongated and dendritic. Other than creating a wound by fin amputation, Nguyen-Chi *et al.* [253] also inoculated zebrafish larvae with *E. coli*. Based on the abundances of the different macrophages at the relative sites in these experiments, Nguyen-Chi *et al.* [253] concluded that zebrafish M1-like macrophages are important in anti-bacterial combat and initial stages of wound healing and that M2-like macrophages are important in especially the later stages of wound healing. Nguyen-Chi *et al.* [253] furthermore showed that in later stages of wound healing the *TNFA*-positive (M1-like) macrophages changed towards a phenotype which they call intermediate to M1 and M2 and which shows high *TGFB1* expression besides lowered *TNFA* expression.

In summary, fish macrophages can be stimulated towards several phenotypes, and at least the M1 phenotype seems to be defined by similar pathways and characteristics as in mammals. The fish macrophage non-M1 polarizations are not well characterized and appear to be predominantly defined by reduced M1 features and maybe by the upregulation of *ARG2*. Hopefully this gap in knowledge on possible non-M1 polarizations can be closed by future inclusion of recombinant fish IL-4/13 cytokines in the macrophage polarization assays.

## 12. I2-Skewed Tissue Milieus in Healthy Mammals and Fish

### For fish this paragraph has an overlap with paragraph 5, but we nevertheless like to dedicate a special paragraph to the comparison between fish and mammals.

Previously we reported that trout and salmon gill and skin appear to have i2-skewed milieus since we observed rather consistent high ratios of *IL-4/13A* plus *GATA3 vs. IFNG* expression [166]. We found similar high ratios for the thymus of trout, salmon and mouse, but it is discussable whether a primary immune organ with its unique immune functions can be classified as “i2-skewed” [166]; on the other hand, it was found in mammals that recent thymic emigrants have a bias towards T_H_2 polarization [267], thus at least in some sense the thymus can be seen as “i2-skewed”. High levels of *IL-4/13A* and/or *GATA3* in fish gill were also found by others [121,167,168], and we are glad that in the present paper we could additionally confirm these findings by SRA dataset analysis for several teleost fish and also for elephant shark (see paragraph 5). We speculate, as before [166], that i2-skewage of the fish gill helps to protect it against parasites, but also against possible i1- or i3- type inflammation that might harm this delicate tissue. There are some data indeed that indicate that it is hard to induce an i1-response in fish gill [166,268], but other studies suggest that it is possible to induce i1- or i3- responses in this tissue (e.g., [269]); more research will be needed to clarify the degree of i2-skewage of fish gills.

Our idea that a sensitive fish tissue like gills may be i2-skewed for its protection from other types of immune reactions actually derives from similar claims made in mammals for the immunity of pregnancies [270,271,272,273] and neonates [33,34,274]. Whether these claims for mammals are actually true, however, has, at least in a general sense, been disputed [275], and precise locations, conditions and measured parameters should probably be acknowledged.

We did some preliminary analysis of tissue-specific transcriptomes for mammals available in public databases to assess the expression levels of i1-i2 axis marker genes, similar to the method followed to make Table 1, but could not distinguish notable expression patterns (data not shown). However, in mammals pronounced tissue-specific distinctions were observed by others who investigated the i1-i2 axis positioning of individual cell types. It is intriguing, for example, that IL-13 secreting ILC2 cells can readily be found in the mouse lung and skin, while these tissues have hardly any ILC3 cells [276]; in a way this is reminiscent of the i2-skewage observed in fish skin and respiratory tissue (gill). For the mouse healthy intestine an opposite ratio is found, with abundant ILC3 cells and relatively few ILC2 cells [276]; this agrees well with the facts that also in fish this mucosal tissue is not i2-skewed, and that in some fish including elephant shark the intestine may be i3-skewed.

Overall, however, it is surprising to us how little work there seems to have been done in mammals to analyze non-diseased tissues for their immune biases along the i1-i2 axis. It seems to us that this should be important information when considering how and where to administer vaccines or therapeutic agents.

## 13. I2-Skewed Tissue Milieus of Tumors in Mammals and Fish

The only mammalian “tissues” for which i2-skewage has been intensively studied and generally accepted as proven are a variety of tumors. The i2-skewage protects the tumors from eradication by type 1 immunity, and immunotherapy shifting the tumor immune milieu towards type 1 immunity has been shown helpful in fighting the cancer. The champion results so far are obtained by antibodies that can block the immunosuppressive functions of the molecules PD-1 and CTLA-4, but many other methods to induce a shift towards i1-immunity are being tried [116,117,277].

It is important to realize that in tumor studies the term “type 2 immunity” tends to be used for a combination of immunosuppressive (“T_reg_-type”) and i2 inflammation (“T_H_2-type”) conditions, and that among these the immunosuppressive conditions probably are more pronounced. The difficulty with changing this immune milieu by therapy is that the cancer cells and the i2-skewed immune cells reciprocally attract/support each other, so that effects of therapeutic i1-stimuli tend to be undone once the stimuli fade out after administration.

An example of a situation in which tumor cells and infiltrating immune cells support each other is found in human and rodent pancreatic ductal adenocarcinoma (PDA). In rodent models, expression of mutant *KRAS* oncogene in pancreatic ductal epithelial cells is sufficient to induce their cancerous proliferation and their expression of factors like STAT3, NF-κB and IL-6 that form part of a (self-) amplifying loop [278,279,280]. These tumor cells release abundant amounts of chemokine CCL2 which attracts monocytes that within the tumor stroma develop into M2 macrophages [281,282]. The tumor is also invaded by abundant lymphocytes, including many T_H_2 and T_reg_ cells, while few are T_H_1 or CD8^+^ T cells [282,283,284]. Despite abundant infiltration with immune cells, in a rat PDA model the expressions of the i1-markers *CD8B*, *IL-15* and *granzyme-C* were found to be 2-, 5- and 5-fold lower than in healthy rat pancreas [285]. Meanwhile, in these rat PDA samples, the expressions of i2 markers *TGFB1* and *IL-33* were found to be 14- and 18-fold higher compared to healthy rat pancreas [285]. Experiments in a mouse PDA model have shown that the cancer cells and M2 macrophages reciprocally support and stimulate each other [286].

Unfortunately in fish the immunology of tumor microenvironments has hardly been studied yet, but there is a nice study by Yan *et al.* [287] which indicate that also in fish the progress of tumors can be enhanced by i2 conditions. Yan *et al.* [287] found that when they induced mutant KRAS expression in zebrafish hepatocytes, this resulted in rapid recruitment of (fluorescently labeled) neutrophils to the liver area and in hepatocarcinogenesis. The experimental results of independent knockdowns of *GCSFR* and *IRF8* let the authors conclude that the infiltrating neutrophils enhanced carcinoma growth. By using specific stimulators or inhibitors of neutrophils, they concluded that the neutrophils stimulated proliferation of the mutant KRAS expressing hepatocytes, while reducing their apoptosis. They found that the nucleus morphology of the infiltrating zebrafish neutrophils resembled that of tumor associated neutrophils in mammals and that they displayed a modified cytokine gene expression profile, which they speculated, based on high *IL-1B* expression, to support angiogenesis. Yan and co-workers [287] also found that the mutant KRAS expressing hepatocytes expressed increased amounts of *TGFB1* (*TGFB1a*), and that blocking of TGF-β pathways reduced both the number of neutrophils and carcinoma growth, and changed the cytokine gene expression pattern of the tumor-associated neutrophils. While some important questions still remain to be answered in the zebrafish model provided by Yan *et al.* [287], their study allows the important conclusion that also in fish tumor development can be supported by TGF-β expression and by infiltration of immune cells that adapt their phenotypes under influence of TGF-β.

In summary, considering the enormous medical importance of the immune milieus of tumors, it is surprising how little this matter has been studied in fish. However, the first results appear to confirm that like in mammals, tumor growth in fish can be stimulated by i2 cytokines and by interaction with leukocytes. We expect that soon many more studies on the immune milieus of tumors in fish will be performed.

## 14. Conclusions and Future Prospects

In the present study we have tried to summarize the i1-i2 axis and its effect on leukocyte polarizations in mammals in a model (Figure 1) that reflects our interpretation of literature consensus. Although not unique, the difference from most existing models is the stressing of the continuity between the different conditions and their associated leukocyte polarizations, in combination with the placement of the i3 and “T_reg_-type” conditions/polarizations between the i1 and “T_H_2-type” conditions/polarizations. We feel we need this type of model to be able to compare between polarizations of different cell types and beyond species borders, and that at the very least our model is a good starting point for discussion. The Figure 1 model automatically leads to the question whether it wouldn’t be better if the term “type 2 immunity” would be split up into T_reg_-type (“regulatory immunity” alias “i-reg”?) and a narrower definition of type 2 immunity (T_H_2-type). However, such change of nomenclature would need a thorough discussion on how entangled T_reg_-type and T_H_2-type conditions are, and whether the change of nomenclature would reduce or increase the confusion.

The strongest evidence that the fish immune system obliges to similar i1-i2 axis principles as known in mammals comes from the remarkable conservation of many of the most important gene loci. But beyond that, recent years have also seen an accumulation of functional data that support that fish leukocytes respond to i1-i2 axis factors in a similar way as they do in mammals. Although these functional data are still fragmentary, when considered together they are rather convincing. In future research of the i1-i2 axis regulation of the fish immune system it hopefully will become more commonplace to simultaneously investigate multiple possible polarizations, so that they can be compared directly. Furthermore, it should be attempted to maintain long-term T cell cultures under polarizing conditions, followed by analysis of epigenetic modifications and of the stability of the polarized phenotype. In the short term we expect the biggest breakthroughs from the research of fish macrophage polarizations, because a lot of good work has been done already. For research of the tumor microenvironment (transparent) fish appear to be a great model, and like in mammals, it can be expected that a lot of i1-i2 research in fish will be dedicated to tumor tissues.

Besides the general discussion on the evolution of the i1-i2 axis, very interesting points of the present study are the findings that throughout bony as well as cartilaginous fish the gills appear to be i2-skewed, and that with analysis of the spotted gar genome sequence a canonical type T_H_2 locus was found in bony fish that harbors *RAD50* as well as genes of both *IL-4/13* and *IL-3/IL-5/GM-CSF* families. Future functional research should help to clarify the identity of the IL-5fam? sequences in teleost fish.

## Supplementary Files

Supplementary File 1
