# Supplementary material for: Along the Axis between Type 1 and Type 2 Immunity; Principles Conserved in Evolution from Fish to Mammals
Source: Biology (Basel). 2015 Nov 17;4(4):814–59. doi: 10.3390/biology4040814 (PMC4690019; doi:10.3390/biology4040814)
Supplement: Supplementary File 1 [file biology-04-00814-s001.zip › biology-4040814-supplementary 1.pdf]

Supplementary Materials

Supplementary File 1

Many gene loci involved in the i1-i2 axis are conserved between mammals and fish. Subsequently the following gene loci are shown: Figure S1: *STAT1* and *STAT4*; S2: *STAT3* and *STAT5*; S3: *STAT6*; S4: *TBX21* (T-bet); S5: *RORA*; S6: *RORC*; S7: *FOXP3*; S8: *GATA3*; S9: *NFKB1*; S10: *RELA*; S11; *SMAD2*; S12: *SMAD3*; S13: *TNFA*; S14: *IL-1B*; S15: *IL-17A/F*; S16: *IL-12A*; S17: *IL-23A*; S18: *IL-6*; S19: *IL-15*; S20: *IL-2* and *IL-21*; S21: *IL-10*; S22: *TGFB1*; S23: *NOS2* (iNOS); S24: *ARG1*. See main text Figure 2 for explanation of symbols and for references to sequence databases. Identical colors of pentagons in the same figure refer to gene homologies, and white color of pentagons refers to absence of homologues within the figure. Depicted orientations of human genomic fragments follow the Ensembl database, and depicted orientations of genomic fragments of other species are arranged to best show syntenies. If in a species there are duplicated genomic regions, these are not shown if that region doesn't contain reported bona fide copies of the gene of interest. For gene nomenclature in most cases we simply followed annotations made by databases or by other researchers. We listed GenBank accession numbers of automated predictions of spotted gar molecules to help the readers with sequence identification, but readers should be aware that these may or may not represent proper/consensus full-length sequences. Some of our sequence predictions only concern partial molecules. Article references and comments are provided below each synteny figure; our referencing does not attempt to be exhaustive.

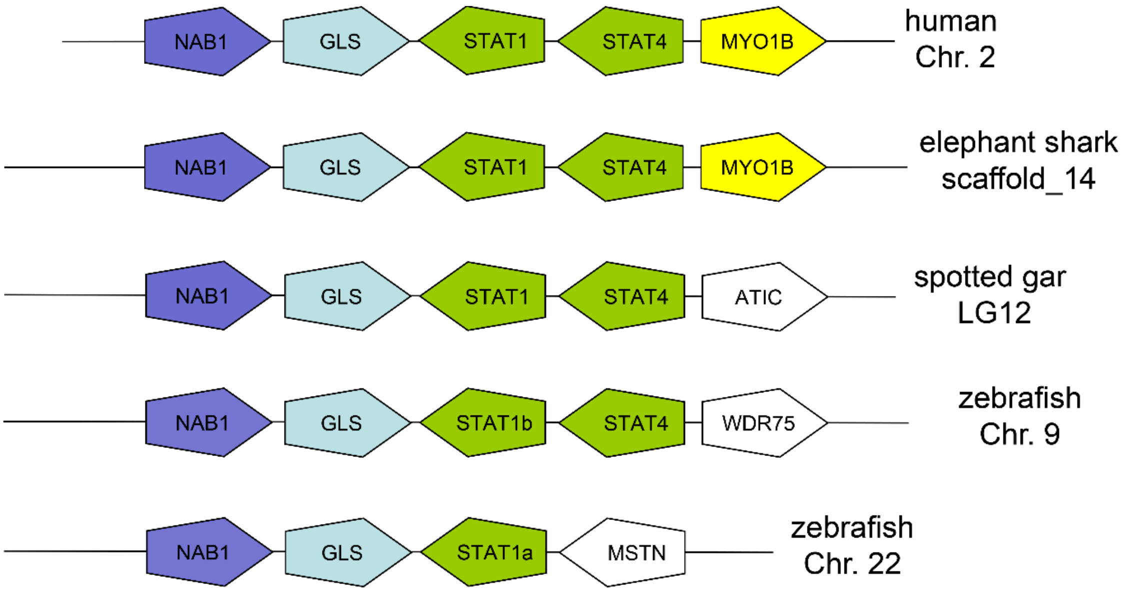

Figure S1. *STAT1* and *STAT4*. The elephant shark *STAT1* and *STAT4* genes were identified by Venkatesh *et al.* [1]. For spotted gar *STAT1* and *STAT4* see GenBank accessions XP_006636727 and XP_006636800, respectively. The duplicated zebrafish *STAT1* genes were discussed by Song *et al.* [2].

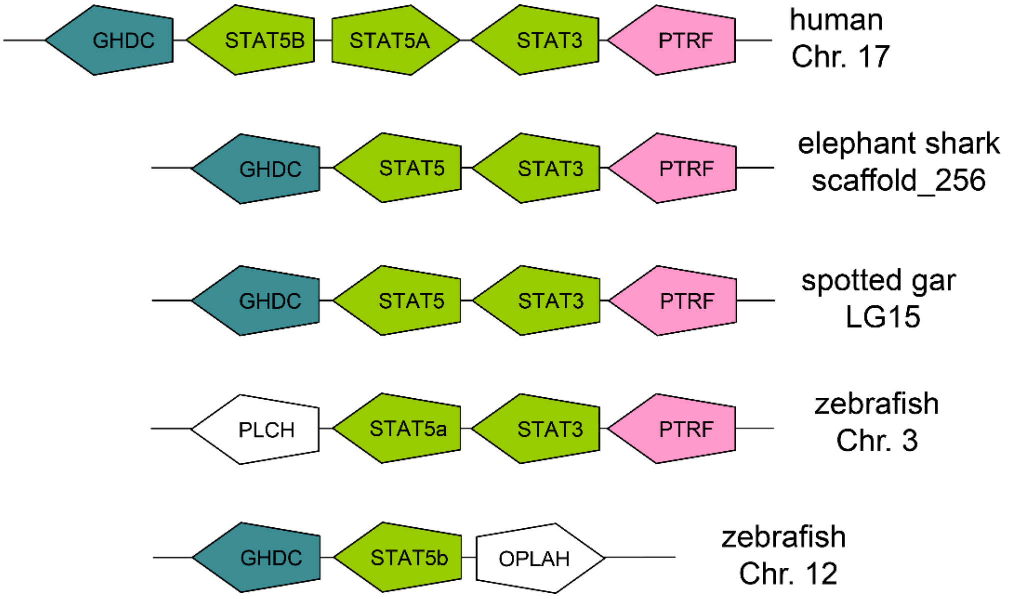

Figure S2. *STAT3* and *STAT5*. The elephant shark *STAT3* gene was identified by Venkatesh *et al*. [1] and for *STAT5* see GenBank accession XP_007907688. For spotted gar *STAT3* and *STAT5* see GenBank accessions XP_006638199 and XP_006638200, respectively. Zebrafish *STAT3* and *STAT5* genes have been discussed by Lewis and Ward [3] and Gorissen *et al*. [4].

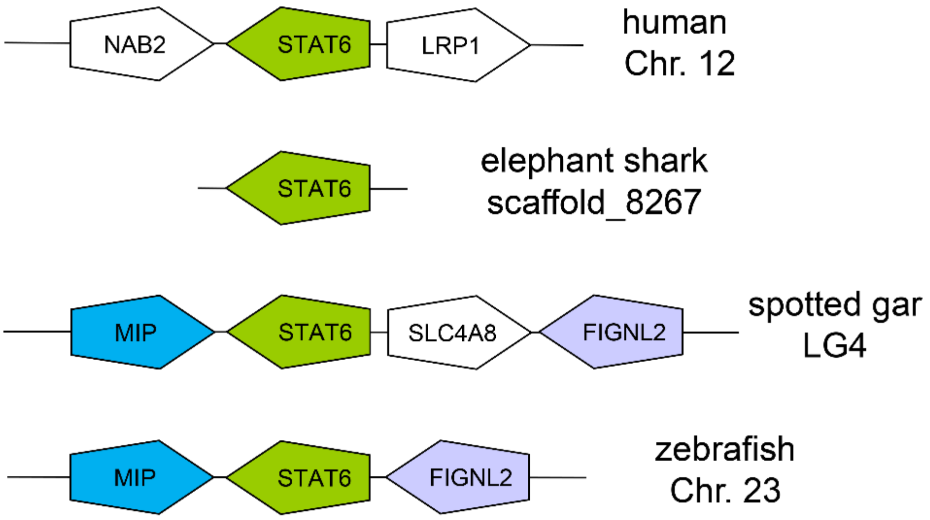

Figure S3. *STAT6*. Elephant shark *STAT6* gene was identified by Venkatesh *et al*. [1]; the respective elephant shark genomic sequence scaffold, scaffold_8267, is too short for gene synteny analysis. For spotted gar *STAT6* see GenBank accession XP_006629463. Zebrafish *STAT6* was discussed by Mitra *et al*. [5]. More diverged syntenies not shown in the figure are: Spotted gar *NAB2* and *LRP1* map to LG4 within 4 Mb and 2 Mb from *STAT6*, respectively. Zebrafish *LRP1* maps to Chr. 23 within 2 Mb from *STAT6*. Human *MIP*, *SLC4A8* and *FIGNL2* map to Chr. 12 within 1 Mb, 6 Mb and 6 Mb, respectively, from *STAT6*.

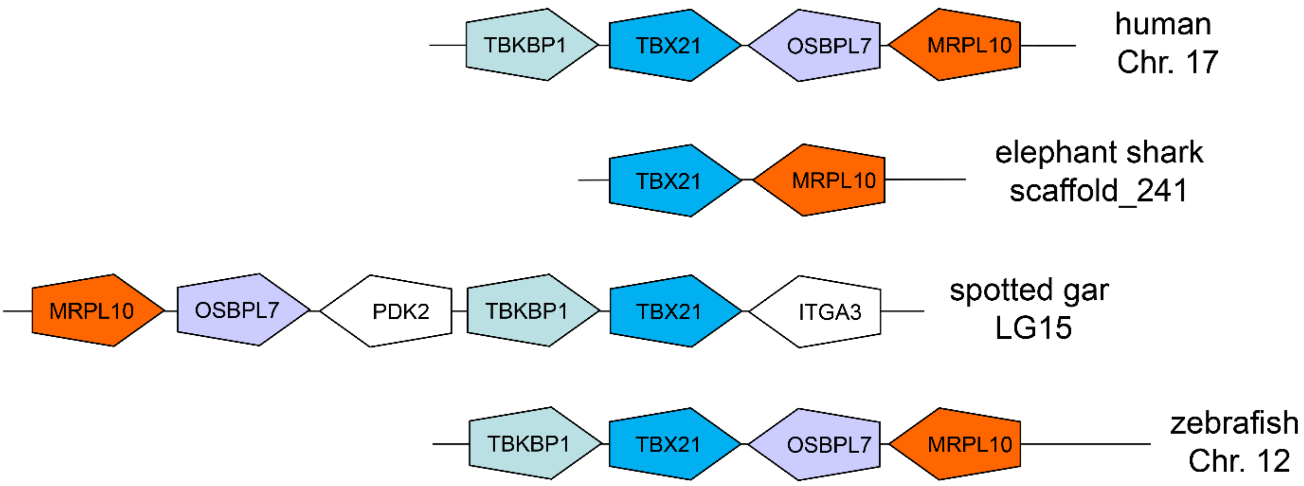

Figure S4. *TBX21* (T-bet). The elephant shark *TBX21* gene was identified by Venkatesh *et al.* [1]. The *TBX21* gene maps to the end of elephant shark scaffold_241, thus the upstream genes could not be determined for this species. For spotted gar *TBX21* see GenBank accession XP_006638408. Zebrafish *TBX21* has been discussed by Takizawa *et al.* [6] and Mitra *et al.* [5].

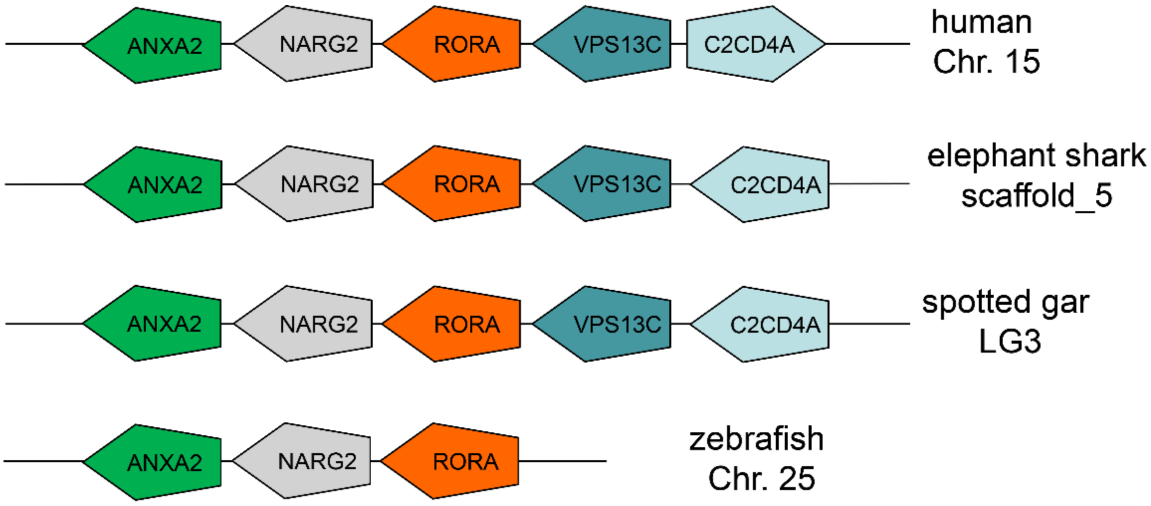

Figure S5. *RORA* (RORα). The elephant shark *RORA* gene was identified by Venkatesh *et al.* [1]. For spotted gar *RORA* see GenBank accession XP_006628975. Zebrafish *RORA* has been discussed by Flores *et al.* [7]. A more diverged synteny not shown in the figure is: zebrafish *C2CD4A* maps to Chr. 25 at 1 Mb from *RORA*.

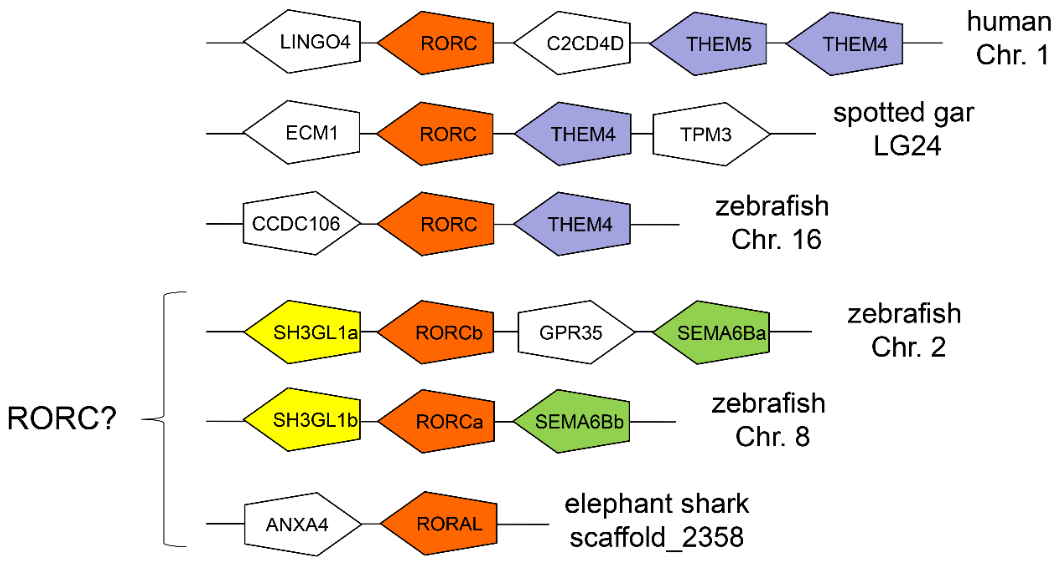

Figure S6. *RORC* (RORγ and RORγt). The elephant shark *RORAL* gene at scaffold_2358 was identified by the Ensembl database as an "*RORA*" gene (SINCAMG00000011495); we added "-like" (L) to the name. The elephant shark scaffold_2358 sequence is very short and does not provide information on neighboring genes other than *ANXA4*; *ANXA4* is not linked to *RORC* in the other species which are compared. The gar *RORC* sequence is available at GenBank accession XP_006641800. More diverged syntenies not shown in the figure are: Human *ECM1* and *TPM3* both map to Chr. 1 within 3 Mb from the *RORC* locus, and zebrafish *ECM1* and *LINGO4* map to Chr. 16 at 8 Mb and at >10 Mb from the *RORC* locus. The duplicated zebrafish stretches on Chrs. 2 and 8 correspond with stretches on human Chr. 19p, and we do not agree with Flores *et al.* [7] that there is (sufficient) synteny supporting *RORC* identity of the respective genes on zebrafish Chr. 2 and Chr. 8.

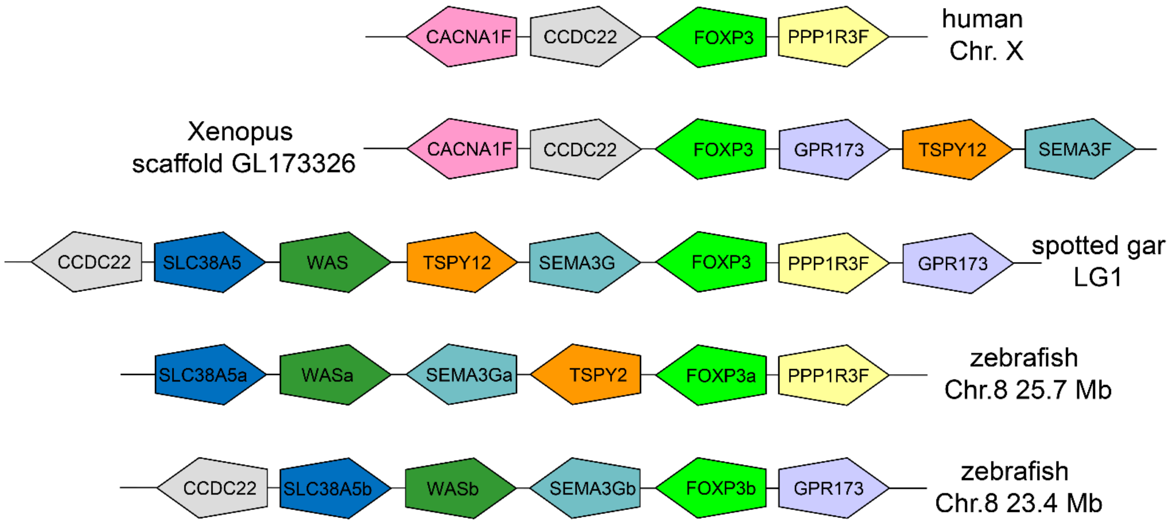

Figure S7. *FOXP3*. An elephant shark *FOXP3* candidate gene was identified in scaffold_4341 by Venkatesh *et al.* [1], but this very short scaffold does neither include the entire gene nor any neighboring genes and final identification may need additional information. For spotted gar *FOXP3* see GenBank accession XP_006625452. Zebrafish *FOXP3a* has been discussed by Mitra *et al.* [5] and Quintana *et al.* [8].

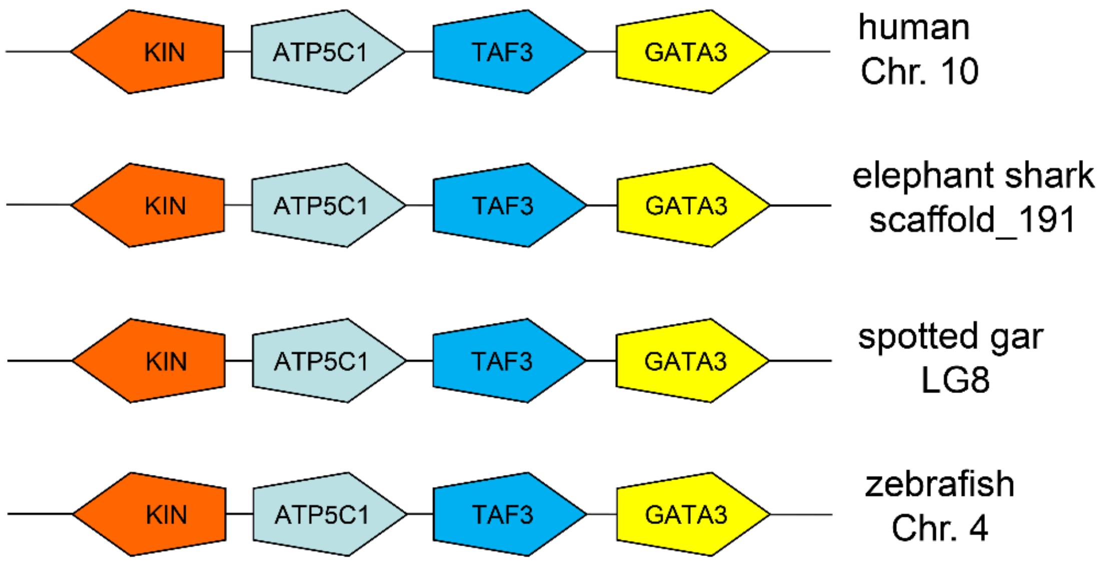

Figure S8. *GATA3*. The elephant shark *GATA3* gene was identified by Venkatesh *et al.* [1]. In the regions immediately downstream of human, spotted gar and zebrafish *GATA3* no coding genes are found. The elephant shark scaffold_191 sequence ends just downstream of *GATA3*. For spotted gar *GATA3* see GenBank accession XP_006633355. Zebrafish GATA-3 has been discussed by Neave *et al.* [9].

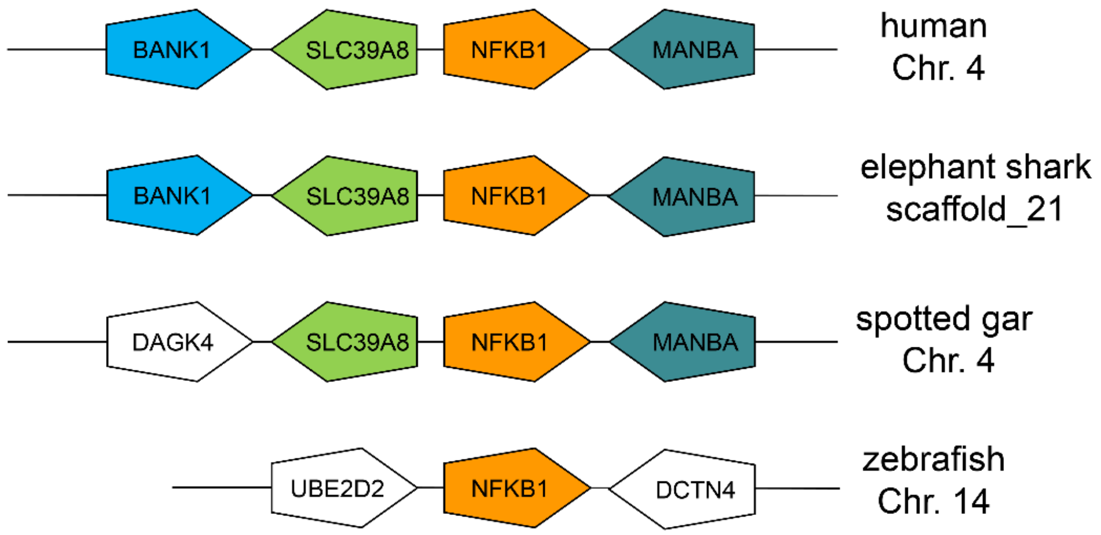

Figure S9. *NFKB1* (p105 precursor of NF-kB subunit p50). The elephant shark *NFKB1* gene was identified by Venkatesh *et al.* [1]. For spotted gar *NFKB1* see GenBank accession XP_006629913. Zebrafish *NFKB1* has been discussed by Pradhan *et al.* [10].

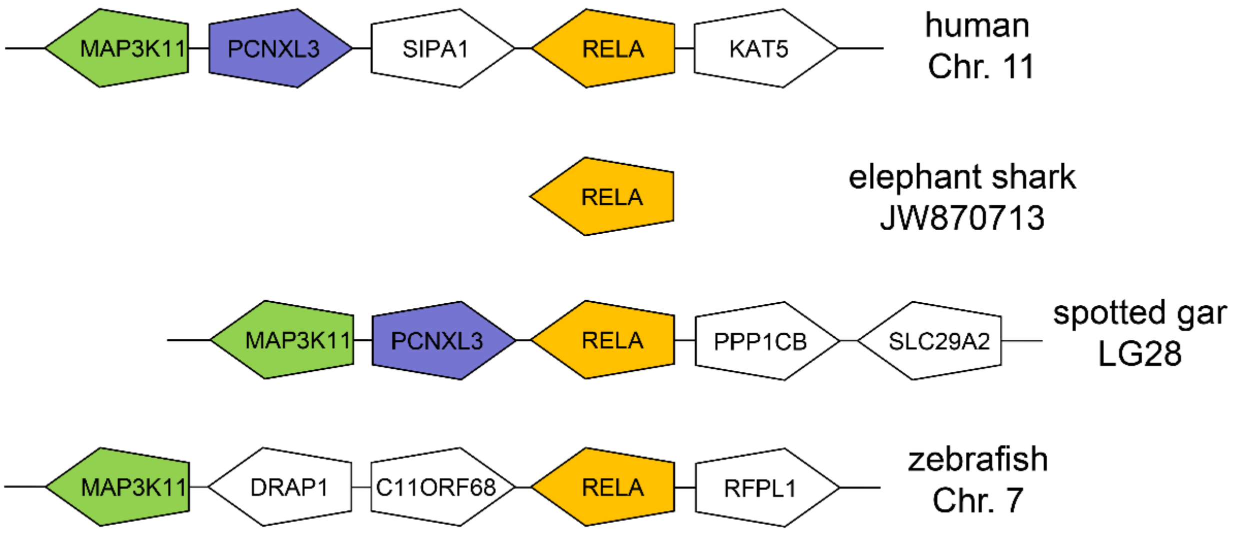

Figure S10. *RELA* (NF-κB subunit RELA, alias p65). The elephant shark *RELA* sequence was identified by Venkatesh *et al.* [1], but from a cDNA sequence and not from a genomic sequence. It is unclear whether spotted gar *RELA* is an intact gene, as non-sequenced gaps remain in the sequence stretch presented by the Ensembl database. Zebrafish GATA-3 has been discussed by Pradhan *et al.* [10]. More diverged syntenies not shown in the figure are: Human *SLC29A2*, *DRAP1* and *C11ORF68* map to Chr. 11 within 500 kb from *RELA*. Gar *SIPA1*, *KAT5* and *DRAP1* map to LG28 within 1 Mb, at 3 Mb and at 15 Mb from *RELA*, respectively. Zebrafish *SIPA1* maps to the other end of Chr. 7 than *RELA*. Zebrafish *RELA* has been discussed by Pradhan *et al.* [10].

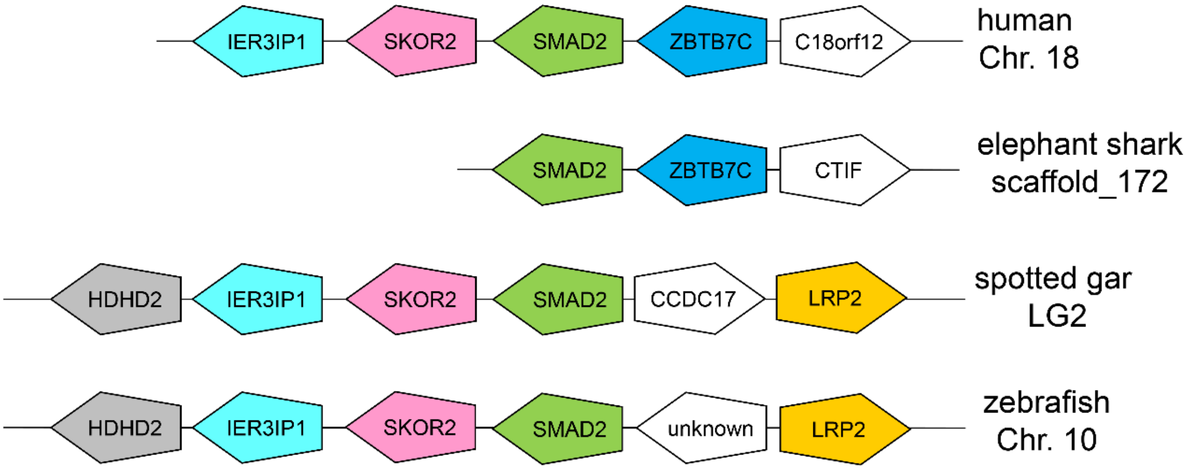

Figure S11. *SMAD2*. For elephant shark *SMAD2* see GenBank accession XP_007904996. Elephant shark *SMAD2* maps to the end of scaffold 172. For spotted gar *SMAD2* see GenBank accession XP_006627302. Zebrafish *SMAD2* has been discussed by Dick *et al.* [11]. More diverged syntenies not shown in the figure are: Human *CTIF* and *HDHD2* map to Chr. 18 within 1 Mb from *SMAD2*. Gar *ZBTB7C* and *CTIF* map to LG2 within 5 Mb from *SMAD2*.

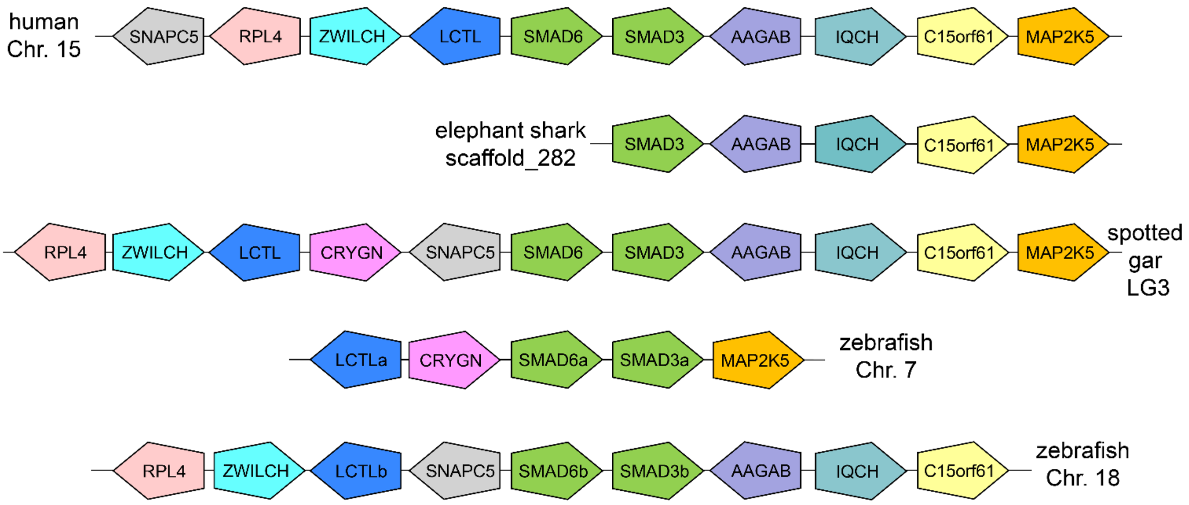

Figure S12. *SMAD3*. For elephant shark *SMAD3* see GenBank accession XP_006628801. Elephant shark *SMAD3* lays on the end of scaffold 282. For spotted gar *SMAD3* see GenBank accession XP_006628801. The duplicated zebrafish *SMAD3* genes have been discussed by Jia *et al.* [12].

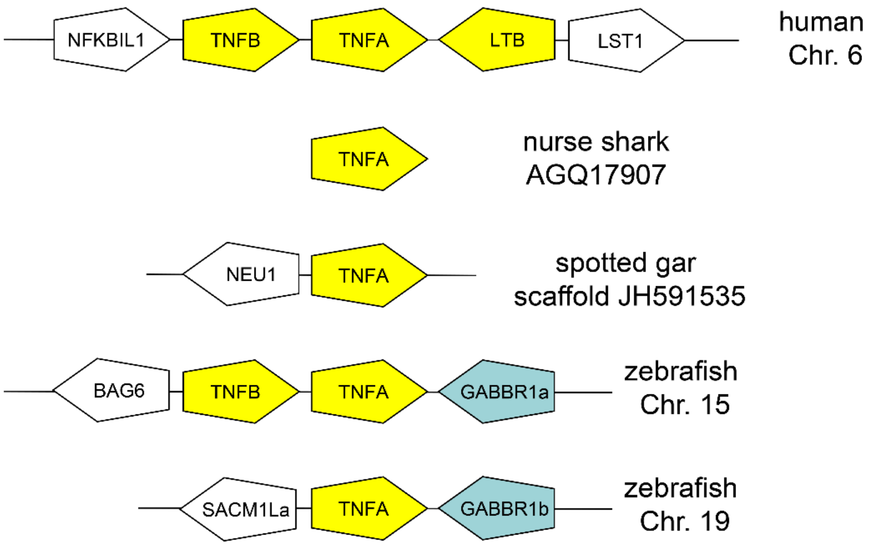

Figure S13. *TNFA* (TNF-α). In nurse shark an mRNA encoding a probable TNF-α protein was reported (GenBank accession AGQ17907), but an equivalent in elephant shark is not known. The spotted gar scaffold JH591535 is very short; for the spotted gar TNF-α sequence see GenBank accession XP_006643375. Human *TNFA* gene resides in the "Mhc III" region, an evolutionary unstable region. However, although the precise gene settings are not conserved, analyses from teleost fish indicate that the location of the TNF genes within the Mhc region is ancient (Sambrook *et al.* [13]; Ohashi *et al.* [14]; Hong *et al.* [15]). Whether the TNF genes in fish correspond to mammalian *TNFA* or *TNFB*, or are elsewise related, will need a thorough analysis. The zebrafish TNF genes are discussed by Savan *et al.* [16] and Hong *et al.* [15], and they name the here depicted zebrafish "*TNFB*" gene (Ensembl nomenclature) as "*TNF-N*". More diverged syntenies not shown in the figure are: Human *NEU1* and *BAG6* map to Chr. 6 within 0.5 Mb from *TNFA*. Zebrafish *NEU1* maps to Chr. 19 at 0.2 Mb from *TNFA*.

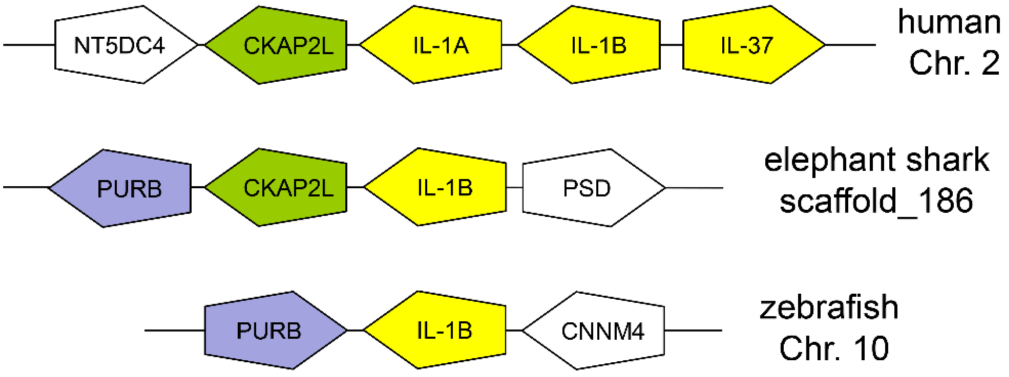

Figure S14. *IL-1B* (IL-1β). The elephant shark *IL-1B* gene was identified by Venkatesh *et al.* [1]. We could not find spotted gar *IL-1B* in the investigated databases. Zebrafish *IL-1B* is discussed by Huising *et al.* [17] and Wang *et al.* [18]. Also in some teleost fish species the *IL-1B* gene maps besides *CKAP2L* [18]. A more diverged synteny not shown in the figure is: Human *CNNM4* maps to Chr. 2 at 16 Mb from *IL-1B*.

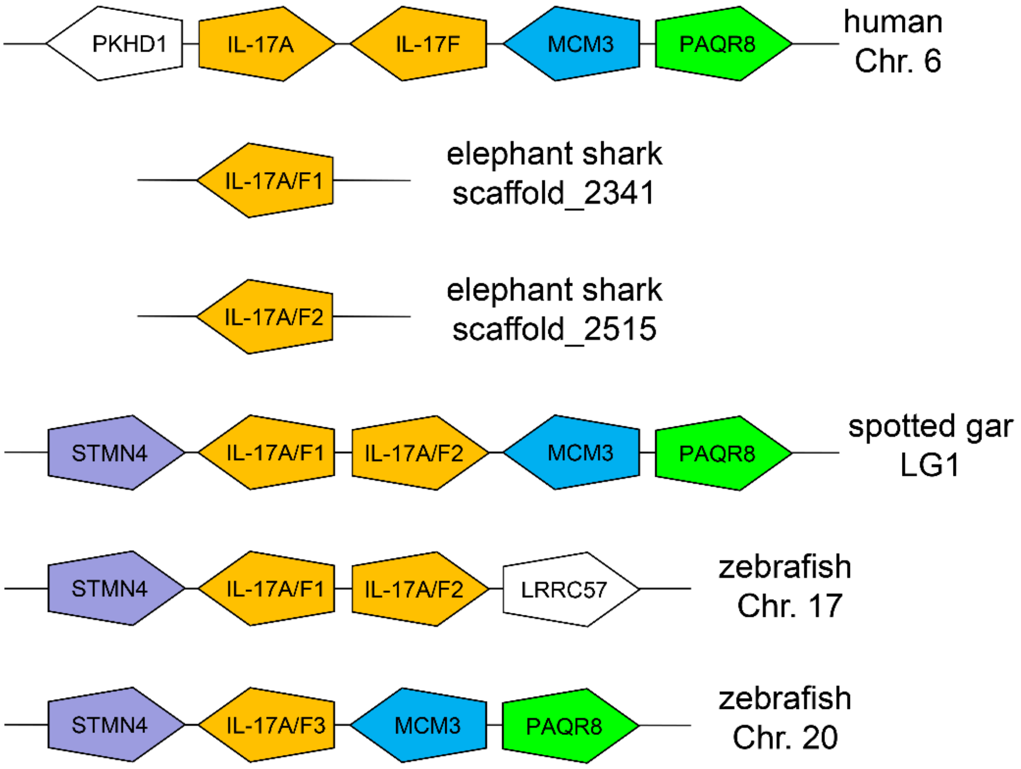

Figure S15. *IL-17A/F* (*IL-17A*, *IL-17F* and *IL-17A/F*). The elephant shark IL-17A/F genes were identified by Venkatesh *et al.* [1]; the scaffold sequences 2341 and 2515 are too short for synteny analysis. The spotted gar and zebrafish IL-17A/F sequences were discussed by Wang *et al.* [19].

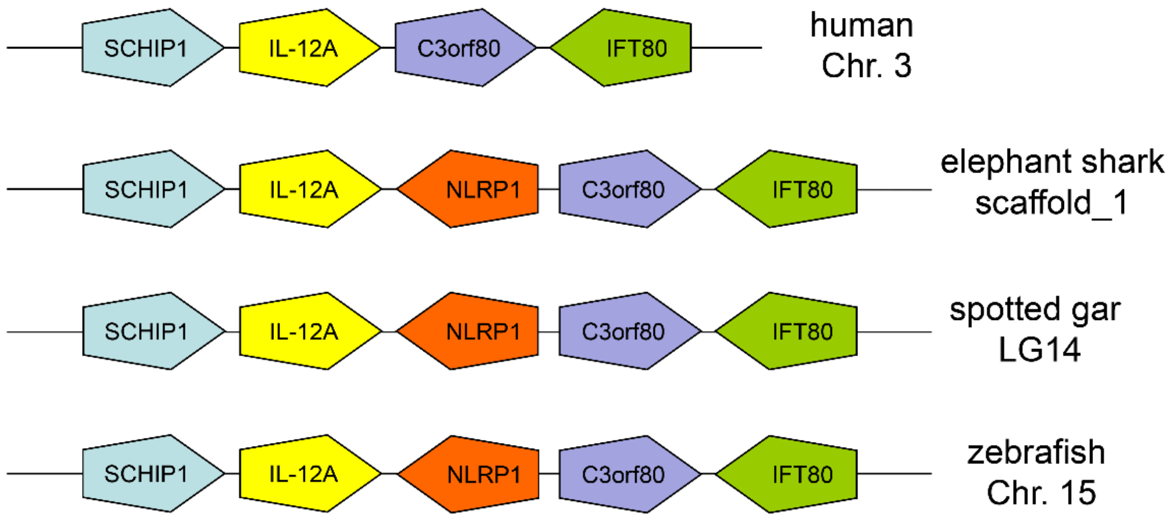

Figure S16. *IL-12A* (IL-12A subunit of IL-12). For elephant shark *IL-12A* see GenBank accession XM_007889808. For spotted gar *IL-12A* see Ensembl accession ENSLOCG00000003721. Teleost *IL-12A* was first described by Yoshiura *et al.* [20]. Zebrafish *IL-12A* has been discussed by Long *et al.* [21]. Zebrafish C3orf80 was found in the Ensembl database by blast similarity searches. *IL-12B* is not shown in this figure, but clear orthologues are found between mammals, elephant shark, spotted gar and teleost fish. However, lineage specific *IL-12B* duplications appear to have occurred, leading to more than one copy in for example elephant shark (Venkatesh *et al.* [1]), carp (Huising *et al.*) [22] and zebrafish (see the Ensembl database).

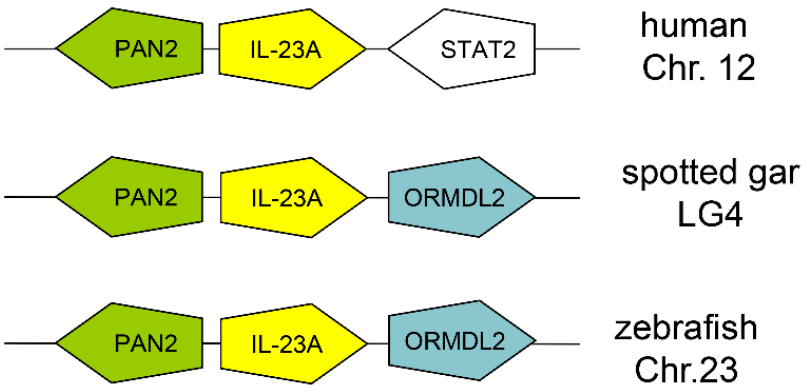

Figure S17. *IL-23A* (IL-23A subunit of IL-23). In elephant shark *IL-23A* has not been found (see also Venkatesh *et al.* [1]). The spotted gar IL-23A sequence might be MPSPSGAGAALLVCLVLSLTGLGEGRGDSIKEWDKFKTYSVELNGLARQMAKMLPDKSRLSHQ DVKGGVLLVESADRCDPLSLKSDPEPCLSKIAGALQNYSRLFGDRDLFKDSPWKELAQQVTT VIEHLLGLLGKPVSSGSLLGQESRWMFWQLQQYSVERLLSFSTIAARVFSPGDPATHDAGSQD SCPPS. Zebrafish IL-23A has been discussed by Holt *et al.* [23]. More diverged syntenies not shown in the figure are: Human *ORMDL2* maps to Chr. 12 within 1 Mb from *IL-23A*. Spotted gar STAT2 maps to LG4 at 6 Mb from IL-23A. For *IL-12B* gene, encoding the other component of IL-23, see Figure S16 legend.

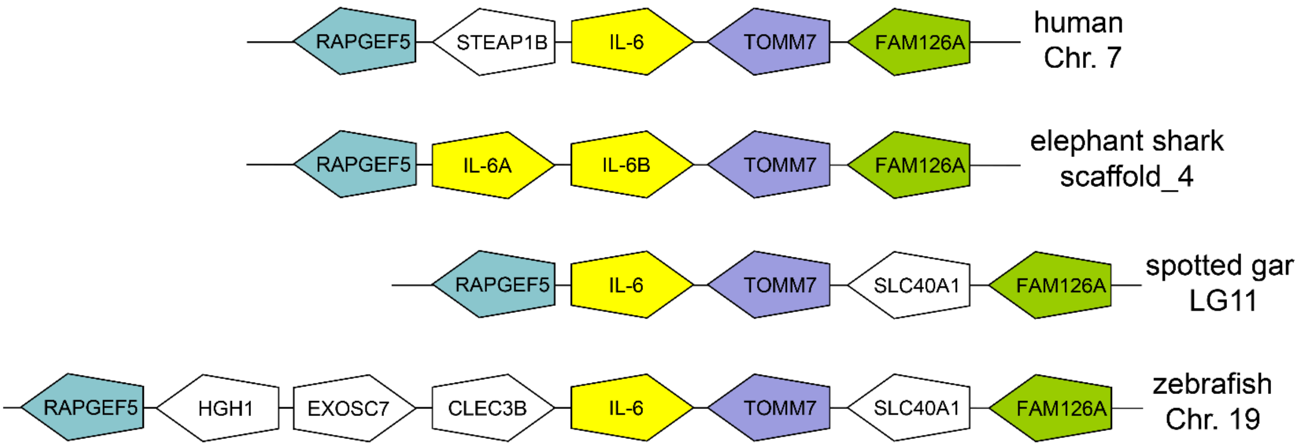

Figure S18. *IL-6*. The elephant shark *IL-6* genes were identified by Venkatesh *et al.* [1]; they appear to be derived from a recent duplication, and we tentatively named them *IL-6A* and *IL-6B*. The deduced partial spotted gar *IL-6* encoded sequence is CLDNFKDYKT ENETLSACKKHFDDQIVLSQIDGCLPSKFNKEKCLASLSTGLREYGVYLEQVRHL YPERTEDLQWNTQCLANDILRRTRSSRGVEELGGLQLRMGLRSDEGGRAWETQ KAAHIVFRELARFLQQAFRAVRFLNCRRQQC. Zebrafish *IL-6* has been discussed by Varela *et al.* [24].

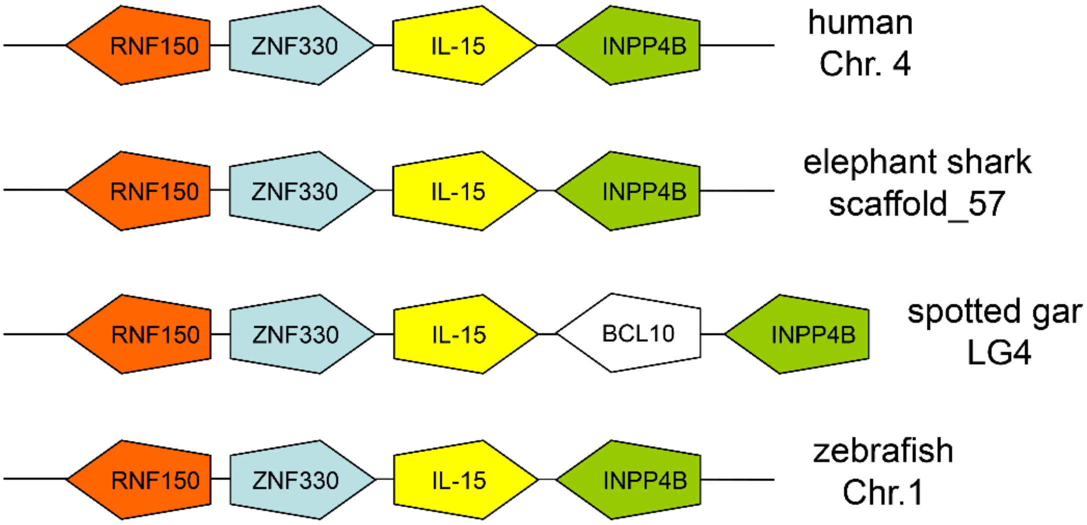

Figure S19. *IL-15*. The elephant shark *IL-15* gene was identified by Venkatesh *et al.* [1]. The deduced partial spotted gar *IL-15* encoded sequence is ALKSEHNHAFLQFFFFF CSGICHNRIWSIFRMCILSSCVYANMPTTEALDQALREMQTCLESNLSLLKSAGCVY YTPEDYDTDCTNTLFECYILEMEVIIFELDQDYESVHSLQTFLEHAKKKIFLHCKRCE EYEEKTAEAFLSKFQTFLQHLHSIGYNMTS. Zebrafish *IL-15* has been discussed by Gunimaladevi *et al.* [25].

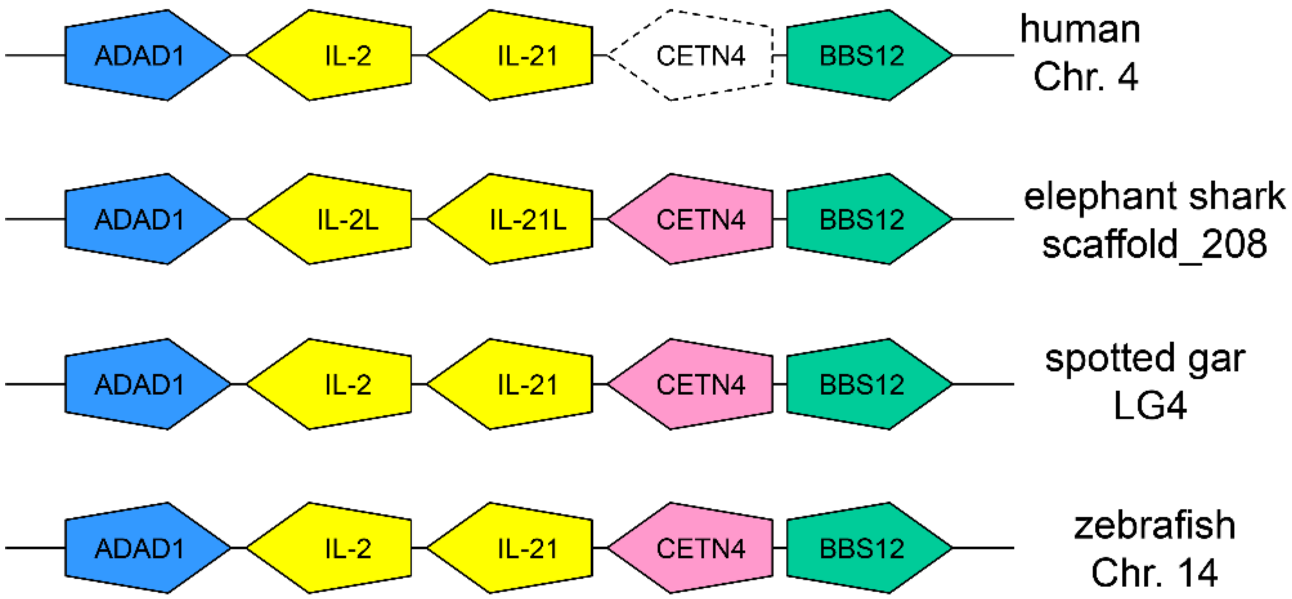

Figure S20. *IL-2* and *IL-21*. Human *CETN4* is a pseudogene. The elephant shark *IL-2L* and *IL-21L* genes were identified by Dijkstra [26]. The gar and zebrafish IL-2 and IL-21 sequences have been discussed by Dijkstra *et al.* [27].

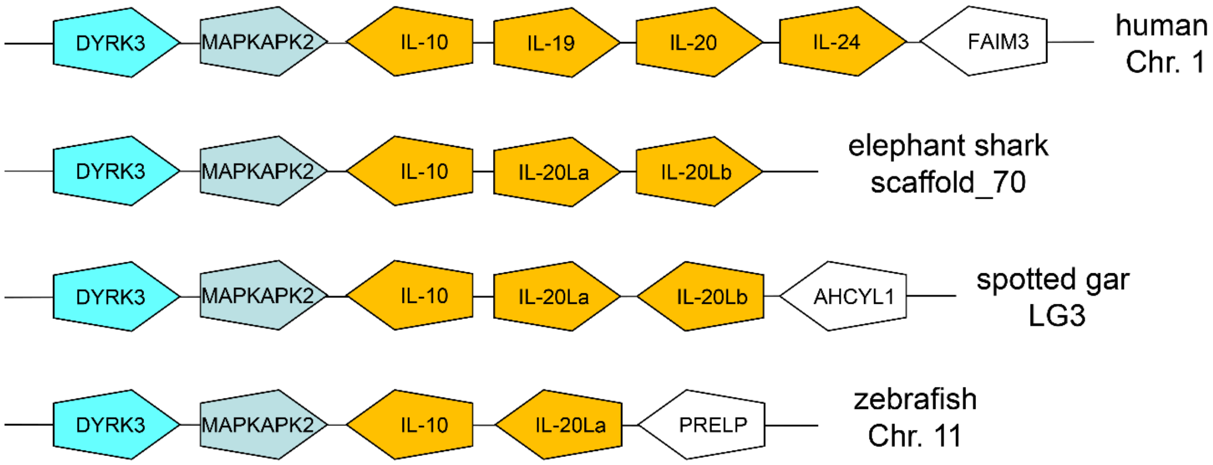

Figure S21. *IL-10*. The elephant shark *IL-10* gene, and the two related genes that we here designate as *IL-20La* and *IL-20Lb*, based on database top-matches and on nomenclature in Wang *et al.* [28], were identified by Venkatesh *et al.* [1]. For spotted gar *IL-20La* and *IL-20Lb* see Ensembl accession ENSLOCT00000015115 and GenBank accession XP_006628631, respectively. Zebrafish *IL-10* and *IL-20L* have been discussed by Wang *et al.* [28].

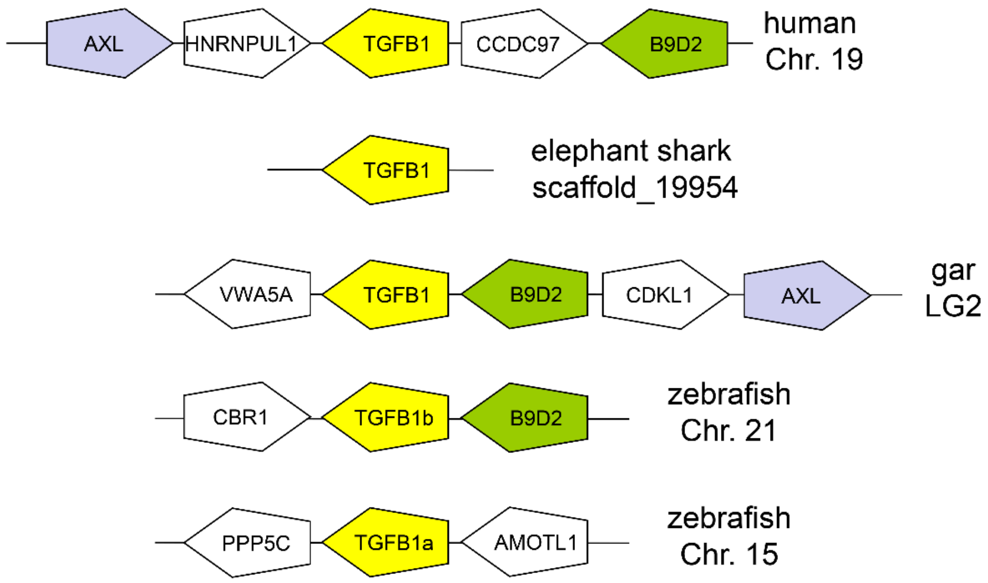

Figure S22. *TGFB1* (TGF-β1, TGF-β). The elephant shark *TGFB1* gene was identified by Venkatesh *et al.* [1], with a genomic fragment represented by the small scaffold 19954 sequence and the entire coding sequence known from cDNA analysis (GenBank accession JW872116). For spotted gar *TGFB1* see GenBank accession XP_006627576. The duplicated zebrafish *TGFB1* genes have been discussed by Maehr *et al.* [29]. More diverged syntenies not shown in the figure are: Human *PPP5C* maps to Chr. 19 at 5 Mb from *TGFB1*. Spotted gar *HNRNPUL1* and *PPP5C* map to LG2 at 0.5 and 5 Mb from *TGFB1*, respectively.

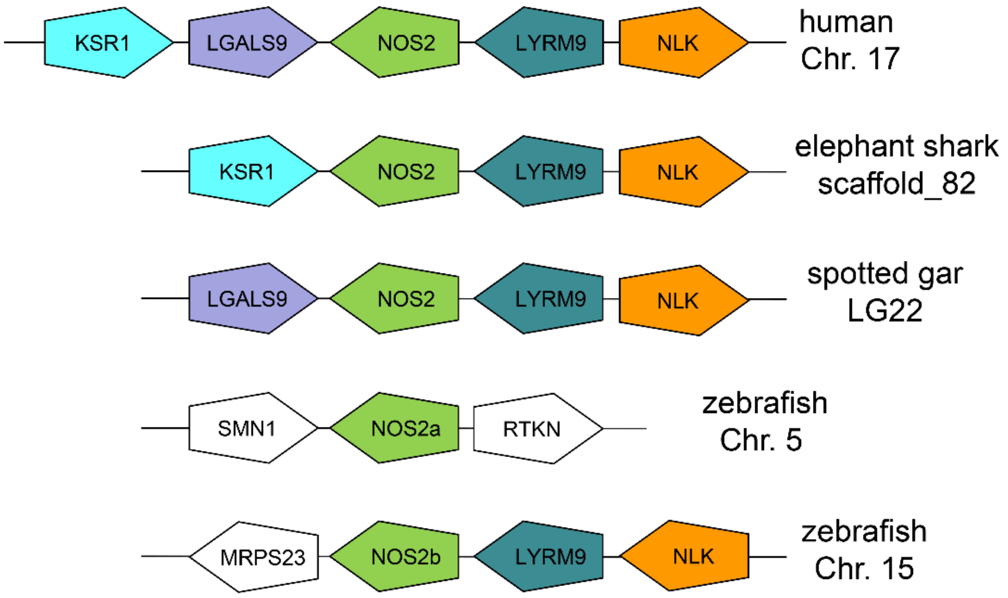

Figure S23. *NOS2* (iNOS). For elephant shark *NOS2* see GenBank accession XP_007898890. For spotted gar *NOS2* see GenBank accession XP_006640988. The duplicated zebrafish *NOS2* genes were discussed by Lepiller *et al.* [30]. More diverged syntenies not shown in the figure are: Human *MRPS23* maps to Chr. 17 at 30 Mb from NOS2. Spotted gar *KSR1* maps to LG22 at 2 Mb from *NOS2*. Zebrafish *KSR1* and *LGALS9* map to Chr. 15 within 1 Mb from *NOS2a*.

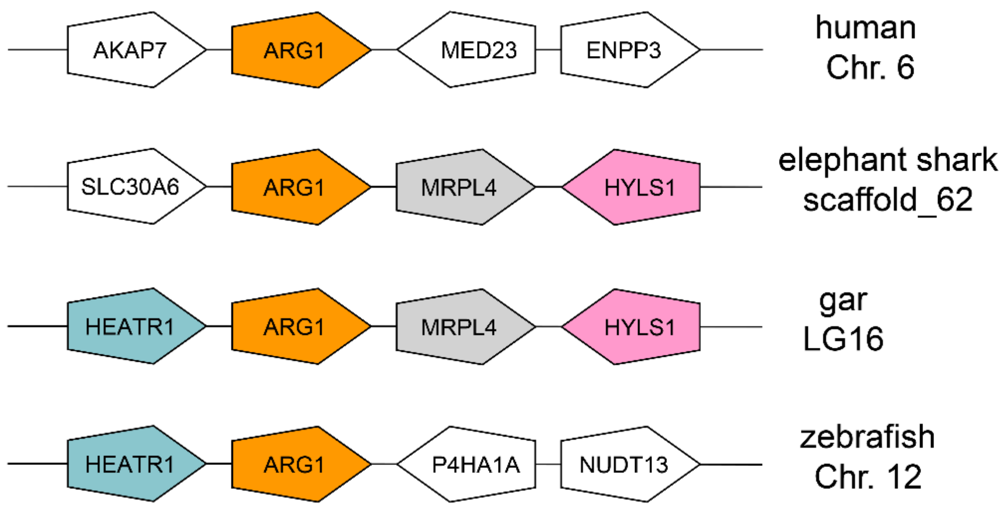

Figure S24. *ARG1*. For elephant shark *ARG1* see GenBank accession XP_007896670. For spotted gar *ARG1* see GenBank accession XP_006638730. Zebrafish *ARG1* has been discussed by Caldovic *et al*. [31].

References

1. Venkatesh, B.; Lee, A.P.; Ravi, V.; Maurya, A.K.; Lian, M.M.; Swann, J.B.; Ohta, Y.; Flajnik, M.F.; Sutoh, Y.; Kasahara, M.; *et al*. Elephant shark genome provides unique insights into gnathostome evolution. *Nature* **2014**, *505*, 174–179.
2. Song, H.; Yan, Y.-L.; Titus, T.; He, X.; Postlethwait, J.H. The role of stat1b in zebrafish hematopoiesis. *Mech. Dev.* **2011**, *128*, 442–456.
3. Lewis, R.S.; Ward, A.C. Conservation, duplication and divergence of the zebrafish stat5 genes. *Gene* **2011**, *338*, 65–74.
4. Gorissen, M.; de Vrieze, E.; Flik, G.; Huising, M.O. STAT genes display differential evolutionary rates that correlate with their roles in the endocrine and immune system. *J. Endocrinol.* **2011**, *209*, 175–184.
5. Mitra, S.; Alnabulsi, A.; Secombes, C.J.; Bird, S. Identification and characterization of the transcription factors involved in T-cell development, *t-bet*, *stat6* and *foxp3*, within the zebrafish, *Danio rerio*. *FEBS J.* **2010**, *277*, 128–147.
6. Takizawa, F.; Araki, K.; Kobayashi, I.; Moritomo, T.; Ototake, M.; Nakanishi, T. Molecular cloning and expression analysis of T-bet in ginbuna crucian carp (*Carassius auratus langsdorfii*). *Mol. Immunol.***2008**, *45*, 127–136.
7. Flores, M.V.; Hall, C.; Jury, A.; Crosier, K.; Crosier, P. The zebrafish retinoid-related orphan receptor (*ror*) gene family. *Gene Expr. Patterns* **2007**, *7*, 535–543.
8. Quintana, F.J.; Iglesias, A.H.; Farez, M.F.; Caccamo, M.; Burns, E.J.; Kassam, N.; Oukka, M.; Weiner, H.L. Adaptive autoimmunity and Foxp3-based immunoregulation in zebrafish. *PLoS ONE* **2010**, *5*, e9478.
9. Neave, B.; Rodaway, A.; Wilson, S.W.; Patient, R.; Holder, N. Expression of zebrafish GATA 3 (*gta3*) during gastrulation and neurulation suggests a role in the specification of cell fate. *Mech. Dev.* **1995**, *51*, 169–182.

10. Pradhan, A.; Khalaf, H.; Ochsner, S.A.; Sreenivasan, R.; Koskinen, J.; Karlsson, M.; Karlsson, J.; McKenna N.J.; Orbán, L.; Olsson, P.E. Activation of NF-κB protein prevents the transition from juvenile ovary to testis and promotes ovarian development in zebrafish. *J. Biol. Chem.* **2012**, *287*, 37926–37938.
11. Dick, A.; Mayr, T.; Bauer, H.; Meier, A.; Hammerschmidt, M. Cloning and characterization of zebrafish smad2, smad3 and smad4. *Gene* **2000**, *246*, 69–80.
12. Jia, S.; Ren, Z.; Li, X.; Zheng, Y.; Meng, A. Smad2 and smad3 are required for mesendoderm induction by transforming growth factor-β/nodal signals in zebrafish. *J. Biol. Chem.* **2008**, *283*, 2418–2426.
13. Sambrook, J.G.; Figueroa, F.; Beck, S. A genome-wide survey of Major Histocompatibility Complex (MHC) genes and their paralogues in zebrafish. *BMC genomics* **2005**, doi:10.1186/1471-2164-6-152.
14. Ohashi, K.; Takizawa, F.; Tokumaru, N.; Nakayasu, C.; Toda, H.; Fischer, U.; Moritomo, T.; Hashimoto, K.; Nakanishi, T.; Dijkstra, J.M. A molecule in teleost fish, related with human MHC-encoded G6F, has a cytoplasmic tail with ITAM and marks the surface of thrombocytes and in some fishes also of erythrocytes. *Immunogenetics* **2010**, *62*, 543–559.
15. Hong, S.; Li, R.; Xu, Q.; Secombes, C.J.; Wang, T. Two types of TNF-α exist in teleost fish: Phylogeny, expression, and bioactivity analysis of Type-II TNF-α3 in rainbow trout *Oncorhynchus mykiss*. *J. Immunol.* **2013**, *191*, 5959–5972.
16. Savan, R.; Kono, T.; Igawa, D.; Sakai, M. A novel tumor necrosis factor (TNF) gene present in tandem with the TNF-α gene on the same chromosome in teleosts. *Immunogenetics* **2005**, *57*, 140–150.
17. Huising, M.O.; Stet, R.J.; Savelkoul, H.F.; Verburg-van Kemenade, B.L. The molecular evolution of the interleukin-1 family of cytokines; IL-18 in teleost fish. *Dev. Comp. Immunol.* **2004**, *28*, 395–413.
18. Wang, T.; Bird, S.; Koussounadis, A.; Holland, J.W.; Carrington, A.; Zou, J.; Secombes, C.J. Identification of a novel IL-1 cytokine family member in teleost fish. *J. Immunol.* **2009**, *183*, 962–974.
19. Wang, T.; Jiang, Y.; Wang, A.; Husain, M.; Xu, Q.; Secombes, C.J. Identification of the salmonid IL-17A/F1a/b, IL-17A/F2b, IL-17A/F3 and IL-17N genes and analysis of their expression following *in vitro* stimulation and infection. *Immunogenetics* **2015**, *67*, 395–412.
20. Yoshiura, Y.; Kiryu, I.; Fujiwara, A.; Suetake, H.; Suzuki, Y.; Nakanishi, T.; Ototake, M. Identification and characterization of Fugu orthologues of mammalian interleukin-12 subunits. *Immunogenetics* **2003**, *55*, 296–306.
21. Long, Y.; Song, G.; Yan, J.; He, X.; Li, Q.; Cui, Z. Transcriptomic characterization of cold acclimation in larval zebrafish. *BMC genomics* **2013**, doi:10.1186/1471-2164-14-612.
22. Huising, M.O.; van Schijndel, J.E.; Kruiswijk, C.P.; Nabuurs, S.B.; Savelkoul, H.F.; Flik, G.; Verburg-van Kemenade, B.L. The presence of multiple and differentially regulated interleukin-12p40 genes in bony fishes signifies an expansion of the vertebrate heterodimeric cytokine family. *Mol. Immunol.* **2006**, *43*, 1519–1533.
23. Holt, A.; Mitra, S.; van der Sar, A.M.; Alnabulsi, A.; Secombes, C.J.; Bird, S. Discovery of zebrafish (*Danio rerio*) interleukin-23 alpha (IL-23α) chain, a subunit important for the formation of IL-23, a cytokine involved in the development of Th17 cells and inflammation. *Mol. Immunol.* **2011**, *48*, 981–991.
24. Varela, M.; Dios, S.; Novoa, B.; Figueras, A. Characterisation, expression and ontogeny of interleukin-6 and its receptors in zebrafish (*Danio rerio*). *Dev. Comp. Immunol.* **2012**, *37*, 97–106.

25. Gunimaladevi, I.; Savan, R.; Sato, K.; Yamaguchi, R.; Sakai, M. Characterization of an interleukin-15 like (IL-15L) gene from zebrafish (*Danio rerio*). *Fish shellfish immunol.* **2007**, *22*, 351–362.
26. Dijkstra, J.M. TH2 and T_{reg} candidate genes in elephant shark. *Nature* **2014**, *511*, E7–E9.
27. Dijkstra J.M.; Takizawa, F.; Fischer, U.; Friedrich. M.; Soto-Lampe, V.; Lefèvre, C.; Lenk, M.; Karger, A.; Matsui, T.; Hashimoto, K. Identification of a gene for an ancient cytokine, interleukin 15-like, in mammals; interleukins 2 and 15 co-evolved with this third family member, all sharing binding motifs for IL-15Rα. *Immunogenetics* **2014**, *66*, 93–103.
28. Wang, T.; Díaz-Rosales, P.; Martin, S.A.; Secombes, C.J. Cloning of a novel interleukin (IL)-20-like gene in rainbow trout *Oncorhynchus mykiss* gives an insight into the evolution of the IL-10 family. *Dev. Comp. Immunol.* **2010**, *34*, 158–167.
29. Maehr, T.; Costa, M.M.; Vecino, J.L.G.; Wadsworth, S.; Martin, S.A.; Wang, T.; Secombes, C.J. Transforming growth factor-β1b: A second TGF-β1 paralogue in the rainbow trout (*Oncorhynchus mykiss*) that has a lower constitutive expression but is more responsive to immune stimulation. *Fish shellfish immunol.* **2013**, *34*, 420–432.
30. Lepiller, S.; Franche, N.; Solary, E.; Chluba, J.; Laurens, V. Comparative analysis of zebrafish nos2a and nos2b genes. *Gene* **2009**, *445*, 58–65.
31. Caldovic, L.; Haskins, N.; Mumo, A.; Majumdar, H.; Pinter, M.; Tuchman, M.; Krufka, A. Expression pattern and biochemical properties of zebrafish *N*-acetylglutamate synthase. *PLoS ONE* **2014**, 9, e85597.

© 2015 by the authors; licensee MDPI, Basel, Switzerland. This article is an open access article distributed under the terms and conditions of the Creative Commons Attribution license (http://creativecommons.org/licenses/by/4.0/).
