# Supplementary material for: Along the Axis between Type 1 and Type 2 Immunity; Principles Conserved in Evolution from Fish to Mammals
Source: Biology (Basel). 2015 Nov 17;4(4):814–59. doi: 10.3390/biology4040814 (PMC4690019; doi:10.3390/biology4040814)
Supplement: Supplementary File 1 [file biology-04-00814-s001.zip › biology-4040814-supplementary 2.pdf]

Supplementary Materials

Supplementary file 2

(Predicted) ORF Sequences of the Immune-Signature Genes Analyzed in Table 1.

In cases where GenBank accession numbers are not given, the sequences were assembled from SRA reads.

Elephant Shark

T_H1-Signature Genes

T-bet (XM_007909399)

ATGTTCCCGTTCCTGAGCTTTAACATCACGGGGCTGGAGCCCACGGCTCACTACAATGTCT
TTGTGGACGTGGTCCTGGCTGACCAGCATCACTGGAGGTACCAGGGCGGCAAGTGGGTGC
AGTGTGGCAAGGCGGAGGGCAGCATGCAAGGTAACAGGACGTACATGCACCCAGACTCG
CCCAACACTGGAACCCATTGGATGCGGCAGGAAATCTCGTTCGGGAAACTCAAACTGACG
AATAACAAGGGAGCGTCCAACAATGTCACTCAGATGATCGTGCTCCAGTCGCTGCACAAG
TACCAGCCACGGCTGCACATTGCGCCGGTGAAGGAGGACGGCTCTGACGACCCCTATCTC
ACCCCCAAGCTTCAGGCCTTCAGCTTCCCCGAGACTCAGTTCATCGCTGTCACCGCCTACC
AGAATGCAGATATCACACAGCTCAAGATTGATCACAATCCCTTTGCCAAAGGATTCCGAG
ACAACTTTGACTCGTGA

STAT1 (XM_007890161)

ATGTCTCAGTGGTATGAGTTACAACAGTTGAATTCCAAGTTCCTGGAGCAAGTCGATCAG
CTTTACGATGACAACTTTCCAATGGAAATCAGACAGTATCTTGGACATTGGCTGGAGACC
CAGGATTGGGAGCACGCTGCTACGAATGACTCCATGGCCACTCTGCTCTTCCATGAAATG
TTGGTGCAGCTGGATGATCAGTACAGCCGCTTCACCTTGGAGAACAACTTCTTACTACAG
CACAACATCAGAAAAATCAAACGCAATCTTCAGGTTTTCTTCCAGGAAGATACTATTTATA
TGGCGTTGACGATATCCAACTGCCTTAAGGAAGAAAAGAAAATTCTAGACAATGCTCAGA
AAGCTGAACAGAACCACATTGGTCATATCCAGAACACTGTTATCCAGGATAAACAAAAG
AAGCTTGACCAGAAAGTGAGAAACGTGAAGACTGGCGTCCAGGAAATTGAACAGGAAAT
CAAATCTCTTGAAGATCTTCAGGATGAATTTGACTTCAGATCCAAAACGCTGCAGAGTCG
AGAGTTGGAAGTGAACGGCTTGGCTCAAAATGAGATTAAACAAGAAAAGCTTCAAATCC
ACAACATGAAGTTGACTCTGGATAAGAAACGAAAGGAAGTGATCAACAAAACCGCTTGTC
TCCTGAATGAAACGGAACAGATTAATGGCATTCTGATCAACGAAGAACTATTGGAATGGA
AACGTCGGCAGCAGATTGCTTGCATCGGAGGACCTCCAAACACTTGTCTTGACCAGCTCC
AGAACTGGTTCACACTGGTGGCTGAGAGTCTGCAGCAGGTTCGACAGCAGCTGAAAAAG
CTGGAAGAATTGGAGCAGAAATATTCGTACGAAGGAGACCCCATCACCCAAAACAAGCAG
TTCCTAGAGGAACGAAACAACAACTTGTTCTGCAACCTTATTGTGAGTTCCTTTGTGGTAG
AGAGGCAGCCTTGCATGCCCACCCACCCCCAGAGGCCGATGGTCCTGAAGACCGGCGTCC
AGTTCACTGTGAAGCTCAGACTCCTGGTCAGGCTCCCCGAGCTAAACTATCAGTTGCGGG
TGAAGGCGTTATTTGACAAGGATGTCACTGAAAAGTGTTCGGGAAAGGGATTTAGAAAAT
TTAACATACTTGGGACAAACACAAAGGTCATGAACATGGAGGAATCAACTAATGGAAGT

TTGGCTGCAGAGTTCCGACATCTGCAACTGAAGGAGCAGAAAAATGCAGGCACCAGAAC
GACTGAAGGCCCTCTCATTGTGACTGAGGAGCTTCACTCCATCAGCTTTGAGACTCAGTTC
TGTCACCAAAACCAGATAATAGATCTTGAGACTACATCTCTCCCGTTGGTTGTCATCTCCAA
TGTTAGCCAGCTCCCCAATGGCTGGGCCTCTATTCTCTGGTACAACATGCTCGTAAGCGATT
CCAAGAACTTGGCATTTTTCAACAACCCCCCAGTTGCCAAGTGGAGTCAGCTGTCTGAGGTA
CTGAGCTGGCAGTTCTCCTCGGTCACCAAGAGAGGCCTTAGCGTCGAACAGCTCAGTATGC
TGGGCGAGAAATTGCTAGGTCTGAATAACTGCTTCAATGATAATGGGATTCCATGGACAA
GATTCTGCAAGGAGAATCTAAATGAAAAACCTTTCTCATTCTGGCTCTGGATCGAAGGGA
TTTTAGAATTGATTAAAAAACATCTTCTTCCTCTCTGGAATGATGGCTGTATCATGGGGTT
CGTAAGTAAGGAGAAGGAGCGAACTTTGCTGAAGGACAGAAACCCGGGAACCTTTTTACT
GAGGTTCAGTGAGAGCAGCAGAGAAGGTGCCATCACGTTTACCTGGGTAGAGAAGTCAC
CAACTGATGAGTCTCAGTTTCATTCGGTGGAACCGTACACAAAGCATCAATTATCCGCAG
TTACCTTCCCAGACATCGTTCGGAATTACAAAGTCATGGCAGCCGAGAACATTCCGGAAA
ATCCACTGAAGTACCTGTACCCTGATCACCCCAAAGATCAGGCATTTGGAAAGTATTACTC
CAGACCAAAAGATCCTGCAGAGCCTATGGATGTGGATGATTCCAAGACCAGTGGTTACAT
AAAGACTGCACTCATTTCAGTGTCTGAAGTTCACCCATCTAGGCTGCAGTCAACAGAAAAT
CTTCTACCTATGTCCCCAGAAGAGTATGGTGAACTCGAACGTCAACTGAGTCCATCTGAAA
TGGACACAGTGATGTGCTCATATTCAAATTAA

<u>STAT4 (XM_007890162)</u>

ATGTCCCAGTGGCATCAACTGCATCAGCTGGACTCCCAGTTCCTGGAACAAGTGGACCAG
ATGTACGATGACACTTTTCCCATGGAAGTCCGACAGTTCCTAGCACATTGGTTGGAAAACC
AAGACTGGGAACTTGCGTCAAGGAATGAATCTTTGGCCACACTTCTGTTTCATAATTTATT
AAAACAAATAGACGACCAGTACAGCCGCTTCTCTATTGAAAAAAACTTCTTACTTCAGCAC
AATCTGAAAAGAATTAAACAAAACCTTCAGGTACAGTACCTGGGGAACCCTTTGCACATG
GCATCAGTTATCGCTGCCTGCTTGAGGGAAGAGCAGAGGATTCTGGCAGCAGTGAACACA
CCGGAGCAGGGGGACGAGGAAAAAATCATGCAGAACTCAATGATTTCGGAAAGACAGAG
AGAACTGGAACAGAGAATGACAGCCATGAAATCCAATGTACAGGTGGTCGAACAGGATG
TAAAGTTCCTCGAGGACCTGCAGGATGAATTTGATTTCAGATACAAGACACTACAGACAC
AAGAACACTGTGATAGGAACAGTATTCATGTGAAGGAGGAAGTTTTCCGGTTACAAGGAA
TGCTTAACCAACTGGATCATAAAAGAAAGGAGATTCTCCAAAAAATCACAGAACTGTTGA
ACCAAATGGATTTTCTCATGAACAGACAACTGATAGAAGAGCTGATAGAATGGAAGCGG
CGACAGCAGATCGCCTCCATCGGAGGGCCTCTGAACACATGCCTCGATCAGCTGCAGAAC
TGGTTCACGATGCTAGCAGAGAGCCTGTTTCAGGTCAGGAGACAAGTGAAAAAACTGGA
AGAGCTGCTGCAGAAGGTGACTTATGAAGGTGATCCTGTTCCACTGCAGAGACCCCACTT
GGAGGAAAAAGTAAACATTTTGATCTACAGCCTCATCAAAAGTTCCTTTGTGGTGGAAAG
GCAGCCTTGCATGCCCACCCATCCCCAGAGACCCATGGTCCTCAAGACTGGAGTCCAGTT
CACTGTCAAAATAAGGTTTTTGGTAAAATTGCCCGAACTGAACTATCAGCTAAAAGTGAA
AGCTACAATTGACAAGGAAAGTCTAACTGCAAGAGGCTATCGTAAGTTCAATATCCTAGG
CACTAATTGCAAAGTAATGAACATGGAGGAGTCAGTGAATGGAAGCCTTTCTGTGGAATT
CAGGCATTTGCAACTCAAAGAACAGAAATCCTCTACAGGAACCAAAGGAAATGAGGGTCC

TCTGATTGTGACAGAAGAGCTCCATTCAATCAATTTGAGACACAGTTTTGTCACCAGGAT
TTAACAATAGATTTAGAGACCAGTTCTTTGCCTGTCGTAGTCATCTCCAACGTGTGTCAGC
TACCCAGCGGCTGGGCCTCTGTTATATGGTACAACATGCTGATGAACGACCTCAAGAATTT
GGCTTTCTTCACCAACCCGCCTGCTGCCATTTGGAGTCAGCTTTCAGAGGTGCTGAGCTGG
CAGTTCTCATCTTATGTGGGTCGAGGTCTCAATTCTGACCAACTAAACATGTTGGCAGAG
AAACTACTTGGACAACAACTGAATTATAATGATTGCCAAGTTTCGTGGAGTAAATTCTGT
AAGGAGACCCTCCCTAATCGGTTCAGTTTCTGGCTTTGGTTGGATGGGATCTTGGAACTG
ATCAAGAAACACCTTTTGGCTCTCTGGATAGATGACTACATAGTGGGATTTGTGAGTAAG
GAGAAGGAGCGAGCTTTACTGAAAGACAAGCTGCCAGGGACGTTTTTACTGAGGTTCAG
CGAGAGCAGCAGGGAAGGCGCTATCACATTTACTTGGGTGGATCGGTCAGAGAATGGGG
AAATGAGACTTCATTCAGTGGAACCTTACTCCAAGAGTCAGCTGTTAGCTGTTCCTTTGC
CTGACATCATTCGCGACTACAAAGTAATGGTGGCTGAGAACATTCCAGAGAACCCCCTGC
AGTACCTGTATCCTGACATCCCGAAGTATAAAGCCTTTGGCAAACATTACAACAATAAGC
CGAAAGAAGTTTCTGAGCCCACAGAGACAGAGAAGAAAGGGTATATCTCCTCAAGGTTT
ATCCCGTTCTCTGAATTTCCAAATGCTTCCACAGCCCCAGTTTCCTCATCAGACCTACTTC
CCATGTCACCGGGAGTATATGCTGAACTGAAACAGCAACTGAGTCCAGCAGAAATCGAG
ACTGCTGTATGTTTTGAAGAAATGATTTGA

IFNγ (XM_007904994)

ATGAAGCTGTGTTTCATCATTGGGTTGGTCACTTGTCTACTATTACTGGGCTCTGCGAGCT
GTGACCGTCTGCAGGACGAAATTAATAACCTGAAATCGCACTTTGATACAAGCAGTCATG
AGGTGGCTGACGGTGGGCCGCTATTTTTGAATATGCTGGAGAAGTGGAAGGGCTCGCCTG
AATCGGGGATTGTGTTTCATCGGATACTACTCTGGTACGAAAATTTCTTTAAAAACATCAA
AGGGGCTCCTGGAAAGAACAAGGAACTGGACATTAGCAACATTGGGAATCTCATTTCTAA
GTGGATAGTAGAGGACAGATACAAACCAGTGGAACTGAAATATGATCTCGATAAATTAGA
CAATATTCAGTGGAATAAACAATTAGTCCAACGCAAAGCAGTCCTTGAACTTGAAATGCT
ACTACCAAGAATGAATGTAAATGGGAGAAGAAGACGCAGAAGCAACATGGGAGGTAGA
AGGCGATCAAGGACTTAA

TH17-Signature Genes

IL-17A like 1 (SINCAMT00000016888)

ATGGTGATGTTGAAATGGGTGCTGTGCTTTGTGCTGGCCATCACTCTGCCGTGTGTGTCAG
CACGAGGAGCTCACAGGAGAGGCTGTGGGAGACAAGTCGGCCGGAGGTGCTCAAAGCAG
CAGAAGTTCATGCTGAGGTTCAGAGAAGCAAAGCCCCTGTCAACCTGGCTCCCTGGGAAC
TCTGGCTATAGCAACCGCTCCATCTCCCCCTGGGTCTACAGGAAAGATGTTGACCCTGACC
GGTACCCAGACATCATAGCGATGGCGGAGTGTATCCACACTCACTGTGTCACTGCTCAGGG
GCACCTGGACCTGGGCCTGAACTCACGCCCAGTGCAGCAGGAGATGGTAGTGCTGAGGAG
GCAGCGCCATGGCCACAAGCTCACCTACCTGGTGGACAAGATGGTCGTTCCCATCGCCTG
TGTGTGTGTCCGTCCCCAGTTAGTTCAGGCCTGA

IL-17A like 2 (SINCAMT00000018117)

ATGATGGTTAAAGCCGCCCTGAGCTTTGTGCTGGTCATGGCTGCGCTGTGTGTGTCGGCG
AGAGCTGCTCCCACCAAAGGCAGTGGGAAAGCAGACCAGGAGGGGGGCCGGGAGCGAG
GCCGCTACCAACATCAGCCAAAGTACACGCTGAGCTTCAGAGAGAGCCCCGGGAGCAGC
GACTGGAGCCGAGGTCACAGCCAGGACCAGGGCCACGGCCAGGGACATGCAGGCATCAA
AAGCCGGTCCATCTCACCGTGGATTTACAAGAACAATTATGACCCTGATCGATACCCAGC
CACCATCTGGGAGGCTCACTGCGTCCACTCACACTGTATCAACGCCAGAGGCAAACTGGA
TGAAGACCTGAACACACAGCGAGTCTGGCAGAGAGTGCTGGTCCTCAGGAGAGAGTCCA
GCGCCAGCAAACTCAACTTCCTGGTGGAGGAGATACCGGTACCAGTCGCCTGTGTGTGTG
TCCGCGCTCGCGTTCTCCAGGCCTGA

IL-21L (Dijkstra *et al.* 2014 [1])

ATGAACAGGATCATCTTTTACAGCTTCATCCTCGCTGCTTTCATTGCTGTTCTTCATGCAGC
TCCCAGACGTGATCATGTGGTTCTGAAAGAGATGATGAAGGATGTCCGAAATTTGAAAAA
AATGTTTGGGCAATCTGGCGTTACAGTTCGCACTACAGAAAGCAAATTGGATAACCTTTCC
TGTTACATGGCCGAACTAAAAGGCCTCAAGAAATCCCGGATGCAAATTAATACCGTAGAA
AAACTGAACAGAAAAGCAAAACAGCTGAACCTTTTAAATATCGACTGCCCCAACAGTGAA
AATTACATGAAGAACAGCCTCGAACAGTTCCTGAATCAATTTTATGTACATCTGCAAAAG
AAATTCCATGCAGCTGTCAAGGTCGGTCAGCAGCTCAATAAAGTTCAGGTGAACATTTAA

IL-22 (XM_007904995)

ATGCCTTGCCTTTGGAGTCCAGCAGTATTGGTCCTTGGCTATGCCACTCTCCTGGTCAACT
GTGCACCGTGTAAGGAGTCTTGCCATGTGGATAAATATTACATGCATTTCATGCGACCCA
CTATCTTTGACCTGTCAAAAGAGGCTCAAGAGCATGACAAGGATACAGCAACAAGACTGAT
AGGAAAGAAGCTGTTCCAGGGTCTTCAGGAAGAGGACAAATGCTATCTGCTCCACAAAGT
TGTTGACTTCTATTTGAGAAATGACCTGGCATCTGAAGAACTGCACAGGAAATATTCACAT
ATCAAAAAAGTCAGGGAGGCGTTTACCACTCTGAAAATGAGCATATCTAAATGTGATGTT
CAAGAAATGAAGAAAGTAGATCACAAACTTGAAGAGCTAGAACGGGAAGTGAGGAAGCT
CGGAAAGAACAGAGAGGCCAAGGTTGTAGGAGAGCTGTACATGCTATTTCAGGATATAGG
GAAATATTGTTCAAAACCAAGAACTGGAAAGAAAGATTGA

T_{reg}-Signature Genes

Foxp3 (XM_007885996)

CTAACCGCTGAGCTGCAGGACGGCACAGATGAAACGTTAACGTGGATTAACCCTTTCCGT
TCCTCGCCACAGAAACCAGGGGAGGCCGGAGCGAGCAGCGTGTTCGAGGAGAACAGAGT
GGGTGTCAGAAACAGCGGGAAAGCTTGGACTCAAGTCTGGGACAAACACACCACTCTCT
ACCCAGACGTGAGTTCGAATCTGGAATATTACAGAGTCTATAATATCAGGCCACCGTTTA
CATACGCAGCTCTGATCAGATGGGCTATTCTGGAGACCAGTGAGAAACAGATGACTCTGA
ACGAAATCTATCTCTGGTTCACAAGGAAGTTTGCTTTCTTTCGCAACAACACAGCTACCTG

GAAGAACGCGGTGAGGCACAATCTGAGTCTCCACAAGTGTTTGTGAGAGTGGAGAATAT
GCGAGGAGCTGTGTGGACAGTGGACGAAATGGAGTTTCAGAGACGGAAATCACCGAAAA
TCACCAGCAGAGACATGCAAATGAGATGGGAGTCGCACTAA

IL-10 (XM_007899549)

ATGCAGCGTGCAACTGTTGTTCTGTGGATGCTGCTCACGCTGTTTAATATTCAGTGTTGCT
GTTGCAGTCATAGACAGGCAGCTGGGCTATGCCTTAGCACATCTGTGCTGCATATTCATCT
CAGAGAACTCAGGACAGCTAATCAGAAAATAAGCAGCTATTTTCAAAGGAGAGACGGCA
ATATTCGTGTATCGCTGCTGAGAGGGAAATTTCAGCATGAATTTAAGGGTCGTTTAGGAT
GTCAGTTTTTGAAGGAAATGTTAGATTTCTACCTCAGTCACATAATCCCAGCCGCCAAGAC
GCAAAGCAAAGCCTACAACACCCACATCAGTAAAATCGGGAACACGCTGTTCGAACTAC
AACAGAACATAGAGAATTGCCACCAACTCTTTAACTGTGACACCTGCAACTGCAATCGCA
GTAATTACATCGAGGAGATATACAACATGTACAAGAAGTTGCAGGATAAAGGTATATAC
AAGGCTATGGGAGAGCTCAACACCTTTATTGACTGGCTCGATAGGTTTCTGTCGATGAAA
CTGAAAGGAGCGAACAAGAAGAGAAAGTGA

TGFβ1 (fragment on scaffold_19954)

ATGCATAACCTGTCCTCCCACAAAGACGAAAAAAACTGCCGCGTCCATCAGTTGTACATA
AACTTCCGGAACGATTTGGATTGGAAATGGATCCACGAACCCAAAGGATATTACGCCAAT
TTCTGTATGGGGCCCTGTCCTTATATCTGGAGCGCAGACACCCACCA

T_H2-Signature Genes

GATA3 (XM_007885971)

ATGGAAGTGGATCAGCCGCGTTGGATGACACATCACCATCCAGCGGTGTTAAATGGACAG
CACCCGGATTCTCACCATCCCGGGCTCAGCCATGGCTATGTGGACTCCTCGCCCTATCTCC
CGTCCGAAGAAGTGGATGTACTGTTCAACATCGACAGCCAAGGCAACCACGTCTCGTCTT
ATTATGGCAACTCGGTCAGAGCCGCCGTTCAGAGATATCCACCGCCACATCACGGGAGTC
AGGTCTGCCGTCCTCCCTTGCTTCACAGCTCCCTGTCCTGGATAGATGGAGGCAAGGCTCT
TGCCTCCCACCACAATGCTTCGCCATGGAATCTGAGTGCTTTCAGCAAGGCCCCCCTTCAC
CACGGCTCCCCGGGGGCTCTTTCAGTCTACCCTCCATCCTCCTCCTCGTCCCTCTCCACAG
GACATTCCAGCCCCCACCTGTTCACCTTCCCCCCGACCCCACCCAAGGATGTCTCCCCCGA
CCCTAACATCTCCGGTGCTGGATCCAACGGCTCGGGCCGACAGGAGGACAAGGAGTGCGT
GAAATATCTGACCGAGGGAATGAAACTGGAGACATCCTCTCACCTGAGAGGCAGCATGG
GATCACTAGGAGGAGGTGCGAGCTCGGCACATCACCCCCTGGCTTCTTACCCGCCTTACG
TCTCAGAGTATGGCGCTGGGCTGTTCCCCCCTGGCAGTCTGTTGGGTGCGTCTCCCCCAGG
CTTCCCTTCGAAAGCAAGACCCAAGGCCCGCTCCAGCACAGGGGATCGGGAAAAAATAC
CTCGGGACTGTAAATTGCACCATTTGGAGGTTGACCGAGCAAGCTGTAGTGAGCTGGGCA
TTTGTTCATTCGACATTTTAATAGAAGGTCGTGAATGTGTGAACTGTGGAGCCACTTCTAC
TCCACTGTGGAGAAGAGATGGGACAGGACATTATCTGTGCAATGCCTGTGGTCTGTACCA
CAAAATGAATGGGCAGAACCGACCACTCATTAAACCCAAGAGGAGATTGTCTGCTGCCA

GGCGAGCGGGCACATCATGTGCTAACTGCCAAACGACCACCACCACATTGTGGAGAAGG
AATGCCAATGGGGACCCTGTGTGCAACGCGTGTGGATTGTATTACAAACTACACAACATT
AACCGACCACTGACCATGAAGAAGGAAGGCATCCAGACGAGGAACAGGAAGATGTCCAG
CAAATCCAAAAAGGCCAAAAAATGCCACGACGGGCTGGATGAGTTCTCGAAGAGCCTGA
TGGAGAAAGCGAGCTCCTTCAGCCCTGCCACGCTCTCAAGACACATGTCTCCCATCGGGC
ACATCCCACACTTCAGCCATGCAGGCCACATGCTGTCGACACCGACACCCATGCACCCCT
CGTCCGGCCTCTCCTTCGGACCCCACCATCCCTCCAACATGGTCTCCGCCATGGGATAA

STAT6 (SINCAMT00000012771)

ATGTCTCTCTCTAACCGCCGGTGGCTGTTCCAGGACCGGTTGCCGTTCCACATCCCGGAGC
GAGTGCTGTGGGGAGCCATGTGTCGGACGCTGGACATGAAGTTCACGGCGGAGGTTCAG
GCTCCACAAGGTCTGTGCCCCGAACACTTCCTCTTCCTCGCTCAGAAGGTGTTCGACGAAC
AGACTTACAGCCAGGACTTCAGTGCCAGGATGGTGTCCTGGTCACAGTTTAACAAGGAGA
ATTTACCGGGGAGAACGTTCACTTTCTGGCAGTGGTTTGACGGGGTGATGGAGCTCACCA
AAAGACACCTGAAAGATTACTGGAACAACGGGTAG

IL-4/13A (contig_23087; Dijkstra *et al.* 2014 [1])

ATGTCGAAGGCCGTGAGCATCATGATCGTTCTGATGACAGTCTTCGCCAGAAGCGACTGC
GGAGTTCTGAGAGAGATATTATCCAAACGGCAGGCCATGGGGGAACTCATTCAAACTCTG
TTACAGTTAACTGCAGGAGAGGTTGCTTGTGGTAACACCATGGTGGCTGATGTTGTATTTG
CTGGAAACGCGGCACCTGAATCGTTCTGCAAAGCTGCTAGAGCGTTGCTGCAAGCACCTT
CGTGCAAACCCATTGAACAGCAACTGAACAACCTGATCATAAACCTGCAAATAACAGGA
GTGGAGACATCCAACTGCTCCCTTGACGAAAATTCTGAGGTTGAAATGAGCACCTTTCTG
ATAAGCCTGAAGAAATTCTGCAAAAAAGTCTTCAGCTTGCAATAA

IL-4/13B (XM_007902042; Dijkstra *et al.* 2014 [1])

ATGGCTCACATACTGAAAGCTGCAGCTCTCCTCTCTATCCTATTGAGTGCTGTGTGTGCCA
CTCAGATCACCACAAAGGCCAGACATCAGTCTCTGAAGGAGATCATCAGTACCATCCGGC
ACATTTTAGAAAACCAGGTTCATCATCATCAAAACCACGTGCCAGAGATATTCGAGGACG
TAAAGGATCGCAGTGATTCAGAGGTTTTTTGCAAAGCGGCCCAAGTCCTGGAGTTGCTTC
CATTTTACAATAACCTCAAAGAATTGCACAAACTTCGACACAACCTAATTCACATGGGAG
GGCAGATAACTGAGTGTGAAGTCAGTGAACTTTCTCAGGTCGAGTTCAAAAACTTTCTGA
AAAGACTGTTGAACTTCTCCCAGAAGAAATTTAGGACAAATGGTGACAAGAATCATTTGA
CGACAAAATCGAAGGGTTGA

IL-4/13C (contig_23087; Dijkstra *et al.* 2014 [1])

ATGAAGCCACTTACAATCGCGTTCATGTTTCTCGTCCTGACCTTCTCCGAAGGCTGCAAAT
GCAGGGCAGTGACTGAAATTATCACAGACCTGGGCTTTCTGGAAAAACACATGGCAAAA
CTGGTCCCGGTCATTTGGAATGAAAACATCAGAcCATCCAACGGGTCTGTTTCTTGCAAAT
CCCACGATGTGCTGAGAAAGACTGTGTTGAACAGAAGTGGCTTCGTAGCGAAACGTACTG

AGCAATTGATCCACAACCTTTTAGCTGTGGGCAGTGTGAATCAGACGTGCGTGCTGAACG
CGCGGTCGGAAATCGACCTGCCCGCCCTGCTGAAAAGCCTGAAGAACGAAATGAGGCGG
AGGTTGTGA

IL-4/13D (XM_007902044)

ATGACTATGATGCTGAAAATTTTGACTGTTCTCACTGCCATGCTGGCTGTCAGCTATGGAA
AGGTTGACCCCAAGAAACATATCTTAAGGGAAATCACCAAAACTCTGGGACATATGACCC
AGGACAAGATTGAATGTGGTGCTACTCTAATCCCAGATGTCTTCAGTGATACGAAGGGGC
AGAGTGCATCGGAAATCTTTTGCAAGGCTGCCGAAGCTCTGGTGAAAATACCTCCCTGCG
AGTCCATCAAACGTCACTTGAAGGCTCTGAGATTAAACTTAGCGAAGGAGGGTGAACAG
ACTCTCAGCTGCCCTGTGAATAAATTCTCTGAAATCGAGCTGACTACTTTCCTGAGGAAGT
TGAGGAAGCTCTCGCAACAGAAATACAGACAATAA

IL-5A (contig_23082; Dijkstra *et al.* 2014 [1])

ATGACCAAAATGATCTGCGTCTACGTACTTCTCTTTAACAGTTTTTTGTTTGTGTTTGGCCG
AAACACTCACCCTTGCAGCAGGCCTCAGATTTATGAAACGATCAACGCCATCAAGAAACA
TGAACAGGATATAATAAAGAAGCTGCCAGCTGAACTGGAATCACCAATCAACGTTGACG
ATGAAAATTGCAAGCAGCAAATCAACAGCACATTCTATAAAGGGTTGAAGCCACTGTACG
AGACGGATTTGAAGCCTATTGCTGAAGAGATACTTTCAAACATGACCAATGACAAAGCCG
ACGTCCAGGATAAACAAAGCGACTGCAACCAGAACGTGCAAACAGTGAACGGGGCCAAG
TTCCTGAAAAGGTTTCTGAACTATCTATTCAGCAAAATGTCCCGCATGAGCCGCAGGAGA
CGGAGCCTGAAACAAATTCTGGCTAACTTCCATTAA

IL-5B (contig_23081 and 23082; Dijkstra *et al.* 2014 [1])

ATGATCAGCTTCTATATGCTTTTGTTTGCCAGCTCTTTGTTCACATTGGGCTGCTCTAGAAA
GCCCTGCCACTATTCCACGATTTGCGAAATGTTAAAAGAATTGAAAAAAGTGGAGAAACT
CGTTCTACCAGGTAACCTGCAAGCACCAATCAATGTTGAAAATAAGCAATGCGTCCAAAC
GATCATCGGCATATTCAACAGAGGATTCAAGCAACTGAAGAAAGAGCTGCACAAACGAG
ACCCGACAACGTCGGACATTTTTTGCATAATACTTTCCAACCTGCATTATGAGGAAATGCA
CCCCAAGTTGCCTTTCCAGAACAAACATAGTTCGTGCACAGCGAGTGACATAAACGTGGA
AGAAATGAACAGCGCCAAATTTCTGAAAAAATTCAAGAACTTCTTACAAGCTTACTACAA
CACTCTGTTGAACCAGAATAAAGAAAGGAAACGTTGA

Golden Mahseer

TH1-Signature Genes

T-bet

atgggcggcATAGGCAGGAATTTGTATCTCAATATGTTGAATGGAACAGACGCGCAAAGTTTC
GCCAAAGACGCAGAACTGAACAGCCACCTGCACCGAAACCGCGACTTACCGGACTTCAA
AATGGGCATCCAAGACACGAGGTTTTACTACCAGGACTCGATTCCAAATGGACAGGACAG

TTTGACGTTGCCGTTCCATGCTGAACACCACCAAAGTGCCGGAGCACAGACGGGGAGGTT
TTACGCACCGACTCCGCTCGGCAGCTGTCACTTCACCAGCCGGAGCGGGACGGGACATAC
TTACGTTCCAGCCGGAGCCGACGCGTATTCTTCAGCCAACGGTAAAGACGTGTACCCGAG
CAGCGCAGACGGCTACCCGACGTCTTTTCAGCACGGGTATCCGCGAGCACCGATGTACTC
TTTACCTGGTCTACAGGTGTCTGGGAAGACTCAAGTGCTCCTCAACAACTACCCGCTGTGG
GCAAAGTTCCACAAGTACCAGACAGAGATGATCATAACGAAACAAGGACGGAGGATGTT
CCCTTTCCTCAGCTTTAACATCAGCTCACTGGACCCCTCGGCTCATTATAACATATACGTG
GACGTGGTTCTTGCAGACCAGCATCACTGGAGGTACCAAGGAGGCAAATGGGTCCAGTGC
GGCAAGGCAGAAGGCAACATGCAAGGAAACAAGGTTTATGTTCACCCAGAATCCCCAAA
CACTGGAGCACACTGGATGAGGCAAGAAATATCGTTCGGAAAACTGAAGCTAACCAACA
ACAAAGGCAGTTCTAACAATGTAGCACAGATGATAGTGCTGCAGTCGCTCCATAAATACC
AACCACGTCTTCACATTGTGGAGGTGAAAGAGGATGGAACCGAGGATCCCTTTCTGACCT
CGAAAACTCAGACGTTTGTCTTCCCTGAGACTCAGTTCATTGCTGTCACTGCCTACCAGAA
TGCAGATATCACTCAGCTGAAAATAGATCATAACCCTTTTGCTAAATGTTTCCGGGACAAT
TATGACACGCTCTATGCACCTCCTGACTCAGACCGCCTTACGCCTTCCCCAACTGAGGGCC
AGCAGCTCCTCCCAGGCAGTTGTTACCCTCACCAGCCGTACCTGTCTGATCAATACATGAG
CCCCCTGTCTCAGAGTCGTTTCTACAGCCGCGAGCCGGTCAGCATGACCCAGCAGTGCCC
CAAAGACCCTGCCGCCAGCCCTCACAGCCGCTTTTACCTGCCCAGCCAGCAGGGGGTGCC
GCCAAACCGCCTCGACTTCAACGCATACGAGAGCGAATACACTTCCAACAACCTCTACAA
GCCGTTTCCGATTCAGACATCCCCACATCACCCTCTGGGCTACTACTCTGAAAGCCCCTTT
GCTTCTGGGCCGGTTTCTGGCGCCAACGCAGGAGCGTGGAGCTCAGGACGACCCTCACCG
CAGTACCTTAATCACCCACACCACAGTAAACCCGGGTCTAGTCTTGGCTGGTTCCGAACC
ATCTCGTCCCCAGCCGCGTCTTCGTCTCCCTCAGCGACAAACTCGCGCTTCCATTCCTCCC
CGTCGCTCCTGGATCCCCAGCGCCAGCCGCTGCTCCAAGACAAGTCCAAAGAGTCTGGGG
AGGACACCTGGATGGAAGCCCCCTCGGTTAAATCGGTGGACTCAGCTGACTCAGGTTTGT
TCGAGGGAGGAGAGAGCAAGAAGAGGCGCGTGTCGCCTTACACGTCCAGCACGGAAAAT
TCCCCTCCCAACCGCAGTGCAGAGGCTTGCGAAAAGGACAATTGCAGTGACGCGGGCTAC
TACGGCTTCTACACCCACTGA

<u>STAT1</u>

ATGAGTCAGTGGTTGGAGCTTCAACAACTGGATTCAAAGTTTTTGGAACAGGTCGACCAA
CTGTACGATGACAGCTTTCCAATGGCCATCCGGCAGTACCTCAGCAGCTGGATAGAAAGC
CACGACTGGGATCATGTTGCAAATGTGGAGAACGAGTCTCTGGCCACCGTGAGGTTTCAT
GATCTTTTGACCCAGTTGGATCACCAACACAGTCGCTTCGCGATGGAGAAAGACTTCCTG
AACCAGCACAACATCCGCAAGATCAAGAGGAACTTGCAGAGCCTCTTCCAAGACGATCC
GGTTTCCATGGCGATGATCATAAGTAGGAACCTTAGCGAGGAAAGGAAGATTCTGGCCGC
TGCTCAAGTGTCAGAGAATCGAACCGAAAGCACCCAAAATGACATGATCGTGGAGAAAC
ATCGAGAAATGGATGGAAGGGTGAAAGACATAAAGATGAAAGTACAGGACACAGAGCA
GACTATCAAAAATCTAGAAGACCAGCAAGACGAATACGACTTCAAGAGCAAAACTCTAC
AGAGCAGAGAGGAACTGAACGGACCATTTCAAAGGAAGACCTGAACCGTGAGAGACTG
ACTATCCGTGCCATGTTTATGAAACTCAACGACACAAGAATAAATGTGATCAGCCAGATG

TCAGAGGTGTTGAACATTATAGATCCGGTGATCTTTGAGCTCATTTCTACAGAATTAGGCG
AATGGAGGCGCCGTCAACAGATGGCTTGCATCGGTGGACTAACCAATGTCTGCTTAGACC
AGCTGCAGAACTGGTTTACTTCTCTAGGAGAATCTCTGCTTCAGCTCAGGCAGCAGCTCA
AGAAACTTCTGGAGCTGGAGCAGAAATTCACATACGACAGCGATCCAATCACCCTCAGCA
AAAGCACTCTGGACGACAGGATCATGTCCAACATCAAAACTCTCATCACGAACTCATTGG
TAGTTGAGCGACAGCCATGCATGCCCACCCAACTACAAAGACCTTTGGTGCTGAAGACGG
GTGTTCTGTTCACCCTCAAATTACGGCTGCTCATAAAACTTCAGGAATTCAACCACACTGT
TCGGGTGAAGGTGCAATTTGACAAGGATATCGTTGAGAAACGGAAAGGGTTTCGCATGTT
CAACATCTTGGGAACGGCCATGAAGGTGATGAACATGGAGGAATCCAATGGGATGGCAG
CAGAATTCCGTCATCTGCAACTGAAAGAGAAGAAAGTTACCGGAAACAGAACAAGTGAG
GGCCCTCTGATCGTCACGGAAGAACTTCACTCCATCACCTTTGAGGCTGAGCTCTGCTGGT
CTGGAATGGTCATGAACTTTGAGACTACATCTCTGCCCATAGTCGTGATCTCCAACGTCAG
TCAGCTACCCAGTGGATGGGCCTCCATCATGTGGTACAACATGCTGACCTTTGAGCCAAA
GAACCTTTCGTTCTTCGTGACTCCACCTTCAGCCACGTGGGGACATCTAGCGGAGGTGCTG
AGCTGGCAGTTCTCCTCCATCACCAAAAGAGGTCTTAATCAAGAACAGTTAAGCATGCTG
GGGACCAAACTCCTTGGTCCTAAAGCGGTGACGGATCCTGAAGCTCAGATTCCTTGGAAT
AGATTCTGCAAGTCTGGTAATGAGAAGAACTTCACCTTCTGGCTGTGGATTGAAGGAATT
TTGGACCTAATAAAGAGACACCTGCTGAGTCTGTGGGATGATGGCTGTATAATGGGCTTT
GTGACCAAGGACCGAACTAAAGCTCTGCTACATGACAAAGCTTCCGGGACGTTTCTGTTG
CGCTTCAGCGAGAGCAACCGCGATGGAGCCATCACGTTCAGCTGGGTTGAACACGACGCA
AACGAAGAGCCACAGATTCGCTCTGTGGAGCCCTACACGAAGAAGGAACTCTCTGCCGTC
TCACTTCCTgatataatacgggactataaggtcATGGCCGCCGACAACGTCCCGGAAAACCCTCTGCGCT
TCCTATATCCCAGTATACCCAAAGACGACGCCTTCAAAAAATACTACTCCAAACCTACCG
ACAAGCAAGAACCAATGGATGTGGATAAGAAGTTACATGAGGGATACATCAGCACCACT
CTCATCTCCATCTCTGAAATGGAGCCACAGGACTATAACGACACACTCATGCCAATGTCG
CCTGAGGTTTACGGCGAGCTGGAGCGCATGAACCGATATGATACATTAAATTTTGAGCCA
TCAGACTTAAGTCTCTCGTAA

STAT4

ATGAGTCAATGGAAACAAATACAGCAACTGGATATTAAGTTCCTCGAACAGGTGGACTAT
TTTTATGATGACAACTTTCCAATGGAGCTGAGACAGGTGCTGGCCAACTGGATTGAAAGC
CAGGACTGGGAAACAGCTTCAAACCATGAATCTATGGCTACGGTACTCTTCAATAACTTC
CTCATTCAGCTGGAGAGGCAGTGTTCTCAAGAGCAGAACTTTCTTCACAGACACAACCTA
AAGAGAATATTTCATCAGATACAGGTAAAATACAAGGCGAATCCTCTGCACATGGCCGCT
GTCATCTGTAACTGCCTGAGGGAAGAAAGACGCATCTTATCCACAGCCAGCATGCAGGAG
CAGGGACCACTGGAGAAGTCAATGCAAAACTCAGCCGCCTTGGAGAAGCAGAAAGTTCT
TGACAACAAAGTGGCAGTCATAAGAAGCAGCGTTCAGATGCTGGACCAGGCTGTGAAGT
ATCTTGAGGACATGCAGGACGATTTTGACTTTCGCTACAAAACACTCCAACTTCGAGATC
CAGTTGAGAGAAACTCTTTAACCATGAAGCAAGAAGTCACTACACTTCAGGAAATACTGA
ACCGCCTGGATTTCAAAAGAAAGGAGATTTTGTCAAAGATTGCAGATGTTATAAAGGAAA
TTGATACCCTGATCTCCTCCCAGCTCAACCCTGAGCTTAAGGAATGGAAACGGAGGCAAC

AGATCGCCTGCATCGGAGGACCGGTTCTGATAGGACTGGACCAGCTCCAGAACTGGTTTA
CCCTGACAGCCCAGAGTCTTTTCCAGATTAAAAGACAGCTAGACAAACTGGGTGAGCTGA
TCCTGAAAGTCACTTATGAGAGTGATCCAATCCCTCTCCAGAGACCTCAGATGGAGGATC
AAGTCAAATGCCTCATCTACCACCTCATTAAAAGGTCCTTTGTGGTGGAGAAGCAGCCAT
GCATGCCTACACACCCTCAGAAACCTCTAATCATCAAAACACAGGTGCAGTTTACCACCA
AGGTCAGATTACTGGTTAAGTTACCTGAGGTGGATTATCAACTCAAAGTCAAGACCACAT
TTGATAAAGACCTTCCACCAGGCAAAGTGAACAGACAGTTCTTCATCCTTACCAACAACA
CCAAAGTGATGGATGTTGAGGAGTCGACTGGATGTCTTTCTGTAGAGTTCAGGCATCTGC
AGCTGAAAGAAAGAAAGTGTCCTACTGGAGGAAAAGGGAATGAGGGTCCCCTGTCAGTA
ACAGAGGAGTTACACTCTCTCAATTTTGAGGCCATGCTGATGTTACAGGGCCTGGATATT
GATCTTGAGACTTGCTCCCTGCCACTGGTGGTCATCTCCAATGTCAGTCAGCTCCCAGGGG
GCTGGGCCTCCGTCATGTGGTACAACCTGCTAACTGACGACCCCAAGAACCTTGGGTTCTT
CAGTAACCCCTTGCGGGCAAGCTGGAGTCAACTCTCAGAGGTACTCAGCTGGCAGTTCTC
AAGTTTTGCAGGCCGAGGACTCAACAAAGAGCAACTTAACATGCTGGGAGATAAACTTCT
GGGTCAACACGCTTCTTATAATGACTGTCAAGTTTCTTGGTCCAAGTTCTGGAAGGAAAA
CATTCCTGGCAAATCATTTAGTTTTTGGCTGTGGCTGGACTCGATCCTGGACCTTATCAAG
AAGCATTTACTTCCTGTTTGGATTGATGGCTACATAATGGGATTTGTGAGTAAAGAGATG
GAACGTGCCTTACTGAAGGAGAAGGAGCCAGGGACATTCCTGCTACGCTTTAGTGAAAGT
CACCTTGGAGGAATCACCTTCACCTGGGTTGAACAGGATGAAAATGGAGACCCAAAGTTC
ATCTCTGTAGAGCCGTACACGAAAAACCGTCTCAACGCCTTGCCCATTGCTGATATAATA
CGGGACTATAAGGTCATTGCAGATGGTGTTGTCCCAGAGAATCCTCTAAAGTTCCTTTATC
CTGACATCCCGAAGGATGAGGCTTTTGGGAAACATTACAACAGCCAGCAAAACAAAGTTT
GTCCTTACATTCAAACACAGTTAGTTCCAATTTCCCATCGGAATGGTCCTGTCCAAAATGC
ATGTTCCTCTCCTGAACCCCCCATGTCTCCTGGAATGTTTGAAATTATCACCCAGCACCTC
AGCCCCTTTGAGTTTGAAAGCGCTATGAGTTCACCCTAG

IFNγ

ATGATTGCGCAACACACGATGGCCTTTTTCTGGGGAGTATGTTTGTTGACTTTAGGATGGA
TGACATATGGCGAGGCCAGCGTCCCTGAGAACCTGGACAAGAGCATCGACGAGCTTAAA
GTGTACTATATAAAAGATGACAGAGAGCTACACAATGCACATCCTGTCTTCCTACGGGTC
CTAAAAGACATAAAGGTGAATCTTGAGGAACCTGAGCAGAATCTTTTGATGAGTATCATA
ATGGATACATACAGCAGGATATTCACTCGCATGGAGAAGGATAGTCAGGATGAAGCTAC
AAAAGAAAAACTGGAACATGTTCAAGAGCATTTGAAAAAACTGCAAGAAAACTACTTCC
CAGGCAAAAGTGCAGAGCTCAAGACGTATGCAGAAACCCTATGGGCGATCAAGGAAGAT
GACCCAGTCGTCCAGCGCAAAGCATTGTTCGAGCTGAAGCGTGTCTACAGAGAAGCAACA
CAGCTGAGAAACCTGAAGAACAAAGAGCGCAGGAGACGACAAGCCAAAATCACAAAAA
GCAAAAGTCTTAATAGATCTTGA

TH17-Signature Genes

IL-17A/F1

ATGTCATCAGCGCTAAACATCCAGTTCCTCATGGTGGCTTGTATGATGGGGCTTGTTTTAA
TATCATTTGTAGCTGAGGGGGCACCTTCAAAACATCTTCCGAAGAAAGGAAACCACAGGT
CATCAGAAGAATCGGATCCATCATCTTCATCATACCGGTTGATCTTGGATCATGAGTTCAA
ATCCTCGGCCAGCTCGATCCACCCAATCAACAACGACTCCATCTCTCCATGGACCTACAC
GTTTACACATGATGACAGCCTGTACCCTTCTAGCATCGCAGAGGCGAAATGTTTGCTGACT
GGATGTTTGCTAGAAGGCGCTGAGAATTTCCATTCCAAACCGATTTACACCCAAATCATG
GTCTTGAGGAAAATTCGAGGAGAAAAGCAGAACTACTCCTTAAAACTTGAGTACAAAAC
CATCGCAGTGGGATGCACCTGCGTTCGTCCATATGTACAACAAGCTTAA

IL-17A/F2

ATGTTTCTGAGCTTCTTCAATGCTAAAAACATGGTTTTGCTGAGCTGTGCGCTGGCGAACA
TCACATTTGCCAAACAAGGGCAGAAAAAAATATGTGACACTAGTTTGAGGATTGACTTTC
AAGTCCATCTGGATGCAGACGCAGAGGGAAATGGCAACATCCACACCCGCTCACTATCTT
CATGGACCTGGGTACCAAAGTTTTCACCACACAGAATACCACAGGTCATCTTTGAAGCCC
AATGCAATTCAGAATATTGCACCCTTTCCAATGGCGTGGATACGAGACTGAACTCATTGC
CCATTTATCAGGAGATCCTGGTGCTGAAACAGGACACGGAGGACAGGAAGTGTTTTAGGG
CGACTTTTGAGAGAGTAATAGTGGGCTGTACGTGTGTGTGGGCCAAAACCTACTAA

IL-17A/F3

ATGCAGCTCTCATTGGTTTTCAGAGCTGTGCTGCTGCTTTACCTGCTTGCCCTGTTACTAAA
TGGCGCTTCTTCTGAGAAAAAGTCTATAAGGCGGTTTATCAGCGGCATCTTCAGATGTGAT
TCTGAGGGATGCAATAGCTTTTGCCAAAAGAAAGAAAAGTGGGTCATCTTGGATCCATCTC
TGAACCTCTCAATGAATGTTCCCCCATCTCCAAGCAGATCTTTGTCACCATGGACATACGA
AGAATCCTATGACGATTCACGATTCCCATCCAAAATCTCTGAGGCAAAATGTGAAAAAACA
GGCTGTTTAACCAAAGATGGTGAGGAAGACTTGGGACTGGAATCTAAACCCATCTACTACC
AGATCAACGTCTTGAAGAGAGTGAAGGAAAAAAATGCTATGCACTACACCCTTCAGCTGG
AGACTAAAGTAATTAGTTTGGGCTGTACATGCGTTCTACCCATCGTTGTGCCTCAGAATTAG

IL-21

ATGAAGGCGTCTGTGTGTTTTGTGTTTGCAGTGGTTTGTTGGATCGCAGCGCAGGCAGAG
GAGTCGCCAAAGATCCTCACGCTCAGTAAAGTTATGAATGAACTCAACAAGATCAACCGG
GGAATGAAGATAACGGACAAAAGCACAAGTTTGAACTCACCCACCGTCAACGATCTGAA
GGACTTCTGTATTGCATCTGCTCTGGAATGTTTTCGGTCCCAAGTGATTCACCTGTCAGTC
ACTGATGCCAAACTAAAGCGGTCACAGAAGATCATCTCACATGAGCTTCGCAAATCAGTC
ATTGTGAACAGTGTGTCCAGTTGCAAACCTGAAGAGATACAGGCAGCTCAGTGCAAATCA
TGTGGCTCGTACAAAAAGGTTGACAGCCGAATGTTCGTAAAGAATTTTCAAACTCTTCTG
CAAAAGATCTATGCCAGTCAAGCATAG

IL-22

ATGAAGTTCCTAACTCTGCTTGCTGTGATGTTCTGCTGCTGCTTTCTGCGCGGCCAGGCGA
TGCACCTCTCTCGCCCGCGCCCCAAGCCTTTAGACAGCTCCGTCACCTGGAACGACCTCTT
CACGATGACGGAACATGCACAGAAGGAAGATACAGACCACGAAACAAGACTGCTCCCAG
TGATCTCCGAGGTTATGCTGAAAGAGGAGAATACTTGTTGCGTCAATGCCATGATCCTGA
ATTACTATCTAAAACACATCTTGCACGCAGATGAGCATGTTGATAAGAAATACCCACATA
TTCGTTTTGTGAGGTCTGACCTGCAGAGGATCGCAAACGTTCTGAAGCCACACTGTGAAA
GCGTCGACTTTGCAGACCATGTGCATGTTAAGGAATTCGAGAAGAACTATAAAACTGCCA
GTGAACATGAATATGAGAAAACTCGTAACAAAGCAGTTGGAGAGACGATTATTCTCTTCC
ACTACCTCTTTGAATCCTGCAGTGCAAGAGTATGA

T_{reg}-Signature Genes

FoxP3

ATGCTTCAAAATGGTGCTGGAACACACAGAGGTGGAGATAACTGGAGTAGTCACCAAAA
TCAGTACCAAGAGGAAGACAGTTCTACCTTTTCCAGCATACAGACCCAGGCCAGAGGAAG
CAGTTCATCTCTGATCTCAGCCAAACCCATGGCTACCAAGGTATCTGTTGGTATCTTGGAC
TGTGATTTCAGAGGTTCTGGCTCATCTCACATTCAAAATTCCTCTCTACAACAGCAGACTG
CCAGTGACAATACAAGCAAACATTTCTTTCAGCTCAGACCATCAGTACTACGCAAAGGCA
ACCAGCCGTTCCCACAAGCTTGTGGTGTTCAGGACTGGGTTGTGGACACTGTGTGTAAGA
TAGAACCTGACAGTGACCTGTCAGAGCCTATGCCACTTTACACCAGCCAAACTGAAAGCA
GACTCAGTGCCTCCATGTCAAGTCCTAAGCCTGGCAGCACAGAGCATACTGGGAAGCATG
CATACAGTGTTCTAGGTGGCTTTCTATGCGTTAAAGGACAATGCGGATGGCCAGGCTGTG
ACAAAGTCTTTGAAGTCTATACTCATTTTCTGAAACATCTAAACCGAGACCACCATGCTCC
TGGGGACAGAGGTATAGCCCAGTTGAGGATGCAGAAAGACAAAGTACAATACATGGAGA
ATCAGTTGACTGCAGAGAGACAGAAACTGCAGGCTATGCAGCTACACCTGTTTGATGTCA
AATCTACCTGTGAGGATGGTAACACTCTGGAGAAGTCGGGACATCTGTCAGAACTCCTGC
AACCTGCAGCATATCAAAACGCAAATGGTGTCTGTGACAGTGACAGAGCGGCAGCTGAA
GCGCTAACACAAGGATACAGGCAGATCTCTACCTCACAAGTTATTCCAGGTCATCCTGCA
CAATATGCTTCGCTAATACGACAGGCAATTTTGGAGTCTCCTGAGAAGCAGCTAACACTA
AATGAAATCTACAACTGGTTCACTCGAACATTTGCGTATTTCAGACGCAATGCAGCAACC
TGGAAGAATGCAGTTCGCCACAACCTCAGTCTTCATAAGTGCTTTGTGCGCGTTGAAGGA
AGGAAAGGTTCTGTTTGGACCGTGGATGAAGAGGAATTTCTGAGAAGAAAAGGACAAAA
GTTTCACAGGGATCAGGATATGGGCTGGATGGCGCCTTTTCACGTGTTTCCTGTGACTCCA
CAAGGTGAAACTTATCAGATACGAGGCCCAAATGGACTCTCATGA

IL-10

ATGGTTTTCACTGGAGTCATCCTTTCTGCTCTGACCATGCTTCTGCTTTCTGACAGTGCTCA
GTGCAGAAGAGTCGACTGCAAGTCTGACTGTTGCTCATTTGTGGAGGGCTTTCCTGTGAG
ACTGAAGGAGCTCCGTTCTGCATACAGAGAAATACAGAGGTTTTATGAGTCCAATGATGA

CTTAGAACCGTTACTGAACGAAAACGTGCAACACAACATCAATAGTCCTTATGGATGTCA
TGTCATGAACGAGATCCTGCGCTTCTACTTGGACACCATTCTGCCAACAGCTGTCCGGAA
GGACCACTTGCACTCCAAAACACCAATTGACTCCATTGGAAATATATTTCAGGATCTCAA
GCGGGATATACTCAAATGTAGGAATTACTTTTCTTGCCAGAATCCCTTTGAGTTCGCCAGC
ATAAAGAACTCGTATGAAAAGATGAAGGAAAAGGGCGTTTCTAAAGCCATGGGAGAGCT
TGATATGTTCTTTAAGTACATCGAGCAGTATTTGGCATCAAAGAGAGTCAAGCACTAA

<u>TGFβ1</u>

ATGAGGTTGGTTTGCTTGGTACTGACGGCCCTGTGCATGGTGGCTGGAACAGGAGGTATG
TCCACGTGCAAAACTCTGGACCTGGAGGTGGTGAAGAAGAAACGGATAGAGGCCATCCG
CGGTCAGATCCTCAGCAAACTGCGCATGGCCAAGGAGCCCGAATCGGAGAAAGACGATG
ATGGACAGAAGATCCCAGATATCATGCTTTCTCTGTACAATAGCAGTGTTGAACTGAGTG
AAGAAATGAAGATGAAGCCCGTTTCTGTGCAGGCTGAAGATGAAGATTACTTTGGCAAGG
AGGTGCACAAGTTCGTCATCCGGCAAGCTCAGAATGGCACGAAGTATCAAATGACTTTTA
ACGTGTCAGAGATGAAGCAGAGTATTCCAGACCAACGGCTGCTGTCTCAAGCCGAGTTAC
GGCTTCGGATTAAGAATCCTACAATGGACCAGGAGCAGAGGCTGGAGCTGTATCGTGGA
ACAGGCGATCAGGCTCGATATCTGGGCACCCGCTTTGTCTCCAAGGACTTGTCCAATCGTT
GGCTCTCGTTTGACGTGAAACAGACGATGATAGAATGGCTGCAGGGTTCAGAGGAGGAA
GAAAGTTTAGAGCTGAGGTTATACTGTGGCTGTAAGACAGACCAGCAGAGAACTGATAA
ATTCCTCTTCAGTATATCAGGACTAGACAAGCAGAGAGGTGACACTGCCGGTCTGGCAGA
GATGATGGCGAAGCCTTATATTCTGGCTTTATCTTTGCCTTCGAATGGCAACTCCCTCGCA
TCCGCTCGCAGGAAACGAGCTGTTGGCACTGATGAAACTTGTGATGAAAAAACTGAGACC
TGCTGTATGCGGAAGCTTTACATTGACTTTCGGAAAGATCTGGGTTGGAAGTGGATCCAC
AAGCCGAAGGGATACTTTGCCAACTACTGCATGGGCTCCTGTACCTACATCTGGAACGCT
GAGAACAAATACTCTCAGATATTAGCCTTGTACAAACACCACAACCCAGGTGCATCTGCT
AAGCCATGCTGTGTACCTGCAGTCCTAGCCCCTCTGCCGATTCTGTACTATGTGGGCAGAC
AACACAAGGTGGAGCAGATGACCAACATGGTGGTGAGGAGCTGCAAGTGCAGATAG

T_H2-Signature Genes

<u>GATA3</u>

ATGGAAGTAAGTCCGGAGCAGCCCCGTTGGATGACCCACCATCACTCGGTCATTAACGGC
CAGCATCCCGAGACACACCACCCAGGACTGGGACACTCCTACATGGACCCGTCGCAGTAT
CCCCTCGCGGAAGATGTGGATGTATTGTTTAATATCGACGGACAGAGCAACCACCCGTAC
TACGGGAACTCGGTTCGGGCTGTGCAGAGGTACCCGCCACCTCACGGTACTCAGGTGTGT
CGCCCATCATTGCTGCACGGCTCTCTTCCTTGGCTCGATGGAAGTAAATCTATCGGCCCTC
ACCACAGTACCTCCCCCTGGAACCTCGGGCCTTTCCCCAAGACTTCCCTGCATCACAGTTC
CCCTGGGCCTCTGTCCGTCTACCCCCCAGCCTCCTCCTCCTCGCAGGCCGCTGGCCACTCC
AGCCCACACCTCTTCACCTTTCCCCCCACTCCACCCAAAGATGTGTCCCCTGACCCCGCAA
TTTCCACGTCAGGCTCGGGCTCATCCGTAAGGCAGGAGGACAAAGAGTGCATTAAGTACC
AGGTGTCTCTGGCCGAAAGCATGAAGCTGGATTCGGCTCATAGTCGGAGCATGGCCTCTA

TCGGACCGGGCGCGTCCTCCGCGCACCACCCCATCGCCACTTACCCATCCTATGTGTCCGA
TTACGGGCCGGGGCTCTTCCCACCAAGTAGCCTGATAGGTGGGTCCTCTTCTAGTTATGGT
TCCAAAACGAGACCAAAAACAAGGTCCTGTTCAGAGGGTAGAGAATGTGTTAACTGCGG
GGCCACCTCGACTCCTCTGTGGCGGAGGGATGGCACCGGTCACTATTTGTGTAACGCCTG
CGGACTGTACCACAAGATGAACGGACAGAACCGACCCCTCATCAAGCCCAAGCGGAGGC
TGTCTGCTGCCAGACGAGCAGGGACCTCATGCGCAAACTGTCAAACGACCACGACGACAC
TGTGGCGGAGAAATGCGAACGGCGATCCCGTCTGCAATGCCTGCGGCCTCTATTACAAAT
TACACAATATCAACCGACCACTCACCATGAAGAAAGAGGGCATCCAGACCCGGAACAGG
AAGATGTCCAGCAAGTCCAAGAAGAGCAAAAAGTCCCATGATAGCATGGAGGATTTCTC
CAAAAGCTTGATGGAAAAGAACAGTTCCTTCAGCCCAGCCGCCCTGTCGCGTCACATGAC
CTCGTTCCCTCCCTTCTCGCACTCTGGCCACATGTTGACCACGCCCACCCCCATGCACCCC
TCCTCCAGCTTGCCCTTCGCCCCTCACCACCCTTCCAGTATGGTGACGGCGATGGGCTAG

STAT6

ATGGCGCTGTGGGCGCAAATGACCCCGCAGTTACAGTACCTGCCAACTCAGACGCTGAAT
GAACTCTACCCAGACACATTCCCTCTGGACGTCAGGCACTATCTGGCCAGCTGGATAGAG
GAACAGAGATGGGAGAACTTTGATGTGGACAACATGGAGCAGGAGCCTCAGGCCCGAAG
GCTGTTGGATCAGATGGTGCATTTGCTGCAGGAGGTGGCCCAACACAACCCTAACATGGT
GGAGAGGGTCAAACTACAACATATCAGCCGGAACTTGAGTGTGTTCCAGTCACAACCCAT
GCAACTTGTGAGGACAGTTCGAGATATTCTAAGGAAAGAAAGGCACTTATTAGCACAGGC
ACAGGGCATGCACCCTAACTTCCCACCCTTCCAGCAAATTCCTCAGGGTCCTCCATCTACC
AATGGCACCCCACAACCTGCAAGGGAGGATGGGAAGTCTGATGTGGACAATCTGGTGCTC
AAAGTTTTGGAAATTCAGAACTGCCGCCAAACACTTCATCAGCTTCAAGAGGATTTGAAC
TGGGAAAAGCAGGAAATGGAGTCAATTCAAAGTCAAAATGAGTTGGATCCACAGCATTC
TGATCAAGTCGCTAGACAGACTCGCATACAGCAGCTTGAGTACAACTATTCAGTCTGTGC
AAAGAAGCGCGTGCAGCTCCTTCACGAGGCCGTGTGCGCTCTGGAGCAGTGCCAGTCCAG
CCTTATTCTCAGAATTAAGTCCTGGAGGTGGGAACAGCACCGAGCAACCATTGGCGTGCA
CTTCGATGACAACCTTGGCCCTCTTCAGACATGGTGTGAGCAGTTGCTTGGAGTAAATGGT
AACCTGAGGCAGGAGCTCATGTTGGTGACGAGAGACCACGGAGGGGCTGAGCAGTTTCA
CACGTTGCTGGAAAGCCTGGCACATCTGCTCCAGACTCTCATACAGAGTTCATTGGTGGT
AGATAAGCAGCCTCCCCAGGTCATCAAAACCCAGTCCAAGTTTTCCACCACTGTACGCTA
TCTGCTGGGAGAGAAGATGGCCCCTGGGAAACCTGTCCTGTTGAAGGCTCAGATTATTAC
TGAGGCTCAAGCGAGACACCTGGGTCAACAAGGTGTCGTACCCAATGAAAATGTAGGAG
AACTTATCAACAGCACGGCCATCTTAGAGCACAACACTTCAAGCAAGAGCACATGTGCTA
CCTTTAGAAACATGTCCATAAGGAAGATCAAACGAGCTGACAGGAAGGGCTCTGAGTCA
GTGACGGAGGAAAAGTTCGCTTTGTTGTTTACCACAGAAATGACCATCACTGGCTGTGAG
AGCCCTTATAGCATTCAGGCAATCTCTCTTCCTGTGGTTGTAATTGTACATGGTAGTCAGG
AAATCAATGCTATGGCGACAGTACTGTGGGATAATGCTTTCTCTGAGCCAGGTCGAGTGC
CATTTGTCGTTCCGGAGCGTGTGCCGTGGAAGCAGATGTGCGAAGCTCTTAGCAGTAAGT
TCATGTCTGAAGTGCAGACGCAGCGGTGCTTGGACAGATACAACCTTCACTTTCTCGCCC
AGAAGATCTTCGACCAACCAGACATCTCTGAAGATTTCAGCAATATGCTAGTGTCCTGGG

CTCAGTTCAACAAGGAAGTTCTGCCTGGTCGAAACTTCACCTTCTGGCAGTGGTTTGAAG
GGGTGCTGGACCTCACCAAGAAGTGTCTGAGAGACTATTGGAGTGAAGGACTGATCTTTG
GCTTCATTGGAAAACAGCATCTCCATGTCATCCTGCAAAACAAACCCAACGGCACCTTTC
TTTTGCGCTTTAGTGACTCAGAGATTGGTGGCATCACCATCGCCTACGTGGGTCCCAGTGA
TAATGGCGGCAGGAAAATCCAGAATATCCAGCCCTTCACCAAGAAGGATCTGGACTCAGC
TGGCCTTGGTGACCGCATACGGGACATTAACTGCATCACTCATGTGTATCCAGACTTACCA
AAGAATGAAGTATTTAAGAAATTCTTTACAGTGCCTTCTCCCCCTAATCCTGATGGATACC
TGCCATTCAAACTAACAACAGTAGTCAACCCAGGAGACCAGTGTTCCTCAACCACCAGCA
TACCACCTGACACTCCTATGGATATGTACAGCCAATCTATGAGCAATGTCTATGGGTCAA
ACATGTGCGTATTTGATTAA

<u>IL-4/13A</u>

ATGATGAAGACTATACTTCTGCTCGCTTTTGCTGTATTCGTCTACAGTGTTCCTACGGAAC
TCAGCGTCCCTGAAAAACACAGTGATAAGACTTTATTGGGAGAGCTTGTTTTGAGCTGG
AAAAAGCAGTGAAAATTTTCTCAAAGGAGACTGAGGCAAACCCAGTCTCCCTGACAGAA
GTGCAAATGAATGGCTGTAAGCGTGAGGTTTTCTGCCAGGCTGAGCAAGAACTGATAAAG
AAGGTTTCTGGGCTGTCTGGTGCCAGATTTGACCGTTTTCGTATTGACATGAAACTAATGA
GGAATTTAAACACGTATAACAAGCACCATGTGAAAACCTGCAAACCCGCTGATGAAGGC
CAAGATGAAATCCTTCTGCATGATTTCTTGAAAAATTTCTTGACATGTGTTAAGAGTGCAT
ATAGCCAAGCCAAGTAA

<u>IL-4/13B</u>

ATGAGAACTTTAATGCTCTTGGTGCTGACAATTGTAGCTGTTACTGGATCAAGACACCCTA
ACGCGGGAGAAATTCTCCTAGTGGAGATCATGAAGGACGTGGACGTGATCCTAAACCAA
AGTTCAAAGACGAATCTCAGCGATTTTGTAAAAGACGTATTTCCACAAGCGGGCTGCTCG
GAGAAACACCTTTGCCAAGCAGCAATGGTTATGATGAACACAAATCTACACTTCTCCAAG
CTACACAGACAACTGCTTGCCTACATTAATTATTCAGGGCACCATCAGTGCAGCGTTCCA
GCTTCAGAGGAACACAGAATGCGTGTTTTTCTGACAAAAATCAAAGAATGCAGCCAAGA
ACAATACTCTAAACTCTTAAAGAATAAAAGACACGTGAAATGA

<u>IL-5fam?A</u>

ATGAAACTGCTAAGTCTTCTCGTCGCACTGCTAGCTCTCAGCTATGCAAACTCAGACATGA
CCGTTAAAACCTTTTCTGAACTCCTGCACAGGCTAGAAAGACTTGAGAAGCATCAGTTCG
ATGCACTCAGTATATCAAAACCGATCCAAACACCTCGAGATATAAAGAATGCATGTGAGT
GCAAGGACTTATTTGCAGAATGCCTGAAGCACTTTCTGAAGTCAGTTACAGGGGTACCCA
AAATCCAGAGGGCCAACATTTTGGACAACCTGAAGCCTTTCCCTCATCCTGGAGTCAACG
CCAATGACAAGTGTAACTTCACAACGCATAACCTGACAAGATTGACATCAGAAAATTTTA
AGAAGTTGATTCAGGATAACATTGAATTCATCCAAAACTGGAATGCACACTGCAAACCAA
ATAATGCACGTCACAAAAGTAACTAA

<u>IL-5fam?B</u>

ATGAAACTGCTAAGTCTTCTCGTCACACTTCTTGCTCTTAGCTATGCGAACTCGAACACCG
CTACGGTCTTCTCTGAACTGCTGCACAGTCTTGAAAAACTTGAGAAGCATCAGTTCAATGC
ACTCAATAAATCAGTACGAATCCAAACACCTCAAGAAGTAAAGAACGCATGTGATTGTGA
GGACTCAATTGCAGGACACCTGAGGCACTTTCTGCGGTCATTTAAAATTGTAATACCCAG
AGCCCAGTTAGCCAACATTTTTGACAACCTGGATTACTTCAATCTTAGAATCGAGCCGAAT
GACAACTGTAGCTTCAAAACGCATAACCTGACCAGTCTGACAACAGACAATTTCAAGAAG
TCCATTCAGAATTACACTGAGTTCATCCAAAACTGGAATGCACACTGCAAATAA

Northern Pike

T_H1-Signature Genes

<u>T-bet (XM_010866213)</u>

ATGGGCGGCATAGGTGGCAATCTTTACCTCAGTATGTTGAATGGGAATGATACGCAAACT
TTCGGAAAAGACATCGATATCCACAATCCCATTCAACGCAGCAAGGATTTAGCGGAGTTT
AAAATGGGAATTCAGGACACCAGGTTCTATTACCAAGACTCAATTCAAAATGGACAAGAC
GCGTTGGCACTGCCGTACCACGCAGATCAAACTGTGGCCGGATTCGGAGCGCAACCCGGG
AGGTTTTACTCAACTCCCCTAAATAGCTGCCATTTCAGCAGTGTCAGGTCGCCGACTCGAA
CTGGGACGGGACAGAATTATTTAACACCTGGAGAAGAAGGTTTCTCAACAACTAGCAAA
GAAGCTTACTGTGGCTCTCCGGAGAGTTATTCCACGCCCTTTCAACACGGGTACCCTCGCG
CGCAACTCTACCCTTTACCTGGCCTACAGGTGTGCGGTAAAACGCAGGCAATCCTCAACA
ACTATCCTCTGTGGGCAAAGTTCCACAAATACCAAACCGAAATGATCATAACGAAACAGG
GGCGGAGGATGTTCCCCTTTCTGAGTTTCAACATCAGTGTTCTAGACCCGTCGGTACACTA
CAATGTATATGTGGATGTGGTCCTGGCCGACCAGCACCACTGGAGGTACCAGGGAGGGA
AGTGGGTCCAGTGTGGTAAAGCGGAAGGCAACATGCCAGGGAACAGGACGTACATGCAC
CCTGACTCCCCCAACACAGGGGCCCATTGGATGAGGCAGGAGGTTTCGTTCGGCAAGCTG
AAGCTCACCAACAACAAGGGCAGCTCCAACAACGTGGCACAGATGATAGTGCTGCAGTC
GCTTCATAAGTACCAGCCACGTCTCCACATTGTGGAGGTGAAGGAGGATGGTTCTGAAGA
CCCGTTCCTCACTGCCAAGGCCCAGACCTTTGTCTTCCCAGAGACCCAGTTCATCGCTGTC
ACAGCCTATCAGAACGCAGACATCACTCAGCTGAAAATAGACCACAACCCTTTTGCAAAA
GGTTTCCGGGACAATTATGACACGCTGTACGCACAGACTGACTCAGACCGCCTCACCCCC
TCGCCTTCCGATGGCCAGCAGCTCCTGGCGGGGCCCTGCTACACCCAGGGGTACCTGACG
GATCAGTACATGAACTCCCTGCCCCAGGGCCGCTTCTACAGCCGAGAGCACATGGCCGCC
ATGAGTCAGCCGCCCAAAGACCCCTCAGCCACCCCCCACGGCCGCTGGTACCTCCCCCCC
CAGCAGGGTGTCGCCTCCAACCGACTGGAGTTCAGCTCTTACGACGGCGACTTCTCCAGT
CCCGGCTTCTACAAGCCCTTCCCGCTGCAGTCGTCAGCCCACCACGCGCTGGGCTACTAC
GGGGACAGCCCCTTTGCCTCAGCCAGCGCCGGGCCCCCGTCGACAGGCTGGAACACCGCC
CGGCCCACCCCCCAGTACCTCAACCACCCCCACCCCGGTAAAGCCGGTCACGGCCTGGGC
TGGTTCAGGCCGGTGTCCTCCGCCTCGTCCTGCTCCACCTCCCCGGGTCACCCCAGACTCC
ACGCCCCGTCCCTCCTGGAGCCGCTGCGCCCCGCGCCCCCACCACCCGACAAGCCCAAGG

ACGTGGGGGCGGAGGACCCCTGGCTGGAACCCCCCTCGGTTAAATCGGTGGACTCGGCCG
ACTCTGGCCTGTATGAGGGCGTGGCGGAGAGCAAGAAGCGACGGGTGTCGCCGTACGCC
TCCAGCACGGAGAACTCGCCGCCCACGCGCGGCACAGAGGTGTGCGAGAAAGACGGTGG
CGGTGACGCTGGCTACTACGGCTTTTACACCCACTGA

STAT1 (XM_010866213)

ATGGCCCAGTGGTGCCAGCTGCAACAACTGGATTCTAAATACCTGGAGCAGGTTGATCAG
CTGTATGATGACTCCTTCCCAATGGACATCAGGCAGTACCTGAGCAAGTGGATTGAGAGC
ATCGATTGGGAAAATGTGGCTGTCCAGGACTCCTTGTCAACAGTACGTTTCCATGACCTCC
TAGCTCAGCTGGATGACCAGCACAGCCGCTTTGCCCTGGAAAACAACTTTCTGCTGCAAC
ACAACATCCGCAAGATCAAGAGAAACCTCCAGGACCGCTTTCAAGAGGACCCCATTCACA
TGGCCATGATCATTGCCAGAAACCTGAAGGAAGAGCAAAGGATTCTGGCTGTTGCCAGAA
GCATACAGGCCGACTTGGAGAACACACAGACGAGCATGGTCCTGGAGAAACAGAAGCTG
GATAATAAAGTTAAAGTGATGAAAAACGAAGTTCAGGTCGTAGATCATAACATAAAGCTT
CTTGAAGAACTTCAAGACGAGCATGACTTTAGAGTGAATAGTCTTAAGAACAGAGAACAT
GAAAATAATGGGCTGACCCCAAAACAGCTGGAACATGAGAAGATGATTATTCTTGAAAT
GTGTTTAAAGCTGAAGATTAATAGAGAGGAAGTGGTGTCTCAGTTAGCAGATGTGCTTAA
CATGTCTGAGTCAATCCAGTCAGACCTGATCTCAGAGGAACTACCTGAGTGGAAGAAAAG
GCAACAGAGCTCCTGTATTGGAGGGCCACCCAATGCCTGTCTGGACCAATTACAGAACTG
GTTCACTGTGGTGGCTGAGAACCTGCAGCAAGTGAGGCAGCAGCTGAAGAAACTCCAAG
AACTAGAGCAGAAGTATACATACGAGAATGATCCAATTGGACTGAAGAAAAGCTTCCTG
GAGGGGCGGGCCCTCACACTGTTCCGGAACCTTCTAGAGCATTCCTTGGTGGTAGAGAGA
CAGCCTTGTATGCCCACACACCCACAGAGACCCTTGGTGCTGAAGACACAGGTTCAGTTC
ACTGTTAAGCTCCGATTCCTCGTGAAGCTCCAGGAATTCAACTATCAACTCAAAGTCAAA
GCTTCGTTTGACAAAGATGTTACAGAAAAAAAAGGTTTTCGTAAATTCAACTTGTTGGGT
ACCAACACAAAGGTTATGAATATGGAAGAGTCCAATGGAAGTCTGGCTGCAGAGTTTCGC
CATTTGCAACTGAAAGAACAGAAAGTGGCCGGAAACAGGACAAATGAGGGGCCTCTTAT
TGTGACTGAGGAGTTGCACACCCTCTGCTTTGAGTCTGAGCTCAACCAGTCCGGCCTGGA
CATCAAGCTAGAGACCATCTCCCTGCCGGTCGTGGTTATCTCCAACGTCAGCCAGCTGCCC
AGTGGCTGGGCCTCTATTCTGTGGTACAACATGCTGACCACTGAGCCCAAGAATTTGAAG
TTCTTCCTTAGTCCTCCTGCTGCCACCTGGGGTCAGTTGTCTGAGGTTCTGAGCTGGCAGT
TCTCCTCTGTCACTAAGAGGGGACTCAACGAGGAACAGCTGGGAATGCTGGCTGACAAAC
TGCTAGGCCATAAAGCGCAGAGGAATTCAGAGGGCCTTATCCCCTGGACCAAGTTCTGTA
AGAGTCTCACCGAGAAGAGCTTTCCCTTCTGGCTGTGGATCGAAGCCATTCTGGACCTTAT
TAAGAGACACCTGCTTTCCCTTTGGAATGATGGTTGCATCCTGGGCTTTGTGAGTAAGGAG
AGGGAGAAGGCCATGTTGACCGGGAGGTGTCCAGGCACGTTCCTGCTGCGTTTCAGTGAG
AGCAGCAGAGAGGGTGCGATCACATTCACCTGGGTGGAGCACGACCTATATGGTGATTAT
CTTGACAAGCCCGTGTTCCATGCGGTGGAACCATACACCAAAAAGGAGCTGACCGCCGTC
TCTCTGCCTGACATAATCCGCACCTACAAGGTCATGGCAGCTGAGAACATCCCGGAGAAC
CCGCTCCGGTTCCTCTACCCGGACATCCCCAAGGACAAGGCTTTCGGGAAGTACTACGCC
AGGCCCACAGAGGTGTCAGAACCCATGGACGTGGAGAGTCCAGCCAGCCGAGGATACAT

GAAGACAGAACTCATATCAGTGTCAGAAGTTCACCCATCTAGACTGCAAGACAACATGAT
GCCGATGTCACCAGATGTGTTTGGTGAGCTGACGAGGAGTGTTGGTCCAGTGTTGGACCA
GTGTTGGAATCAAAGCCCTAAGGATTTGGATGCTGTGGCGTGTGATTTCGCTGATTTGATG
CCTGAAGACTTTCAGATGACTGCTGACTTGGACTCCCACTGA

STAT4 (XM_010890773)

ATGAGCCAATGGAAACAAATTCAACAGCTCGAGATCAAATACCTAGAGCAGGTGGACTA
CCTTTACGATGACAACTTCCCTATGGACATTCGGCAGGTGCTCTCCAGCTGGATTGAGAGT
CAAGACTGGGATACTGCCGCAAACCATGAGTCCATGGCAACAGTTCTCCTCAACAATCTC
CTATCTCAATTGGAAAGGCAATGTTCTCAGGAACAGAACTTCCTGCAAAGACACAACCTG
AAGATCATCAACCAGCAGTTACAGCTGAAGTACAAAGCCAGTCCTCTGCACATGGCCGGG
GTCATCGCCACCTGTCTCAGGGAGGAGCGTCGTATTATCTCTACTGCCAGCATTCAGGAA
CAGGGCCCATTGGAGAAGTCCATGCAGAATCCAGTGGCCTTTGAGAGACAAAAGAATTTG
GATAACAGGGTGGCTGTGATCAGGAGTAGAGTGCAGATGATGGACCAGGCGGTTAAACA
TCTGGAGGACATGCAAGACGACTTTGACTTCCGCTACAAAACTCTTCAGTCTCGTGATCCG
GCAGACAGGAACACCGACGTGATGAAACAGGAAGTGACTCGACTGCAGGAGATGCTCAA
CAACCTGGACTTTAAAAGGAAGGACAGTCTGTCTAAGATGAGGGACGTGATAAAGGAGA
TTGATGCTCTGGTAGTCTCCCAGCTCAACCCAGAGCTAATGGAGTGGAAACGCAAGCAGC
AGATTAACTGTATAGGCGGTCCGATGCTCACTGGATTGGAACAACTCCAGAACTGGTTTA
CTCTGACAGCCCAAAGCCTGTTCCAGATGAAACGTCAACTGGACAAAATGGGGGAGCTTA
TCCTGAAGGTGACCTATGAGAGCGACCCCATCCCCCTGCAGAGACCGCAGATGGAAGAG
CAGGTTAAATACCTACTCTACCACCTCATCAGAAGCTCCTTTGTGGTGGAAAAGCAGCCTT
GTATGCCAACCCACCCCCAGAAGCCTCTCATTATCAAGACTGGAGTGCAGTTCACCACCA
AAGTCAGATTGCTAGTTAAACTCCCAGAAGTGGATTATCAACTGAAAGTGAAGACAATAT
TTGACAAGGACCTTCCCCCTGGCAGAGTAAATCGTCAGTTTTCATCCTGACTACTAACAC
CAAAGTAATGGACGTGGAGGAATCATCCAATGGCTGTCTATCTGTGGAATTCAGACACTT
GCAGCTGAAAGAGAAGAAATATGTGAATGGAACCAAGGTGAACGAGGGTCCTCTCTCCG
TGACGGAGGAGCTCCACTCCCTCAGCTTCGAAGCCCAGTTCAACATACTGGGCATCAATA
TTGACCTGGAGACATGCTCCTTTCCTCTTGTCGTGATATCCAATGTCAGCCAACTGCCTGG
AGGATGGGCCTCTGTAATGTGGTACAATCTGCTAACGGATGAACCCAAGAACTTGGGCTT
CTTTGCCAATCCCCCGCGAGCCAGTTGGGGCCAGCTCTCTGAGGTATTGAGTTGGCAGTTC
TCCACCTTCGCTGGACGGGGCCTCAACAGAGAGCAGCTGCACATGTTGGGAGAGAAGCTA
TTGGGTCACCAAGCTTCCCACAGTGAATCTCAGGTGTCCTGGTCAAAGTTCTGCAAGGAA
AACATACCTGGGAAGCCTTTCAGTTTCTGGATGTGGTTGGATTCCATCCTAGAGCTCATCA
AGAAGCACCTGCTTCCCATCTGGAACGACAACTGCATCATGGGATTTGTAAGTAAGGAAA
CCGAGAGATCCCTGCTGAAGGATAGGGAGCCTGGGACCTTCCTGCTACGCTTCAGTGAGA
GCCATCTTGGCGGGATCACGTTCACGTGGGTGGAGCAGTCAGAAAATGCAGGAGAAGCC
AAGTTAATCTCGGTGGAGCCATACACAAAATCCCGTCTCAGTGCCCTCCCATTCGCTGAC
ATCATCCGTGACTACAAGGTTTTCGCAAATGGGGAAGCCCCGGAGAACCCCCTCAAGTTC
CTCTACCCTGACATTCCCAAAAATGAGGCCTTTGGCCGGCATTACAACAGTCAGCAGAGC
AAAGTCTTTCCATACATACCATCTATGCTGATCCCCATCTCTACTTTGCATGCCACACCTC

CCCCCTGCTCCTCCCCAGAGCCTCCCATGTCCCCTGGGATGTTTGATATGCTCACCCAGCA
CCTTAGCCCCTTGGAGATTGAGACTGCTATGAGTTCTCCATACTCTGAGTAG

IFNγ (XM_010882688)

ATGGATATGTTGTTAAGGGCTGTGCTGTGTGTCTGCCTGCTGGACACGATGACTTCAGGAT
GGGGCTGTGAGGCCCAGCACACATCAGACAACATGAAGACACACATAGAGAGGCTACGG
AACCACTTTAAGATCTCCAAGGACCAACTGTACAATGGACACCCTGTTTTCGCCAAGGAA
ACCTTTCAGAACAAGTATGAGGACAATGAGCGGCGAGTGTTGATGAGTGTGGTTCTGGAG
GTATACCTCAGTCTCTTCACCCATATGCTGAACCAGACAGCAGACCAGGAACCGAAGGAT
AGTCTGATATATGTCCAGAAAAAGGTTCAGGACCTCCAAAAGCACTACTTCCTGGGGAAT
ATACCTGAGCTCAAAAGACTAATGCAGGATCTGTGGGCCATCAAGACGAGTGACAGCTTA
ATTCAAGGGAAGGCCCTCTCCCAGTTGAGGACCATGTACGAGGAGGCCTCCAAACTGGCC
AATCACATCTACCTGAGAAAGTCCAACCGCAGGAAGCGACGGCAGGCCCTGAGGCACCA
AGCTATGGCGGGCTGA

TH17-Signature Genes

IL-17A/F1 (GATF01011446)

ATGGGTTTAATTTTAAACTTACACTCTCTAATGGCTCTTTGTACGATGGGGGTGTTGGTGA
TGATGATGAATATGGCACAGGCATTACCCCACCCCAACCCAGAGAAAATACTGAGCAACC
CTCGGGCTCACAAGAAGTCTGCAGAGCCATCCGATTCAGACACAATAACTGTCCCCCTGC
ACATAGACCCTAAAGGCTTGGTTCCTTCCCGTCCGATCAGGCCTATTGGGAACCATTCAAT
CTCCCCATGGACTTACCACACAACGCATGACGAGAGTCGTTTTCCCAGTGTCATCTCTGAG
GTCAAGTGCACTCTACAGGGCTGTCTGAGCATCACGGGTAAAGAGGACAGGAGCCTTGA
GTCCAAACCCATCTACCACCAGATCCTGGTTTTAAGGAAGGTGGTGGGGAGCGTGGACAG
ATACCATTACCGGCTGGAGTCAAAGATTATTGCGGTGGGCTGCACTTGTGTCAGGCCTGT
CATCGTACACCAACAGCAGGAGTAG

IL-17A/F2 (GATF01034854)

ATGTCGAAGTATCTGGTTCTGTGTTTTGTGTCTCTGCTGCTGAGCCTGACTGTCTCAGATGT
GAAAGCGGGGACAAAGGAATGGTGTAAGGACACACTGACAATCCCTTCAGACTACAACA
GCCCTCCCGAGGAAGAATCAAAGGGAAATGGAAATATCCACACACACTCGCTGTCCTCCT
GGACCTGGAAGACCACTAGGATGGAAAACCGTATTCCGGAGACCATCTGGGAAGCCGTG
TGCAGCTCTATGTATTGTGTCTACCCCACCAACAGCAGCCAGGGAGTAGGCTACAGGCAGA
ACTCTGTACCTATCTACCAGCAAATCTTGGTGCTTCACACCTCAGCCACCAGCAAATGTTAC
CGAGCCTCCTTTCTAAATGTGGCTGTGGGGTGCACCTGTATCTGGGCAAGGACCTATTGA

IL-17A/F3 (XM_010883130)

ATGCTTCTGATCGCGTTCTTCAGAGGTTTGCTGGTGCTGGGTCTGGTCACATCATTGCATG
GACTGAAGAAAGGTTCGAAGAACCGAGCGAGGCTGGGGAAAGGCTCCGGCCCAGAGGAC

ACCACGTTTCTCTATCTGGATTCTAAAATAGCCACCCCCACCCTCTCAGTGTCCTCTGTAG
CCAACAGGTCCCTGTCTCCATGGACATTCAGGAACTCCCATGTTAAGAACCGGTTTCCTGA
ATACATTGCCCAGGCTCAGTGCCAGATGTCTGGCTGTCTAACGCCTGACGGAAGGGAGGA
CTTGTCCATGGAGTCCAAACCCATTCTGTATCAGATCCTGGTGCTACACAGAGTTGAGAA
AGACGATGGCCGCAAAGAGAAGAGGAAGAAAGGGAATGTGAAGAAGAACAGGAAGGGG
AAGAGATATTTCTACACACTGAAAACAGAGATTGTTACAGTGGGCTGTACCTGTGTGAGG
CCAATCCAGCTACCTCATTACTAG

IL-21-1 (XM_010893846)

ATGCTGAGTGGACAGAAAACGGACTTAATTCTAGCCGTTTTGCTCCTCTTCTTCAGCGCCA
CGAATATGAAACGGGCTGGACCATACAGCCGCGAAACTATCCTGACACTGAAGTACATAT
TAGATAAACCCCAAGAGATGGAATCGCTTGAGTGCAGTCTTTACACCCCGACGCTGGATG
AATACACGAAATGCCCCATGTCTACCTTCAGTTGCTATGTAAATGAGGTGAAAGTCTTGG
TGTCAGAGACTGGGAGGTCCCCTATAAAACGACTAGCCGAACAACTGATCCAACTGAACA
GCACAAGTAAACAACAGAAGGAAAGTGATGCTTGTCCCTGCTGTGAGTCCTACAGTGAAC
AGCCCCCAAAAGTCTTCTTATCTACATTAAGAATTATTCTGGAGAAAAAAAATACAGCTG
TCGAGCAGAAACCACGAGGGACACGTAATGGATAG

IL-21-2 (XM_010893845)

ATGCTGAGTGGACAGAAAACGGACTTAATTCTAGCCGTTTTGCTCCTCTTCTTCAGCGCCA
CGAATATGAAACGGGCTGGACCATACAGCCGCGAAACTATCCTGACACTGAAGTACATAT
TAGATAAACCCCAAGAGATGGAATCGCTTGAGTGCAGTCTTTACACCCCGACGCTGGATG
AATACACGCAGAAATGCCCCATGTCTACCTTCAGTTGCTATGTAAATGAGGTGAAAGTCT
TGGTGTCAGAGACTGGGAGGTCCCCTATAAAACGACTAGCCGAACAACTGATCCAACTGA
ACAGCACAAGTAAACAACAGAAGGAAAGTGATGCTTGTCCCTGCTGTGAGTCCTACAGTG
AACAGCCCCCAAAAGTCTTCTTATCTACATTAAGAATTATTCTGGAGAAAAAAAATACAG
CTGTCGAGCAGAAACCACGAGGGACACGTAATGGATAG

IL-22 (GATF01012417)

ATGAAGTGCACCATGATCAAACTGCTTACCGCCGCGGTAGTGATGGTGAGCATGTGTTTG
CTGCGGGATGCGTGTGCGCTCTCCATCCACATTCCACTCAGCGCCCCTTTGCAGAACGCGG
ACACCGACGCCCTGGTACAACAGGTGGCTCGACATGCACAAAGTTCAGACACAGATACA
AACGTTAAACTGATGCCAGACGTCGATACCAAAAAGAACCATCGTGACATCTGCTGCCTC
CACGCCAACATCCTGGATTTCTACCTGAGTAACATCCTGATCTCAGATAATCACAAGCATC
CTAAACTCCCTGAGCTGAAAGAACACCTGGCGCGAGTTAGCAAAGACCTAAAGGACCAT
GGCTGTGCTGTCAAACACTACAGCAATGATCGCCACAACATCGCTTTCCGCAAGAAGTTG
TCCGAGATGGGAGGAGGGAAAGGTATAAGGAAAGCTATTGGAGAGATCGATATCCTGTT
CACTTATCTGAAAGATTTCTGTGTCCAGGTCTAA

T_{reg}-Signature Genes

FoxP3-1 (XM_010875638)

ATGGAACAGACTGAGAGTGACAGGCTGAAGAGGGGGCGGCTGAACAGTGGGCGACAGC
AGCAGCAACAGCGCGAGCGGCGGGGTGAAGACATTGAGGCAGCAGACCCACGTACCCAG
CCCAGTGATTCTGTCCCCCCTCAGCTCCACACACGCAACTCTGTTACGCAGATGGGCTTCC
CACTGACAACGAGACATGGAGGTCCCCTCGTGCCCTCCTCATCTCAGCTCCAGGGAATAC
TTTTACAACAGTGTTCCAGTGAGGGGGAATTGAGACCATTTCTGCAACGGGTGTCTCGGC
GTCCCCAGTTGAGCCAGCCCAGACCCTCGGTTCTACGTCAGGGTCTCCAGGCTGCCCAGG
CTGGCTATGTACGACACCATGCACCATATACAGAGGCCAGTTCTGCTCCACCCACCACAC
TGTGTAAGGTGGAGGTGGACACAGGGATTCAGGGATCGTCTTCACCCCCTCACCCTGGGC
TCTCTCCTGAGCCCACTCGTCACCCCTCTCCCCTTAGGGGAACCAGTCCTAAGCAAAGCTC
CATCATCACTAGGCATCACAACAGACAGCTCCACTCTGACCTTGACCATGGAGGCTACAG
CATTGCGCAGTGGCGTGCTCAACAAGACATTGTGCAGTTCTTGGAGAACCAGCTGACGGT
GGAAAAAGAGAAACTCCTTGCAATGCAACTCCATCTGCACCTATCCGAGCACAAGTTGAC
TGACTTGAAGGCATGTTCAGATTGGCCCTACAACAAGAGCCTGGCACTTGTCCTGCCCCA
AAATGCAGATGGAGCAGCACGCAACGCCACTAAGGAGCAGGAAGAGCTAGCACAGCCCG
GGTATTGGCATGCTGCCACTCACCCACACGTACTGCCAGATCTTGTCCCGAGTGTTGAGTG
TTACAAATACAACAACATCAGGCCTCCATTCACCTACGCTTGTCTAATAAGATGGTCTATA
CTGGAGTCTCCGGACAAGCAGCGCTCTCTGAATGACATCTACAACTGGTTCACCACCATG
TTCTTCTACTTCCGACACAACACGGCAACATGGAAGAACGCTGTACGTCATAATCTTAGC
CTACACAAATGTTTTGTGCGAGTGGAGGGAGGAAAGGGGGCCGTCTGGACGGTGGATGA
ACTGGAATATCAAAGGAGAAAAGGACAAAAATACCACAGGGATCATCATGTGAAATGGC
TGGCCCCCTACTCCTTATATCGTCCAGAGGGACCTTGA

FoxP3-2 (XM_010875638)

ATGGAACAGACTGAGAGTGACAGGCTGAAGAGGGGGCGGCTGAACAGTGGGCGACAGC
AGCAGCAACAGCGCGAGCGGCGGGGTGAAGACATTGAGGCAGCAGACCCACGTACCCAG
CCCAGTGATTCTGTCCCCCCTCAGCTCCACACACGCAACTCTGTTACGCAGATGGGCTTCC
CACTGACAACGAGACATGGAGGTCCCCTCGTGCCCTCCTCATCTCAGCTCCAGGGAATAC
TTTTACAACAGTGTTCCAGTGAGGGGGAATTGAGACCATTTCTGCAACGGGTGTCTCGGC
GTCCCCAGTTGAGCCAGCCCAGACCCTCGGTTCTACGTCAGGGTCTCCAGGCTGCCCAGG
CTGGCTATGTACGACACCATGCACCATATACAGAGGCCAGTTCTGCTCCACCCACCACAC
TGTGTAAGGTGGAGGTGGACACAGGGATTCAGGGATCGTCTTCACCCCCTCACCCTGGGC
TCTCTCCTGAGCCCACTCGTCACCCCTCTCCCCTTAGGGGAACCAGTCCTAAGCAAAGCTC
CATCATCACTAGGCATCACAAACAGCTCCACTCTGACCTTGACCATGGAGGCTACAGCAT
TGCGCAGTGGCGTGCTCAACAAGACATTGTGCAGTTCTTGGAGAACCAGCTGACGGTGGA
AAAAGAGAAACTCCTTGCAATGCAACTCCATCTGCACCTATCCGAGCACAAGTTGACTGA
CTTGAAGGCATGTTCAGATTGGCCCTACAACAAGAGCCTGGCACTTGTCCTGCCCCAAAA
TGCAGATGGAGCAGCACGCAACGCCACTAAGGAGCAGGAAGAGCTAGCACAGCCCGGGT
ATTGGCATGCTGCCACTCACCCACACGTACTGCCAGATCTTGTCCCGAGTGTTGAGTGTTA

CAAATACAACAACATCAGGCCTCCATTCACCTACGCTTGTCTAATAAGATGGTCTATACTG
GAGTCTCCGGACAAGCAGCGCTCTCTGAATGACATCTACAACTGGTTCACCACCATGTTCT
TCTACTTCCGACACAACACGGCAACATGGAAGAACGCTGTACGTCATAATCTTAGCCTAC
ACAAATGTTTTGTGCGAGTGGAGGGAGGAAAGGGGGCCGTCTGGACGGTGGATGAACTG
GAATATCAAAGGAGAAAAGGACAAAAATACCACAGGGATCATCATGTGAAATGGCTGGC
CCCCTACTCCTTATATCGTCCAGAGGGACCTTGA

IL-10 (XM_010874587)

ATGTCTCGCCTCTCTGTCCTCCTGTTCCTGACGCTGGTTGCTGTGTTATGGTGTGAGCATGC
CCAGAGCAGAGCTGTGACGTGCTCTAACCGCTGCTGCTCCTTCATCGAGGGCTTCCCTGTC
CGTTTAAAGGAGCTCCGCACTGCCTATTCCAATATCCGAGACTACTATGAGGCCAATGAT
GCTCTGGAGACGGCTCTGCTAGATGAAAGCCTTCTACACAACTTCATGAGCCCTTTCGGTT
GTCATGCCATAGACAGGATCCTCAAGTTCTACCTCGACACGGTTCTACCCACAGCTGTGA
ACAACAACCAAACGCATAACGACCTAAAATCTCCAATCGACTCAATCGGAAACATCTTTC
ACGAGCTGAAGAAGGAGATGATCCAATGCAGGAACTATTTCTCCTGTAAGAAACCTTTTG
ACATACAGGAGTTAATCTCTTCATATAAAAAGATGGAGGCAAATGGCCTGTATAAGGCTA
TGGGAGAGCTTGACCTGCTCTTCAACTACATTGAAGAGTACCTGGTGTCCAAGAGACGCA
AACAGTGA

TGFβ-1 (XM_010898895)

ATGAGGCCGTTGTGTTTAGCCCTGGTGTGTCTGGCAGCGCTGTGTCTGGTTGCCGGGACAG
CGAGTGGCATGTCCACCTGTAAGACTGTGGACCTCGAGCAGGTCAAGAGGAAACGTATCG
AGGCCATCAGGGGACAGATCCTGTCCAAGCTCCGGATGCCCAAGGAGCCAGAGGAGGAG
CAGTCGGGGGAGGGGAAGGAGATCCCCTCAGAACTCCTCTCTCTCTACAACAGCACGGTG
GAGCTCGGTGAAGAGCAGAAGGAGCAGGCTCAACTCAGCCTACAGGAGAAGGAGGAGG
ACTACTTCGCTAAAGTGGTCCACCAGTTCACCATGAGCGCAGATAAAAATGTCACAGAAC
ATCAGATGCTGTTCAACCTAAATGAGATGAGGTCCAACATTGGGGATTACCATCTGTTGT
CCACTGCTGAACTCCGCCTGCTCATCAAGAACCCTTCAATTGACCCAGGCTCAGAGCAGC
GTCTGGAGCTCTACAGAGGGTTCGGTGACAGCAAACGATACTTGGGATCACGCTTCATCA
CCAACGAGTTGAACAACCGGTGGCTGTCCTTTGACGTCACATCCACCCTGAAGGAATGGC
TGAAGGGGAATGAGGACGAGCAGGGCTTTGAGTTGCAGTTGTACTGTGAGTGTGGAAAA
CCTATGGAAAAGTTCCACTTCAAAATAGCTGGGTTGGAAAACAATGTGCGAGGCGACACA
GCTAAAATGGCTAGGAACATGAGGAAGCCTCACATTCTGACCATGTCCGTCCC
CCAGAACTACAGCTCCCAGCTCACATCCTCGCGCAAAAAGCGGGCCACCACTACAGATGA
TGCCTGCACAGATAAAACCGAGACCTGTTGTGTGAGAAAGCTCTACATTGACTTCCGGAA
AGATCTGGGCTGGAAGTGGATCCACAAGCCAAAGGGTTACCATGCCAATTACTGCATGGG
TTCATGCACCTACATCTGGAACGCTGAAAACAAATACTCTCAGATCCTGGCCCTGTATAA
GCACCACAACCCTGGAGCATCTGCCCAGCCCTGCTGTGTACCCCAGGCCCTAGACCCTCT
GCCAATCCTTTACTACGTTGGAAGACAACACAAGGTGGAACAGTTGTCCAATATGATCGT
AAAGAACTGTGATTGCAGCTAG

TH2-Signature Genes

GATA3 (XM_010901334)

ATGGAAGTATCCGCGGACCAACCACGATGGATGAGCCATCATCACCCCTCGGTGTTAAAC
GAACAGCATCCCGGCTCACACCATCCAGGCCTCGGCCACTCGTATATGGACCCCTCGCAG
TATCCCCTCACCGACGATGTGGATGTACTGTTTAATATCGACTCACAGGGTAACCACGTAT
CTCCATACTATGGGAACTCAGTCCGAGCCGTCCAGAGGTACCCACCTCCTCCACACAGTA
GTCAGGTGTGTCGCCCCTCTTTACTGCATGGCTCTCTGCCGTGGCTGGACGGAAGCAAAG
CCATGCAGCACCACAGCACTTCTCCGTGGAACCTGAGTCCTTTCCAGAAGAGCTCATTGC
ACCACGGTTCCCCGGCCGGTCTCTCCGTCTACCCCCCGGCGTCCTCCTCTTCCCTGTCCGG
GGGTCACTCGAGCCCGCACCTTTTCACCTTTCCCCCAACCCCTCCGAAAGACGTGTCCCCG
GATCCGGGCATCTCAACCCCAGGTTCCAGCAGCAATAGTCGCCAACAGGAAGAGAAAGA
GTGCATAAAGTACCAGGTGTCTCTGGCCGAGAGCATGAAACTGGAGTCTCACAGCCGGAG
CATGGCGTCAATCGGAGCGCAATCCTCTGCCCACCACCCCATCGCCACATACCCATCCTA
TGTCCCCGAATACGGCCCAGGCCTCTTCCCACCAAGTAGCCTGATAGAAGGTAGAGAGTG
CGTTAACTGTGGAGCTACGTCGACCCCGCTCTGGAGGCGGGATGGCACTGGTCACTATTT
GTGTAACGCCTGCGGACTCTATCACAAGATGAACGGGCAGAATCGGCCCCTCATCAAACC
CAAGCGACGACTGTCTGCTGCAAGACGGGCAGGAACGTCGTGTGCGAACTGTCAAACGA
CCACCACAACACTGTGGCGAAGAAACGCGAATGGGGACCCGGTGTGTAACGCTTGCGGT
CTATACTACAAACTGCACAATATCAACCGACCTCTCACCATGAAGAAAGAGGGCATCCAG
ACCAGGAACAGAAAGATGTCCAGCAAGTCGAAGAAGAGCAAAAAGTCCCACGACAGCAT
GGACGACTTCTCCAAGAGCCTGATGGACAAGAGCAGCTCATTCAGCCCGGCTGCTCTGTCA
CGTCACATGTCCTCTTTCCCTCCCTTCTCACACTCCGGCCACATGCTGTCCACCCCAACGCC
CATGCATCCCTCTTCCTTCGCCCCACATCCCCACTCCAGTATGGTCACTGCCATGGGCTAG

STAT6 (XM_013136358)

ATGGCACAGTGGGCGCAGATGAAACAGCTGATTCCCTATCTGCCAACGCAGGCGCTTAAC
GAGCTCTACCCAAACTCCTTCCCCATGGACGTCAGGTGCTACCTGGCCAACTGGATCGAG
GAGCAGAGATGGGAGGACTTTGATCTAGAGAAGGTAGAGCAGGAGCCACAGGCCTTGTC
TCTGTTGGACCAGATCACTTCCTACCTGTTGAGCCTTGCCCAGCAGAACCCCAACGTGGTG
GAGAGGATGAGACTGCAGCACATCTCCAGGAGCATGAGCATGATGCGCTCGCAGCCCCT
GAAGCTGGTGCTCACCGTGAGGGACGTTCTGAGGAAGGAGAGAGACCTTCTCAGTCGGA
CGTCCGCTCAGATCCCGCCCCACTTCCCCCCGTCCCCAGCCCAGGATCCCATCTCTGTGGG
CCCCTCCACCCGGGACGTGGACCTGCTGGTGCTCAAGGTGCTGGAGATCCAGAACTGCAG
ACAGCAGATCCACCAGCTGCAAGAGGAACTCAACTGGGAGAGGCAGAACTATGAGTCAA
TCCAGGGCTTTGGAACAGGCACTCTTCAGCAGGCCTCGACCAATGGAATGGACACCGGCA
TCTCAGAGGTCACCAAACTGCAGAATAACATTAAACAGCATGAGTACAATGGCCAGGTCA
TAGCCAAGAAGCGTGTTCAGTTGCTCCAGGATGCTGTATCTAGTCTGGAGAGTTGCCAGT
CTCGTCTGATCAGCCGAATCAAAGCCTGGAGGTGGGAACAGCATCAGGCTACCATAGGAC
ACCCCTTTGATGACAACCTTAGCCCTATGCAGACATGGTGTGAGCAACTTCTTGGGGTGA
ATGGGAAGTTGAGACAGGAAGTGATGCTGGCCGGCGAGCCAATCCCAGAGCTGCAAGAC

AGACTGGGACTTCTGCTGCAAACCCTAATTCAGAGCTCTCTGGTGGTAGACAAGCAGCCT
CCCCAGGTAATTAAAACCCAGTCCAAGTTCTCTACCACCGTGCGCTACCTGCTGGGGGAG
AAAGTGGCGCCGGGGAAACCAGTTCTCCTCAAGGCTCAGATTATCACTGAGGCCCAAGCA
AGAAACCTTGGACAGCTAGGCAGCGTACCCAGTGAGAATGTTGGAGAATTAATCAACAA
CACAGCTATTTTGGAACACAACACCTCCAGCAAGAGCACCTGTGCCACTTTCAGGAACAT
GTCTATAAAGAAGATAAAGAGAGCCGACAGGAAGGGGTCGGAGTCAGTGACCGAGGAG
AAGTTTGCTCTCTACTTCACCACGGAGATCAGTATCACCGGTTGTGACATCCCCTATAGGA
TACAGATGATCTCTGTACCTGTGGTGGTGATAGTCCACGGCAGTCAGGATAACAACGCCC
TAGCGACCATCATCTGGGACTGTGCCTTCTCTGAACCAGACAGGATTCCATTTGTAGTTCC
CGAGCGTGTGCCCTGGAAGCAAATGTGCATGACTCTAAACAGCAAGTTCATGTCGGAGGT
TGGGACACAGCGTTGCCTAGACAATTACAACCAACATTTCCTGGCCCAGAAGATATTTGA
CAAGCCAGACATTGCTGGAGACTACAGCAACATGCTGGTTTCCTGGGCACAGTTCAGCAA
GGAGGTGCTTCCTGGACGACCTTTCACCTTCTGGCAGTGGTTTGATGGCGTGATAGAGCTT
ACCAAGAAGCACCTGAAGAGCTACTGGAGTGACGGGTTGATTTTTGGTTTCATCGGTAAG
CAGCACCTCCACCTAATTCTGAAGGACCGACCCAATGGAACGTTCCTACTGCGCTTCAGT
GACTCAGAGATAGGAGGCATCACCATCGCTTATGTGGCCCCCTCAGAGAATGGGGGCCAG
AAGATCCAGAATATTCAGCCATCCACTAAGAGAGACTTAGAGATCCGTTCCCTTGGCGAC
CGTATTCGAGACATTGACTTCATCACACACGTGTATCCAGATCTGCCGAAGAATGATGTG
TTTCGGAAATACTATACAGATAACCCACTGCCCAGTGGAACTGGGTACCTGGCCGTCTCC
ATACAGACCAGGGTGGGAGTCAGGGAATCCCACTGTGAATCTCCTCAGCGCAGTGACACC
CCACAGGACAACTGTCCTTTCTTTGCCATGGACCAAGGGAATACATCGGCCACGTCACCA
TTCTTCCCTGTCTCTCCGCAACAGACGTTCCCGTCTAGCATCCAGCCGGTTCCGGTACCAA
TGCCGGGGATCTACACAGACCCGCTGCCCGACCCCAACAGACTCTTCTCTGGACTGGACA
TGTCCCAGGGCTCATCCACTGAGATGGAACTAGAGCATATTCTGAACACTTTTGTCGAAA
GGCCGGGTGAAGATGTGTAG

IL-4/13A (GATF01012417)

ATGAAGACTTTGATCTTTCTGCTTTCGGTTAACCTGGCGCTGGTTGTCACTGCAATTCCGTT
AACACACGGGGATTCCATCCAAGCCAACTTGGAGAAGATTATTGGCTTGGCAGAGGACTA
CGTAAGAACTCTGCCTGAGGACTTTCTGAAGACACTTGGCAAAGATTTAACACATTTGAC
TGAGACCACAAATAAATGCAAGGACTTCTTTTGTGAAGTTGAGGCAGTTTTGGCATCTCT
GGAGCATCACTTCTTTGGTAAAAGGGGAGAGATTGTCCGATCACTGGAACAGTACAACAA
ACATATGAATTGCCCCAATGCGAATCACAGCCAAGGCAACCAAGTGGAGCTGAAGAGAC
TTCTACATGACCTTAAAGGATGTGGCCAGAAAATCAACTCCAATAAGGTTTAG

IL-4/13B-1 (AZJR01088263)

ATGAAGACTGTTGCTCTCCTCTTTTTTGCAATTGCATTAGGGTACAGTGCACCTATTACTG
AACCCACTGAACACAAGTATGAGCATCGGCTACTTAAAGATATCATGGATGATGTCACGG
AACTGCTCAAAGGCCCAAAGAAACTTTTGGACATGACAGTGCCTAAGGGATTCGATAAAT
ATAAGTGCTGTAGAAACGGGCAGCCTGAAGAGTTCTGCATAGCTGAGAAGATTCTGTCTG
ACATAAACTCCACACAGAACGGGATTCCAGAAAAAATTAGGAAAATCAACAGGGTGCTG

AAACAATACAATATGTTTAACCCAACTAACTGTACTGTGACAAAGAACGAAGATGAAGA
GCAGCTAAGAGTTCTTCTTCAAGATCTCGGTTGTTGTGCTCAGGACAAATACTCCCATTGG
AATCACAAAAGAGCCAAGCGTTCTGCCTACAACAAACTTAAAAATACAACATTCTGTATA
AATCAATATTAA

IL-4/13B-2 (AZJR01044272)

ATGAAGACTGTTGCTCTCCTCTTCTTCGCATTTGAACTGGTCTACACTGCACCCACTCAGG
ACGAGCATCATTTATTGAAGAGAATCATGGACACCGTCAACGATCTACTCAAGACAGGCC
CGGAGGAGTTTTTGTCAAGTATTGTGCCGCACCAATTCGATAGAGAACGCTGCGGCCGTA
AAGGGCCTGAAGATTTCTGCATAGCTGAGAAGATTTTGACTGACATAAACTATACAAAGT
ATGGGATTCCAGAAAACAGGGAAATTCACCGGTTTCTGATACAATATAATAAGTTTCATC
CAAACAACTGCACTGTGGACAAAGATGAAGAGGAGGAACAGTTAAGAGCTCTTCTTGAA
GATCTCTACTGCTGTGCTCAAGTCAAATACTCCCGTCGGAATCATAAAAGAGCCCAGCGC
TCTGCTGATCGCAATCAATGGAACATTCACCAATGTGCAAAACAACACTTTTAG

Rainbow Trout

T_H1-Signature Genes

T-bet (NM_001195793)

ATGGGCGGCATAGGTGGCAATCTTTACCTCAGTATGTTGAATGGGACAGATACGCAAACT
TTCGGAAAAGACACCGATATCAACAATCACCTTCAACGCAGCAAGAATTCCGTGGATTTT
AAAATGGGAATTCAGGACACCAGATTATATTACCAAGACTCAATTCAAAACGGACAGGA
CGGATCGGCACTGCCGTACCACGCAGACCAAACTGTGGCCGGATTCGGAGCGCAACCCG
GGAGGTTTTACTCACCAACTCCCCACAATAGCTGCCATTTCAGCAGTGTCAGGTCGCCGA
CTAGAAACGGAACGGGACAGACTTATTTACCAACGGGAAGCGAAGGTTTCTCGACAACT
AGCAAAGAAGTTTACCCTGCCTCTGCGGAGAGTTATCCCGCACCCGTTCAACATGGGTAC
TATCGCGCGCCACTCTACCCATTACCTGGGCTACAGGTGTGCGGAAAGACGCAGGCTCTC
CTCAACAACTATCCTCTGTGGGCGAAGTTCCACAAATACCAAACCGAAGTGATCATAACG
AAACAGGGGCGGAGGATGTTCCCCTTCCTGAGTTTCAACATCAGCGTTCTGGACCCGTCA
GCCCACTACAATGTGTATGTGGACGTGGTCCTATCCGACCAGCACCACTGGAGGTACCAG
GGAGGCAAGTGGGTCCAGTGTGGCAAAGCAGAGGGTAACATGCCAGGGAACAGGATGTA
TATGCACCCAGACTCTCCCAACACAGGGGCTCACTGGATGAGGCAGGAGGTATCGTTTGG
CAAGCTCAAGCTCACCAACAACAAGGGCAGTGCCAACAACGTGGGCCAGATGATCGTTCT
GCAGTCACTTCATAAGTACCAGCCACGGTTGCACATCGTCGAAGTGAAGGAGGATGGTTC
TGAGGACCTCTTCCTCACCGCCAAGGCCCAGACCTTCATCTTCCCAGAGACCCAATTCATC
GCCGTCACAGCCTATCAGAACGCAGACATCACCCAGCTAAAAATAGACCATAACCCCTTT
GCGAAAGGTTTCCGGGACAATTATGACGCGCTGTACGCACCACCTGACTCAGACCGCGTC
ACCCCCTCTCCCACCGAGGGCCAGCAGCTTTTGGCAGGTACCTGCTATACCCAGGGGTAC
CTGATGGACCAGTACATGAACCCACTGCCCCAGAACCGCTTCTACAGCCGGGAGCACATG
GCAGCCATGGGCCATCAGACCAAAGACCCCTCAGCCAGCCCCCACAACCGCTGGTACCGC

TCCCCCCAGCAGAGTGTCACCTCCAACCGGCTGGACTTTAACTCCTATGAGGGTGACTTCT
CCAGCAACAGCTTCTACAAGCCCTTCCCCCTGCAGACGTCAGCCCACCACACGCTGAGCT
ACTACGAGGACAGCCCGTTCGCCACGGCCAGCGTATCCGTCTCACGAGCCTCCTCGGCAG
CCTGGAGCACCGGCCGGCCCTCCCCTCAGTACCTCAACCATCACTGTTCCAGTAAAGTCG
GCCCCAGTCTAGGCTGGTTCAGGCCTGTGTCCTCTACCTCTCCAGGTCACCCCAGGCTCCA
CCCCCCGTCCCTCCTGGAGCCACTCCGCCCCCCACCTTCACTGCAGGACAAGCCCAAGGA
GGCGGGGGTGGTAGGAGAGGACCTCTGGCTGGAACCCCCCTCGGTCAAATCGGTGGACTC
TGCTGACTCCGGCCTGTTCGAGGACGGGGCAGAGGGCAAGAAGCGACGAGTGTCACCCT
ACGCTTCCAGCACGGAGAACTCGCCGCCCACCCGCAGCGTGGAGGTCTGTGAAAAGGACT
GCAGTAGTGATGCCGGCTACTACGGCTTTTATACCCACTGA

STAT1-1 (NM_001124707)

ATGGCCCAGTGGTGCCAGCTGCAACTACTGGAGTCTAAATACCTGGAGCAGGTCGACCAG
CTCTACGATGACTCCTTCCCCATGGACATCAGACAGTACCTCAGCAAATGGATTGAGAGC
ATCGACTGGGAAAATGTGGCTGTTCAGGACTCCTTAGCAACAGTACGTTTCCATGACCTC
CTAGCCCAGCTGGATGATCAACACAGCCGCTTCGCCCTGGAAAACAACTTCCTGCTACAA
CACAACATCCGCAAGATCAAGAGAAACCTCCAGGACCACTTTCAGGAGGACCCCGTTCAC
ATGGCCATGATCATCTCCAGAAACCTGAAGGAGGAGCAGAAGATCCTGGCTGCTGCCAA
AAGCATAGAGACCAACAGAGAAAACACACAGACTAGCATGGTTCTGGAGAAACAAAAAC
TGGACAACAAAGTCAAAGACATGAAAAACAAGGTTCAGGAAGCGGATCAGAATGTAAAG
TCTCTGGAATACCTTCAAGATGAGCATGACTTTAAGGAAAATACTCTTAAGAACAGAGAA
CATGAAATGAACGGGCTGACACCGAAACAGCTGGAACACGAGAAGCTGCTGATTGCAGA
GATGTGTTTGAAGCTGAAGATCAAGAGAGGGGAAGTGGTTGGCCAGTTGGCGGAGGTGC
TCAACATGGCCGAGGCGGTCCAGTCAGACCTGATCTCAGAGGAGCTGGCGGAGTGGAAG
AAGAGGCAGCAGATCTCCTGTATCGGAGGACCACCCAACGCATGTCTGGACCAACTGCAG
AACTGGTTCACAGCGGTGGCGGAAAGCCTGCAGCAGGTGAGGCAGCAGCTGAAGGAGCT
CCAGGAACTAGAGCAGAAGTACACATACGACAACGACCCAATCAAACAGCAGAAAGGCT
TCCTGGAGGGGCGGGCCCTGACACTGTTCCGGAACCTCCTAGAACATTCCTTGGTGGTGG
AGAGACAGCCCTGTATGCCCACACATCCACAGAGACCTCTGGTGCTGAAGACACAGGTGC
AGTTCACTGTCAAGCTCAGATTTCTTGTGAAGCTCCAGGAATTCAATTATCAACTCAAAGT
GAAAGCTTTGTTTGACAAGGACGTTACAGAAAAGAAAGGGTTTCGTAAGTTCAACATATT
GGGCACCAACACAAAAGTTCTGAATATGGAGGAGTCAAATGGAAGCCTGGCTGCAGAGT
TTCGCCATTTGCAACTGAAAGAGCAGAAAGTAGCCGGCAACAGAACAAATGAGGGGCCT
CTCATTGTGACAGAGGAGCTGCACTGCATCTGCTTTGAGTCTGAACTCAACCAGTCAGGT
CTGGAGCTCAAGCTAGAGACCATCTCCCTGCCCATCGTGGTCATCTCCAACGTCAGCCAG
TTGCCCAGTGGCTGGGCCTCCATCCTGTGGTACAACATGCTGACCAGCGAACCCAAGAAC
CTGAAGTTCTTCCTGAGCCCTCCTGCAGCATCCTGGGGTCAGCTGTCTGAGGTGCTGAGCT
GGCAGTTCTCCTCTGTCACCAAGAGGGGACTCAATGAAGAGCAGCTAGGCATGCTGGCCG
ACAAACTACTGGGCCAGAAAGCACAGAGGAATCCTGAGGGCCTTATCCCCTGGACCAAG
TTCTGTAAGAGTCTCAATGAGAAGAGCTTTCCCTTCTGGCTGTGGATCGAAGCTATCCTGG
ATCTCATCAAGAGACACCTGCTGTCCCTGTGGAACGATGGTTGCATCCTGGGCTTTGTGAG

TAAGGAGAGGGAGAAGGCCCTGCTGACTGGGAAGTGTCCAGGCACGTTCTTGCTGCGCTT
CAGTGAGAGTAGCAGAGACGGAGCCATCACATTCACTTGGGTGGAACACGACCAATACA
ATAAGCCTGTATTCCATGCAGTGGAGCCCTACACCAAGAAGGAACTGTCTGCCGTCTCTC
TGCCTGACATCATCCGCACCTACAAGGTGATGGCAGCTGAGAACATCCCAGAGAACCCAC
TCCGCTTCCTCTACCCAGACATCCCCAAAGACAAGTCCTTCGGAAAGTACTACGCTAGGG
CCTCCGAGGCATCGGAGCCCATGGATGTGGAGGGTTCCAGCAGCACAGGGTACATGAAG
ACAGAACTCATATCAGTGTCAGAAGTTCACCCATCTAGACTGCAAGACAACATGATGCCC
ATGTCGCCAGACGTGTTCGGAGAGCTGAAGAGGAGCGTTGCACCAGTGTTCCAGTGTTGG
ACTGGTCCTAAGGAAATTGATGCTGTGATGACACCTGACTCGGACTGCAACTGA

STAT1-2 (NM_001124746)

ATGGCCCAGTGGTGCCAGCTGCAACTACTGGAGTCTAAATACCTGGAGCAAGTCGACCAG
ATCTATGATGACTCCTTCCCCATGGACATCAGACAGTACCTCAGCAAGTGGATTGAGAGC
ATCGATTGGGAAAATGTGGCTGTTCAGGACTCCTTAGCAACAGTACGTTTCCATGACCTCC
TAGCCCAGCTGGATGACCAACACAGCCGCTTCGCCCTGGAAAACAACTTCCTGCTACAAC
ACAACATCCGCAAGATCAAGAGAAACCTCCAGGACCGCTTTCAGGAGGACCCCGTTCACA
TGGCCATGATCATCTCCAGAAACCTGAAGGAGGAGCAGAGGATTTTGGCTGCTGCCAAAA
GCATAGAGACCGACAGAGAAAACACACAGACCAGCATGGTTCTGGAGAAGCAAAAGCTG
GACAACAAAGTCAAAGACATGAAAAACAAGGTTCAGGAAGCGGATCAGAATATAAAGTC
TCTGGAATACCTTCAAGACGAGCGTGACTTTAAGGAAAATATTCTTAAGAACAGAGAACA
TGAAATTAACGGGCTGACACCCAAACAGCTGGAACACGAGAAGCTGCAGATTGTAGAGA
TGTGTTTCAAGCTGAAGTTCAAGAGAGTGGAAGTGGTTGGCCAGTTGGCGGAGGTGCTCA
ACATGGCCGAGGCGGTCCAATCAGACTTGATCTCAGAGGAGCTGCCTGAGTGGAAGAAG
AGGCAGCAGAGCTCCTGTATTGGAAGACCACCCAACGCATGTCTGGACCAACTGCAGAAC
TGGTTCACAGCGGTGGCAGAAAGCCTGCAGCAGGTGAGGCAGCAGCTGAAGGAGCTCCA
GGAACTAGAGCAGAAGTACACATACGACAACGACCCAATCAAACAGCAGAAAGGCTTCC
TGGAGGGGCGGGCCTTGGCACTGTTCCGGAACCTCCTAGAACATTCCTTGGTAGTAGAGA
GACAGCCCTGTATGCCCACACATCCACAGAGAACTCTGGTGCTGAAGACACAGGTGCAGT
TCACTGTCAAGCTCAGATTTCTTGTGAAGCTCCCGGAATTCAACTATCAACTCAAAGTGAA
AGCTTTGTTTGACAAGGACGTTACAGAAAAGAAAGGGTTTCGTAAGTTTAACATATTGGG
CACCAACACAAAAGTTATGAATATGGAGGAGTCAAATGGAAGCTTGGCTGCAGAGTTTCG
CCACTTGCAACTGAAAGAGCAGAAAGTAGCTGGCAACCGAACAAATGAGGGGCCTCTCA
TTGTGACAGAGGAGCTGCACTGCATCTGCTTTGAGTCTGAACTCAACCAGTCAGGTCTGG
AGCTCAAGCTAGAGACCATCTCCCTGCCCATCGTGGTCATCTCCAACGTCAGCCAGTTGCC
CAGTGGCTGGGCCTCCATCCTGTGGTACAACATGCTGACCAGCGAACCCAAGAACCTGAA
GTTCTTCCTGAGCCCTCCTGCAGCATCCTGGGGTCAGCTGTCTGAGGTGCTGAGCTGGCAG
TTCTCCTCTGTCACCAAGAGGGGACTCAATGAAGAGCAGCTAGGCATGCTGGCCGACAAA
CTACTGGGCCAGAAAGCACAGAGGAATCCTGAGGGCCTTATCCCCTGGACCAAGTTCTGT
AAGAGTCTCAGTGAGAAGAGCTTTCCCTTCTGGCTGTGGATCGAAGCTATCCTGGATCTC
ATCAAGAGACACCTGCTGTCCCTGTGGAACGATGGTTGCATCCTGGGCTTTGTGAGTAAG
GAGAGGGAGAAGGCCATGCTGACTGGGAAGTGTCCAGGCACGTTCCTGCTGCGCTTCAGT

GAGAGTAGCAGAGACGGAGCCATCACATTCACTTGGGTGGAACACGACCTATACGATAA
GCCTGTATTCCATGCAGTGGAGCCCTACACCAAGAAGGAGCTGTCTGCCGTCTCTCTGCCT
GACATCATCCGAACCTACAAGGTGATGGCAGCTGAGAACATCCCAGAGAACCCACTCCGT
TTCCTCTACCCAGACATCCCCAAAGACAAGTCCTTCGGGAAGTACTACACCAGGGCCTCC
GAGGCATCGGAGCCCATGGATGTGGAGGGTTCCAGCGTCACAGGATACATGAAGACAGA
ACTCATATCAGTGTCCGAAGTTCACCCGTCTAGGCTGCAAGACAACATGATGCCCATGTC
GCCAGACGTGTTCGGAGAGCTGAAGAGGAGCGTTGCACCAGTGCTCCAGTGTTGGAGTAG
TCCTAAGGATATTGATGCTGTGATGACACCTGACTCGGACTGCAACTGA

STAT4-1 (CDQ57838, FR904292)

ATGACTTGTGTCCCCGAACGTGGATATTTCTGTGATAGCGCACCAGTCCAAAGACACAGC
ATGAGTCAATGGAAACAAATTCAACAGCTCCAGATCAAATACCAGGAGCAGGTGGGCTA
CCTTTATGATGACAACTTCCCTATGGACATACGGCAGGTGCTCTCCAACTATATTGAGAGC
CAGGACTGGGATACTGCCGCAAACCATGAGTCCATGGCGACAGTTCTCCTCAGCAATCTC
CTGTCTCAGCTGGACAGGCAGTGTTCTCAGGAACAGAACTTCCTACAAAGACACAACTTG
AAGTTCATCTACCAGCAGTTACAGACTAAATGCAAAGCCAACCCTCTGCTGATGGCCGGG
GTCATTTCCACTTGCCTCAGAGAGGAGCGTCGTATCATCTCCACAGCCAGCATTCAAGAA
CAGGGCCCATTGGAGAAGTCAATGCAGAATTCGGTGGCCTTCGAGAGACAGAAGAACAT
GGACAACAGGGTTGCTGTGATCAGGAGTAGTGTGCAGATGATGGACCAGGCAGTGAAAT
ACCTAGAAGACATGCAAGACGACTTTGACTTCCGCTATAAAACTCTCCAGTCTCGAGATC
CAGTGGACAGGAACACAGCGGCGATGAAACAGGAAGTGACCCGACTGCAGGAGATGCTC
AACAACCTGGACTTTAAGAGGAAGGAGAACCTATCTAAGATGAGTGACGTGATAAAAGA
GATTGATGCTCTGATAGTCTCCCAGCTGAACCCAGAGCTGATGGAGTGGAAACGCAGGCA
GCAGATCAACTGTATAGGTGGCCCACTGCTCACTGGACTGGACCAGCTCCAGAACTGGTT
TACACTGACAGCCCAAAGTCTGTTCCAGATGAAGCGTCAGCTGGACAAACTGGGGGAACT
TATATTAAAGGTGACGTATGAGAGTGACCCCATCCCACTGCAGAGACCGCAGATGGAGG
AGCAGGTCAAATACCTCATCTACCACCTCATCAAAAGCTCCTTTGTGGTGGAGAAGCAGC
CATGTATGCCAACCCACCCCCAGAAACCTCTCATCATCAAGACCGGGGTGCAGTTCACCA
CCAAAGTCAGATTGCTGGTCAAACTTCCAGAGGTGGATTACCAACTAAAAGTGAAGACAA
TATTTGACAAGGACCTCCCCCCTGGTAGAGTGAATCGTCAGTTCTTCATCCTGACTACCAC
CACCAAAGTAATGGACGTGGAGGAATCATCCAATGGCTGCCTGTCTGTAGAGTTTCGACA
CTTGCAGCTGAAAGAGAAGAAATACGTTAATGGAACCAAGGTGAACGAGGGCAAATTCC
TTATCCCGTTGATAGAGCCTGCCGTGTTTTTCATTGAGATGTGTTCAACTTTTTGGTGTGTC
CCTCACTGCACAAAAAATACCTGCTCCTTTCCTCTGGTGGTCATCTCCAATGTTAGCCAAC
TGCCTGGAGGTTGGGCATCTGTCATGTGGTACAACCTGCTGACTGACGAACCCAGGAACC
TGGGCTTCTTTGCCAGTCCTCAGCGGGCCAGTTGGGGCCAGCTCTCTGAGGTACTGAGTTG
GCAGTTCTCCACCTTCGCTGGGCGGGGCCTCAACAGGGAACAGCTGTGCATGCTAGGAGA
GAAACTCTTGGGTCAGCAAGCCTCCCAAAACGAATGTCAGGTATCCTGGTCCAAATTCTG
CAAGGAGAATATTCATGGGAAGAATTTCAGTTTCTGGATGTGGCTGGATTCCATCCTGGA
GCTCATCAAGAAGCACCTACTTCCCATCTGGAACGACAACTGCATCATGGGTTTTGTCAGT
AAGGAGACTGAGAGGGCCCGTCTGAAGGACAAGGAGCCTGGGACTTTCCTGCTGCGCTTC

AGCGAGAGCCACCTGGGCGGGATCACCTTCACCTGGGTGGAGCAGTCCGATAGTGGAGA
GGCCAAGTTCATCTCGGTGGAGCCGTACACCAAATCACGCCTCAGCGCTCTCCCGTTTGCC
GACATTATCCGGGACTACAAGGTCATCGCAGACGGAGAGGTCCCTGAGAACCCACTAAA
GTTCCTCTACCCAGACATCCCCAAAGACGAGGCCTTCGGACGACTTTACAACAGCCAGCA
GAGCAAAGGCAAGATGTCTGCCAAGACATTCTTTCCATACATACCATCTATGCTGATCCC
CATCTCAACTTTGCAAACCACACCTCCCCCCTGCTCCTCCCCAGAACCCCCCATGTCCCCT
GGGATGTTTGACATGCTCACCCAGCACCTCAGCCCCTTGGAGATTGAGACTGCCATGAGT
TCTCCGTACTCCGAGTAA

STAT4-2 (CDQ57312, FR904285)

ATGAGTCAATGGAAACAAATTCAACAGCTCGAGTTCAAATACCTGGAGCAGGTGGATTAC
CTTTATGATGACAACTTCCCTATGGGGATTCGGCAGGTGCTTTCTGACTGGATTGAAAGCC
AGGACTGGGATATGGCCGCACACCATGAGTCCATGGCGACAGTTCTCCTCAGCAATCTCC
TGTCTCAGTTGGACAGGCAGTGTTCTCAGGAACAGAACTTCCTAGAAAGACACAACCTGA
AGAACATCAACAAGCAGTTACAGATAAAATACAAAGCCTACCCTCTGCTCATGGCCGGGG
TCATCTCCACTAGCCTCAGAGAGGAGCGTCGTATCATCGCCTCAGCCAACATACCGGAAC
AGGGCCCGTTGGAGAAGTCTGTACAGAATTCCGTGGCCTTCGAGAGACAGAAGAACATG
GACAACAGGGTGGTCGTGATCAGGAGTAGTGTGCAGATGCTGGACCCGGCAGTGAAATG
TCTAGAGGACATGCAAGATGACTTTGACTTACGCTTCAAAACTATCCAGTCTCGAGATCA
GGCGGACAGGAACACAGAGCGGATGGAACAGGAAGTGGCCCGACTGCAGGAGATTCTCA
ACAACCTGGACTTTAGGAGGAAGGAGATCCTGTCTAAAATGAGTGACGTGATGAAAGAG
ATTGACGCTCTGATAGCCTCCCAGCTGAACCCAGAGCTGATGGAGTGGAAACGCAGGCAG
CAGATCAACTGTATAGGTGGCCCACTGCTCACTGGACTGGACCAGCTCCAGAACTGGTTT
ACTCTGACGGCCCAGAGTCTGTTCCAGATGAAACGTCAGCTGGACAAACTGGGAGAACTT
ATCCTGAAGGTGACCTATACGAGCGACCCCATCTCGTTGCAGAGACCGCAGCTGGAGGAG
CAGGTCAAATACCTCATCGACCACCTCATCAAAAGCTCATTTGTGGTGGAGAAGCAGCCA
TGGATGCCAACCCACCCCCAGAAACCTCTCATCATTAAGACCGGGGTGCAGTTCACCACC
AAAGTCAGATTGCTGGTCAAACTCCCAGAGGTGGATTATCAACTGAAAGTGAAGACGATA
TTTGACAAGCACCTCCCCCCTGGCAGAGTACATCGTCAGTTCTTCATCCTGACTTCCACCA
CCAAAGTAATGGACGTGGAGGAATCATCTAATGGCTGTCTATCTGTAGAGTTCAGACACT
TGCAGCTGAAAGAGAAGAAATATGTCAACGGTATAAAGGTGAACGAGGGTCCTCTCTCTG
TGACGGAGGAGCTCCACTCCCTCAGCTTCGAGGCCCAGTTCAACATACAGGGCATTAACA
TCGACCTGGAGACCTGCTCTTTTCCTCTGGTGGTCATCTCCAATGTTAGCCAGCTGACTGG
AGGCTGGGCCTCCATCATGTGGTACAACATGCTGACTGACGAACCCAGGAACCCGGACTT
CTTTGCCAGTCCTCCGCGGGCCAGTTGGGGCCAGCTCTCTGAAGTGCTGAGTTGGCAGTTC
TCGACTTTCACTGGGCGGGGCCTCAACAGGGAGCAGCTGTGCATGCTGGGAGAGAAACTC
ATGGGTCAGCAAGCCTCTCACAATGAATGTCAAGTATCCTGGTCCAAATTCTGCAAGGAG
AATATTCCTGGGAAGCCTTTCAGTTTTTGGATGTGGCTGGATTTCATCCTGGAGCTCATCA
AGAAGCACCTGCTTCCCATCTGGAACGACAACTGCATCATGGGCTTTGTCAGTAAGGAGA
CAGAGAGGGCCTGTTTGAAGGACAAGGAGCCTGGGACATTCCTGCTGCGCTTCAGTGAGA
GCCACCTGGGTGGGATCACTTTCACCTGGGTGAAGCAGTCCGACAGTGGAGAGGCTAAAT

TAATCTCAGTGGAGCCGTACACCAAATCACGCCTCAGCGCTCTCCCGTTCGCCAACATCAT
CCGGGACTACAAGGTCATCGCAGACGGAGAGGTCCCTGAGAACCCGCTCAAGTTCCTCTA
CCCCGGCATCCCCAAAGACGAGTCCTTCAGACGACATTACAACAGTCAGCAGAGCAAAG
GCAAGATGGTTACCAAGACACGTTTATGA

IFNγ-1 (NM_001160503)

ATGGATGTGTTATCAAGGGCTGTGATGTGTTTCTGCTTGATGGGCTGGATGACTTTAGGAT
GGAGTAATGCTGCTCAGTTCACATCAATTAACATGAAGAGAAACATAGACAAACTGAAAG
TCCACTATAAGATCTCCAAGGACCAGCTGTTCAACGGAAACCCTGTTTTCCCCAAGGACAC
GTTTGAGGACAGTGATCGGAGGGTGTTGATGAGTGTGGTTCTGGACGTGTATCTGAGTATC
TTCGGCCAGATGCTGAACCAGACGGGGGACCAGGAAATGATTGAGAGTCTGAAATATGTC
AAGGGGAAAATTCAGGATCTCCAGAAACACTATTTCCTGGGGAGGATACCTGAGCTGAGG
ACACACCTGCAGAACCTGTGGGCCATCGAGACCAGCGACACCACAGTCCAGGGGAAGGCT
CTGTCCGAGTTCATTACCATCTACGAGAAAGCCTCCAAACTGGCCCTTAAGTTCCATCTAA
AGAAGGACAACCGCAGGAAGAGACGGCAAGCCCAGAGGCTCAAATCACACATCATGTAG

IFNγ-2 (NM_001160503)

ATGGATGTGTTATCAAGGGCTGTGATGTGTTTCTGCTTGATGGGCTGGATGACTTTAGGAT
GGAGTAATGCTGCTCAGTACACATCAATTAACATGAAGAGAAACATAGACAAACTGAAAG
TCCACTATAAGATCTCCAAGGACCAGCTGTTCAACGGAAACCCTGTTTTCCCCAAGGACAC
GTTTGAGGACAGTGAGCAGAGGGTGTTGATGAGTGTGGTTCTGGACGTGTACCTGAGTATC
TTCAGCCAGATGCTGAACCAGACGGGGGACCAGGAAGTGAGGGAGAGGCTGGACCAGGTC
AAGGGGAAGGTTCAGGAGACCCAGAAACACTATTTCCTGGGGAGGGTACCTGAGCTGAGG
ACACACCTGCAGAACCTGTGGGCCATCAAGACCAGTGACACCACAGTCCAGGGGAAGGCT
CTGTCCGAGTTCATTACCATCTACGAGAAAGCCTCCAAACTGGCCCATGAGATCCATCTAA
AGAAGGACAACCGCAGGAAGAGACGGCAAGCCCAGAGGCTCAAATCACACATCATGTAG

TH17-Signature Genes

IL-17A/F1a (KJ921977)

ATGGGTTCTACTTCAAACGTACACTTTTTGATGGTGCTGTGTTTAATGGGGTTGTTGGTGA
TGATGATGATGATGGGTGCAGAGGCTGCCCCCCACGTCCATCCAGGGTTGACAGGGAGAC
GCTCACAGAACAACCTTCAGGGACACAAGAAGCCTGTAGAGCCTTCAGCTCCAAACACA
ATAACTGTCCCCCTGCACCTGGACCCGAGCGACTTGATTCCAGCCCGTCCCGTCAGATCCA
TTGGGAACCACTCCATCTCCCCATGGACTACCAACACCACCTATGATGAGTCCCGCTTTCC
CAATACCATCTCGGAGGTCAAGTGCTCTCTGGAGGGCTGTCTGAACGTCGCGGGTAAGGA
GGACAGGAGCCTGGAGTCCAAACCCATTTACCACCAGATCCTGGTCCTGAGGAAGGTGAT
GGGGAGCAAGGACAGATACTACTACCGGCTGGAGGCAAAGATCATCGCGGTGGGCTGCA
CCTGTGTCAGGCCTAGCATTGAGCACCAACAGTAA

IL-17A/F1b (KJ921978)

ATGGTATCTACTTTAAGCTTCCAGTCTTTGACGGTGCTGTGTATGAAGGGGGTTTTGGTGA
TGATGATGGGGGCAGAGGCGGCCCCCCGTGCCCATCCAGAGAAGACAGAGAGCCACACA
CGGACCACACTGCAGACACACAAGAAGCCTGCAGAGTCAGAGTCTTCAGGTCCCGACAC
AGTAATTCTCCCCCTGCACCTAGACCCAAATTACTTGGTTCCTTTCCATTCCATCAGACCT
ATTTACAACCATTCAATCTCCCCATGGACTTACAACACTACCTATGATGATAGACGCTTTC
CCGCCATCATCTCGGAGGTCAGGTGCTCTCTGAAGGGATGTCTGAACATCAAGGGTAAGG
AGGACAGGAACCTGAAGTCCAAACCCATCTTCTACCAGATCCTGGTCCTGAGGAAGGTGA
TGGGAAGTGGGAAGAAATGCCACTACCGACTGGAGTCAAAGGTCATCTCGGTGGGCTGC
ACCTGTGTCAGGCCTACCATCGAACAGTAA

IL-17A/F2a (AJ580842)

ATGGAGCTCAAAAGCAACGTGTCGAAGTACCTGGTTGTGTGCTGTGTGTCTATGCTGCTG
GGCCTGACCATGGCGAAAGGAATGAAGGTGACAAAGGAGAGGTGTAACGAAACACTGAT
CATCCCTTCAGACTTCTACAAGATTCCCACAGAGGAATCAGAGGGAAATGGGAACATTCA
CACACGCTCTCTGTCACCCTGGACCTGGAAAAGCACTACAGTGGAGAACCGTATTCCTCA
GACCATGTGGGAGGCCGAGTGCAGCTCTATGTACTGTGTCTACCCCACCAACAGAAGCCA
GTACATGCGCTACAGGCAGAACTCTGTACCTATCTACCAGCAGGTCGTGGTACTCTACAC
TTCAGCCACCAGGAAGTGCTACAGCGCCTCCTTCCTGTCTGTGGCCGTGGGGTGCACCTGT
GCCTGGGCAAGGACTACTTGA

IL-17A/F2b (KJ921979)

ATGCACCACTCAGACAGAAGGATGTGGAAGACATCAGAGATGGAGCTCAAGAACAATGC
GTCGAAATACCTGGTTGTGTGCTGTGTGTCTATGCTGCTGGGCCTGACAATAGCCAAAGG
GGAGGTAGGGGAGAAGGAGAGGTGTGAGGACATACTGACCATTCCTTCAGACTACTACA
AGACTCCTTCAGAGGAATCAGAGGGAAACGGGAACATCAACACACGCTCCCTGTCCCCCT
GGACCTGGAAACCCATTACAGTGAAGAACCGTATTCCTCCGACCATTTGGGAGGCCAAGT
GCAGCTCTATGTACTGTGTCTACCCCACCAACGGCAGCCAGGCCGTGGGCTACCAGCAGA
ACTCTGTACCTATCTACCAGCAGGTCATGGTGCTCCATAGCTCAGCCACTAGGAAGTGCTA
CAACGTCACCTTCCTGTCCGTGGCCGTGGGGTGCACCTGTGCCTGGGCAAGAACCACTTGA

IL-17A/F3 (KJ921980)

ATGCATCTGGTCATGGTCTTCAGAGGTTTACTGGTGCTGGGTCTGATCATGTCACTGGATG
GACATAAGAAAAGCCAGAAGACCAAACGGAAGGCAGCCAATGGCTCCGGCCCAGTCGAG
GTCTTTGGACGGAGAGTAATGACTGTAGGTTTAGTTATGGATCCTAACCTGAAGACCGCT
GTCTTCCCTGTCCTTCATAAAGCCACCAGGTCTCTCTCTCCGTGGACATACAGCGACACAT
ACGATGAGACCCGGGTCCCTCAACATATCTCCCAGGCTCAGTGCCAGAGGTCTGGCTGTC
TGACCCCAGGCGGAGAAGAGGACATGGGGTTGGAGTCCAAACCCATACTCTATCAGACCT
TGGTGCTCCGCAGGGTTCAGGGAGAGAAGGGCAGCAGTGGAGGGAGGAGGAGGAAGAA

GGGGAGGAAGAACAAGGGTTATTTCTACAAACTGGACAGTGAGAGTGTAAATGTGGGCT
GTACCTGTGTGAGACCCAGCATTTTCCCTCAGAAGCAGTGA

IL-21 (NM_001246331)

ATGAAGCTGCTTGTTTGTTATCTCCTAGCCATCACCTGTGTATTGGTGGATGCAGACAAGG
GTGAGAGAATCCTGAAGCTGACTGAAGTAATAAAGGAATTAAAAGCGCTAAACAGAACTG
TGACGCACAACAGTGTGATGTCGAACGCTCCTTCAATGGATACGGAAGAGTGCTGTTCCCA
GTCTGCCTTGAAGTGCTTCCGGGCGATGGTACCTCATCTCAAGGCCAAAAATAAGACCCTT
CAGCGTAAAGTTATCAAAAACCTCAACAACCGAATGATTTGGGAAGTCTGGACTCCTGCA
GTCAGGAGGAGAGAGAGAAAACAGTCTGCCAAGGATGTGATTCATATCCAAAGGACAGCC
AGAAATGTGTACAACAGTTGGAATCTCTGCTTCAAAAGGCCATCAGTAGACTGGCGTGA

IL-22 (NM_001164064)

ATGAAGTTCAGCACGGTCCAACTCGTAGCCGCTGTGGTAGTGGTGATGAGCGTCTGTCTG
CTGCGCGAATCCGTGGCGCACTCCATCCATCGCCCGCTCAGCGCCCCCCTGCACAGCGCG
GATACCGACACCATGGTACAGCAGGTGGCTCAACATGCGCAAAGTTCTGACACCGATACT
GACACTAAACTGATGCCAGACATCGATACTAAAAAGAACCACAGAGACATCTGCTGCCTG
CATGCTAACATCCTGGACTTCTACCTGAGTAACATCCTGACCACTAAGGAGAAGCAGGAC
AAGCATCATCCTAAACTCCCTGCCCTGAAGGAAGACCTGGCCCGAGTCAGCAGAGACCTG
AAGGAACACGGCTGTGCTATTAAACACTACAATGACCACCACCACAGCATTGCTTTCCGC
AAGAAGTTGTCCGAGATGGAGGAGGGGAAAGGAATCAAGAAAGCCATTGGAGAGATCG
ACATCCTCTTCACCTTCCTGAAAGACTTCTGTGTGCACGCCTAG

T_{reg}-Signature Genes

FoxP3-1 (FM883710)

ATGCTACAGACTGAGAGTGAGAGGTTGAAGAGTGGGCGACTGAACAGTGGGCGACATCA
GCAGCAGCGAGAGCAGCGAGAGGAGCCAGACGCATGTACCCAGCCCAAAGATTCTGCCT
CACCTCAGCTCTGTGCTCGTACCTCTGTTACGCAGATGGGCTTCCCATTGATGATTAGACC
TGGAGTTTCACTTATGGCCTCGTCTCAGCTCCAAAGCATATTGTTACGGCAGTGTTCCAGT
GAGGAGGAGGGGAGATCGCTCCTGCAACGGGTGTCTCTGTGCCCCCAGTTGAGCCAGCAC
AGACCCTCTGTTCTACGCCAGGGTGTCCAGGCTGCCCACGTGCGACCGCAGGCACCATGC
ACTCTAACAGAGGCCAGTTCAGCTCAGCCCATCTCACTGTGCAAGGTGGAGGTGGACACA
GGGAGCCGTGGACAGTCCTCTCCCCCACACTCTGAGCACTCTCCTGGGCCAACTCGTCAC
CCCTCTCCCCCGAGGACAGCCAGTCCTAAGCAGAGCTCCATCATCACCAGGCAACATGAG
GCAGGTCACCCAACCCTAGAGGGGTCCAGTGCTCTGTTTCTCAACGGACTCTGCTGCTGG
CCTGGCTGCGATGCAGTATTTGAAGAATTCCCCAGTTTTTTGAAACACCTCCACTCTGACC
ATGGCCATGGAGACAGAAGCATTGCACAGTGGAAGGTACAACAAGACATGGTGCAATAC
ATGGAGACCCAGCTGACTGTGGAAAAACAGAAACTCTTCGCAATGCAACTACATCTGCAC
CTATCTGGACACAAATCAACTGTCTTGAAGGCAGCTTCAGACTGGCCCTACAGGCACAGC
CTGTCACTAGGCCTGCCCCAGAACCGAGGTGGAGTGTCACGCTGCACCACTAAAGAGCCA

GAGGAGCTGGAGCAGCATGAGTATTGGCCCTCTGCTGCTCCCCACCACCTACGGCCAGAT
CTTATCCCGAGTGTTGAGTGTTACAAATACAACAACATTAGGCCACTGTACACCTACGCCT
GCATGATAAGATGGTCTATACTGGAGTCTCCAGACAAGCAGCGCTCCCTGAATGACATCT
ACAACTGGTTCACCACCATGTTCTTCTACTTCCGACACAACACGGCCACATGGAAGAACG
CTGTCCGTCACAATCTTAGCCTCCACAAGTGTTTTGTGCGAGTGGAGGGAGGCAAGGGGG
CCGTCTGGACAGTGGATGAAATGGAATATCAAAGGAGAAAGGACAAAAATACCACAGG
GATCATCATGTGAAATGGCTGGCACCCTACTCCTTATTTCGTCCAGAGGAACCTTGA

FoxP3-2 (FM883711)

ATGCCACAGACTGAGAGTGAGAGGTTGAAGAGTGGGCGGCTGAACAGTGGTAGACAGCAG
CAGCAGCGAGATTGGCGGGGTGAACACGGAGAAGAGCCAGGCACACGTACCAAGCCAAA
TGATTCTGTCTCACCTCGGCTCTATGCTCGTACCTCTGTTACTCAGATGGGTTTCCCATTGAT
GATTGGATCTGGAGGTCCACTTATGGCCTCATCTCAGCTCCAGAACATACTGTTACAGCAG
TGTTCCAGTGAGGAAGAGGGGAGACCTTTGCTGCAGCGGGTGTTTCGGTGCCCCCAGTTGA
GCCAGCACAGACCCTCTGTCCTACGCCAGGCTGTCCTGGCTGCCCACGTACGACCACAGAC
GGCCGGTTCAGCTCAACCCATATCACCGTGCAAGGTGGAGGTGGACACAGGGAGCCGTGG
ACAGTCCTCACCCCCACACTCTGAGCACTCTCCTGGGCCCACTCGTCACCCCTCTCCCCTGA
GGAGAGCCAATCCTAAGCAGAGCTCCATCATCACCAGGCATAATGAGCCAGGTCACCCAA
CCCTACGGAGCTCCAGTGCTCTGTTTCTCAACGGACTCTGCTGCTGGCCAGGCTGCGATGC
GGTATTTGAAGAATTTCCACGTTTTTTGAAACACCTCCACTCTGACCATGGCCATGGAGAC
AGAAGCATTGCACAGTGGAGGGTACAACAAGACATGGTGCAATACATGGAGACCCAGCTG
ACTGTGGAAAAACAGAAACTCTTTGCAATGCAACTCCATCTGCACCTGTCTGAACACAAAT
CAACTGACATGAAGGCAGGTTCAGATTGGCCCTACAGTCACAGCCTGGCACTAGTCCTGCC
CCAGTACTCATCCCCGGCCTTGGCAGCAGATGGAGTGCCACGCTGCTCCACTAAAGAGCCA
GAGGAGCTGGCACAGCATGAGCAATGGCCCGCTGCCGCTCCCCACCATCTACTCCCAGATC
TTGTCCCGAGTGTTGAGTGTTACAAATACAACAACATCCGGCCTCCGTACACCTACGCCTG
CCTGATAAGATGGTCTATCCTGGAGTCTCCAGACAAGCAGCGCTCTCTGAATGACATCTAC
AACTGGTTCACCACAATGTTCTTCTACTTCCGACACAACACGGCCACATGGAAGAACGCTG
TCCGTCATAATCTTAGCTTGCACAAGTGTTTTGTGCGAGTGGAGGGAGGCAAGGGGGCTGT
CTGGACAGTGGATGAAATGGAATATCAAAGGAGAAAGGACAAAAATATCACAGGGATC
ATCATGTGAAATGGTTGGCACCCTTCTCCTTATTCCGTCCAGAAGAACCTTGA

IL-10 (AB574339)

ATGTCTCCCTGCTCTCTCCTCCTGTCCCTGCTGTTGGCTGCAGCGCTGCAGTGTGAGCATG
CCCAGTGCAGACGTGTACCGTGCTCCGACCGCTGCTGCTCCTTCGTAGAGGGCTTCCCCGT
CAGACTGAAGGAGCTCCGCACCGCCTTCTCCACCATCAGAGACTACTACGAGGCTAATGA
CGAGCTGGAGACATCCCTGCTGGACGAAGGGATTCTACACCACTTGAAGAGCCCCGTGGG
GTGTCACGCTATGGACAGCATCCTGAAGTTCTATCTCGACACGGTGCTGCCCACCGCCAT
GAACAACAGAACACAGAACAACAACGACTTTAAATCTCCCATCGACTCCATCGGAAACAT
CTTCCACGAGCTGAAGAAAGAGATCGTCCAATGCAGGAACTACTTCTCCTGTAAGAAACC
GTTTGACATCAACGAGTTCATCTCCTCGTATGAGAAGATGCAGGATAAAGGCCTGTATAA

GGCAATGGGAGAGCTGGACCTACTCTTCAACTACATCGAGGAGTACCTGGTCTCTAAGAG
ACGCAAACACTGA

TGFβ-1a (KF870471)

ATGAGGGCAGTGTGTTTGATGCTGACTGCCTTATTGATGCTGGAATATGTGTGCCGGAGT
GACACCATGTCCACCTGTAAGTCTCTGGACTTGGAGCTGGTGAAGAGGAAACGTATTGAG
GCCATCCGTGGACAGATATTGAGCAAGCTGCGGCTACCTAAGGAGCCAGAGATTGACCA
GGAGGGAGACACTGAGGAGGTCCCAGCTTCCCTGATGTCCATCTACAACAGCACAGTGGA
GCTGAGTGAGGAGCAGGTGCACACGTACATACCGTCCACTCAGGATGCAGAGGAGGAGG
CATACTTTGCAAAGGAGGTCCACAAGTTCAACATGAAACAAAGTGAGAACACCAGTAAG
CACCAGATTCTCTTCAACATGTCTGAGATGCGGTCAGTATTGGGGACTGATCGACTGCTCT
CTCAAGCTGAGCTCCGCCTCCTCATCAAGAACCATGGCCTGCTCGACGACAGCGAGCAGA
GGCTGGAACTCTACAGAGGAGTTGGCGATAAGGCACGTTACCTGAAGTCCCATTTTGTCT
CCAAGGAATGGGCCAATCGCTGGGTGTCCTTTGATGTAACACAGACTCTGAATGAGTGGC
TGCAAGGGGCTGGAGAGGAGCAGGGATTCCAATTGAAGTTGCCTTGTGATTGTGGGAAAC
CAATGGAGGAATTCCGCTTCAAAATATCAGGGATGAACAAGCTGAGGGGAGATACAGAA
ACACTAGCCATGAAAATGCCATCAAAGCCTCACATTTTACTGATGTCACTTCCTGTGGAAC
GCCACAGCCAGCTTAGCTCTCGGAAGAAACGACAAACCACTACTGAAGAGATCTGCTCAG
ATAAATCGGAGAGTTGCTGTGTGCGAAAACTTTACATTGACTTCCGTAAGGACCTGGGCT
GGAAGTGGATCCATGAACCCACTGGCTACTTTGCTAACTACTGCATCGGCCCCTGCACCT
ATATATGGAACACAGAAAACAAGTATTCCCAGGTACTGGCTCTGTATAAGCACCACAACC
CCGGAGCCTCTGCCCAACCCTGCTGTGTTCCACAGGTCTTGGAGCCCCTACCCATTATCTA
TTATGTGGGGAGACAACACAAGGTGGAGCAGTTGTCCAATATGATTGTTAAGTCCTGCAG
GTGTAGCTAG

TGFβ-1b (NM_001281366)

ATGAGACTGGTGTGTTTGGTCCTTACGTGCCTGGCGGCGCTGTGCTTGGCGGGCAGCGTG
AGCAGCATGTCCACCTGTAAGACTCTAGACCTGGAGCTGGTTAAGAAGAAACGCATCGAA
GCCATCAGGGGACAGATCCTCTCCAAGCTGAGGATGCCCAAGGAGCCGGAGGAGGAGCA
GGCGGGAGCAGGGAAGGAGATCCCTGTAGCGTTGCTCTCTCTCTACAACAGCACCGTGGA
GCTCAGTGAGGAGCAGAAACTACAGCCACAGCCCACCGCACACACACTGGATGAAGAGG
AGGAGTACTTCGCCAAGGAGGTCCACAAGTTCACCATGAAGCAGGATAAGAATGTCACC
AAGCACCAGATGCTGTTTAACCTGAATGAGATGAGGTCCAGCATTGGGGACTACCATCTA
CTCTCCAGCGCTGAGCTCCGCCTGCTCATCAAAACCACTAACATCCCGTCTGGCAGTGAG
CAGCGCCTGGAGCTCTACAAGGGGCTGGGCGACAGCAGACGATACCTGGGATCACGCTTC
GTCACCAACAAGTGGAGTGACCGCTGGCTGTCTTTCGACGTCACTGAAACCCTCAAGGAA
TGGCTGAAGGGGACCGAGGAAGAGCAGGGCTTTGAGTTGAAGCTGTACTGTGAGTGTGG
TAAACCCATGGAAAAGTTCCTCTTCAGTATCTCCGGGTTGGACAGCGGGGTACGAGGTGA
CGTGGCTGGTCTAGCCAAGAACATGAGGAAGCCTCACATCTTGGCCATGTCCATCCCCCA
GAACTACAGCTCACAGCTCACCTCCTCGCGTAAGAAACGGGCCATCGCTACAGACGACAC
CTGCACAGATAAAACAGAGAACTGTTGCGTGAGGAAACTCTACATCGACTTCAGGAAAG

ATCTGGGCTGGAAGTGGATCCACCAGCCAAAAGGTTACCATGCCAACTACTGCATGGGCT
CATGCACCTACATCTGGAATGCTGAAAACAAATACTCTCAGATCCTTGCCCTGTACAAGC
ACCACAACCCAGGAGCGTCGGCCCAGCCGTGCTGTGTACCCCAGGTCTTGGAACCTCTGC
CAATCCTCTACTACGTAGGGAGACAACACAAGGTGGAACAGTTGTCCAACATGATTGTGG
AGAGCTGTAAGTGCAGCTAG

TH2-Signature Genes

GATA3 (NM_001195792)

ATGGAAGTATCCGCGGACCAACCCCGATGGATGAGCCATCACCACCCATCGGTGCTAAAC
GAACAGCATCCCGGCTCGCACCACCCGGACCTCGGCCACTCGTACATGGACCCCTCGCAG
TATCCCCTCGCCGACGATGTGGATGTACTGTTTAATATCGACTCACAGGGCAACCACGTAT
CTCCATACTATGGGAACTCAGTCAGAGCCGTCCAGAGGTATCCACCTCCTCCACATAGTA
GTCAGGTGTGTCGCCCCTCTCTACTGCACGGCTCTCTGCCGTGGCTGGACGGAAGCAAAG
CAATGCAGCACCACAGCACTTCCCCATGGAACCTGAGTCCTTTCCAGAAGAGCTCACTGC
ACCACGGTTCCCCGGCAGGTCTCTCTGTCTACCCTTCGGCGTCCTCCTCCTCCCTGTCCGG
GGGCCACTCCAGCCCGCACCTCTTCACCTTTCCCCCCACTCCTCCGAAAGACGTGTCCCCG
GACCCAGGCATCTCCACTCCGGGCTCAAGCAGCAACAGTCGCCAACAGGAGGAGAAAGA
GTGCATAAAGTACCAGGTGTCCCTGGCCGAAAGCATGAAACTGGAGTCTCACAGCCGGA
GCATGGCATCAATCGGAGCGCAATCCTCCGCACACCATCATCACCCCATCGCCACATACCC
ATCCTATGTCCCCGAATACGGCCCAGGCCTCTTCCCACCAAGTAGCCTGATAGGTGGGTCA
TCTTCTAGTTATGGTTCCAAAACAAGACCAAAAACAAGGTCATGTTCAGAAGGTAGAGAGT
GCGTTAACTGTGGAGCTACGTCGACCCCGCTCTGGAGGCGGGATGGCACTGGTCACTATTT
GTGTAACGCCTGCGGACTCTATCACAAGATGAACGGGCAGAATCGGCCCCTCATCAAACCC
AAGCGACGACTGTCTGCAGCAAGACGCGCAGGAACATCATGTGCAAACTGTCAAACGAGC
ACGACAACACTGTGGCGAAGAAACGCCAACGGGGACCCCGTGTGTAACGCTTGCGGTCTA
TACTACAAACTGCACAATATCAACCGACCTCTCACCATGAAGAAGGAGGGCATTCAGACC
AGGAACAGGAAGATGTCCAGCAAGTCGAAGAAGAGCAAAAAGTCCCACGACAGCATGGA
CGACTTCTCCAAGAGCCTGATGGAGAAGAGCAGCTCGTTCAGCCCCGCCGCTCTGTCCCGC
CACATGTCCTCCTTCCCCCCCTTCTCACACTCCGGCCACATGCTGTCCACCCCCACGCCCAT
GCACCCCTCCTCCTTCGCCCCTCACCCCCACTCCAGTATGGTCTCTGCCATGGGCTAG

STAT6 (CDQ66037, FR904526)

ATGGCACAGTGGACGCAGATGAAACAGCTGATCCCCTATCTGGCCACACAGATCCTCAAC
GAGCTCTACTCCCCCTCCTTCCCCATGGACGTCAGGTGCTACCTGGCCAACTGGATCGAGG
AGCAGAGATGGGAGGACTTTGAACCAGAGAAGGTAGACCAGGAGCCCCAGGCTCTGACC
CTGCTCGATCAGATCATATCCTGCCTACAGAATCTAGCCGGGCAGAACCCCAATGTGGTG
GAGAGGATGAGGCTGCAGCGTATCTCCAGGAACATGAATATGTTCCGTTCCCAACCTTTC
CAGCTGGTACTCACAGTCAGGGATGTCCTGAGGAAGGAGAGAGAGCTGCTCAGTCAGGC
GTCTACCCAGTTCCCGGCCCTAGCCCGCTTCTCTCAACCCCCAGCCCAGGATTCCATGGCT
GCAGCGCCCCCTACCAGAGACGTGGACCTGCTTGTGCTCAAGGTGCTTGAGATCCAGGAC

TGCAGACAGCAGATCCACCAGCTGCAGGAGGAGCTCAACTGGGAGAGGCAGAACTATGA
GTCCATGCAGGGCACTCTCCAGCAGACGCAGACCAATGGGATGGACACCAGCATGTCAG
AAGTCACCAAACTCCAGACTAACATCAAACAGCATGAGTACAATGGCCAAGTCATAGCC
AAGAAGCGTGTCCAGCTGCTCCAGGAGGCAGTGTCTAGTCTGGAGAAGTGTCAGTCTCGT
CTGATCAACAGTATCAAAGCCTGGAGGTGGGAACAGCATCAAGCTACTATTGGACACCCC
TTCGATGACAACCTCAGCCCTCTGCAGACGTGGTGTGAACAACTCCTGGGGGTGAACGGG
AAGTTGAGACAGGAAGTGATGCTGGCCGGGGAGCCAATCCCAGAGCTGCAAGAGAGACT
GGGACGTTTGCTGCAAACTCTAATCCAGAGCTCCCTGATGGTGGACAAGCAGCCTCCCCA
GGTGATTAAGACCCAGTCTAAGTTCTCCACCACGGTGCGCTACCTGCTGGGGGAGAAGGT
GGCACCCGGGAAACCAGTCCTCTTCAAGGCCCAGATCATCACTGAGGCCCAGGCTAGGAA
CCTCGGACAGCTGGGCAGCTTACCCAGTGAGAATGTTGGAGAGTTGATCAATAACACAGC
CATTTTGGAACACAACACTTCCGGCAAGAGTACATGTGCCACCTTCAGGAATATGTCTAT
AAAGAAGATCAAGCGAGCAGACAGAAAAGGGTCAGAGTCTGTTACAGAGGAGAAGTTTG
CTCTCTTCTTCACCACAGAGATCAGCATCACTGGCTGTGACATTCCCTACAGAATACAGAT
GATCTCTGTGCCGGTGGTGGTGATCGTTCACGGTAGTCAGGATAACAATGCCCTAGCAAC
CATCATCTGGGACTGTGCCTTTTCTGAACCAGAAAGGATTCCGTTTTTGGTTCCGGAACGC
GTTCCCTGGAAGCAGATGTGCAGGACCCTCAACAGTAAGTTCATGTCGGAGGTCGGGACG
CAGCGTTGCCTAGACAACTACAACCAACATTTCCTGGCCCAAAAGATCTTCGACAAGCCA
GACATCGCTGGAGACTACAGCAACATGCTGGTATCCTGGGCACAGTTCAACAAGGAGGTG
CTCCCTGGACGACCTTTCACCTTCTGGCAGTGGTTTGATGGTGTGATAGAGCTTACCAAGA
AGCACCTGAAGAGCTACTGGAGTGACGGGTTGATATTTGGTTTCATCGGTAAGCAGCACC
TCCACCTGATTCTGAAGGACCGGCCCAACGGGACCTTCTTACTGCGCTTCAGCGACTCTGA
GATAGGAGGCATCACCATTGCATACGTGGCTCCATCAGAGAACGGGGGCCAGAAGATCC
AGAATATTCAACCGTCCACCAAGAGAGACTTGGAGATCCGTTCCCTTGGTGACCGTATTC
AAGACATCGACTTCATCACACACGTTTATCCAGATCTGCCGAAGAATGACGTGTTCAGAA
AATACTACACAGGCAAGTCGCGCGCGCACACACACACCACCACACACACACACACACAC
ACACACACACACACACACACACACAAACAGACTTCACAAGGGACCAGGGGAATGGAGCC
AGTGCATCACCATTCTACTCTCTCTCTCCGAACAACCAGTTCCAAACCCCCTTCCAGCTGG
CCCCAGTACCCCTACAGGGGGGGATGTATCCTGACCCCCTCTCCAATCCCAATGGACTCTT
TCCTGGCTCGGACATGTGAGTGA

<u>IL-4/13A (AB574337)</u>

ATGAAGACGGCGATCCTTCTCCTCTCTGTTGCGATGGTGCTATTTGACTCTGGAACTCTGC
CTGCCAACGCTGCAGCGCACCGGCAGCATAGAAGTCACAATTCCAATTTAGAGGCAATTA
TTAGCATGGCAGGACAATACACACACACTCTGTCAGAGGAACTTCTGGAAACATTACTCA
TAGATGTAACACATTTGACTGCGACCACCACAAAGTGCAAGGAGTTCTTCTGCGAAGCTG
AGACAATCTTGGCCTCCGTGAAAAATGACGCGTTTGGTGAGGAGGGAAAGATTGTCCGAC
ATCTGAGGGTTTACAACAAACTTCAGAAGTGCACCGTTGTGAAGAGCCAAGACCAGGTGG
AGGGCAACCAGGTAGAGCTGAGGAGACTTCTCCTCGACCTAAAAGAATGTGGCCAAAAA
ATCAACTCCAAACCATGA

IL-4/13B-1

ATGAAGACTCTTGCTCTCCTCTTCTCCTTTGCATTTGTGCTGGTCTTCACTGCGCCCACTAA
GACACCTGATGAGATTCATCTACTGCAGAGGATCATGAAAGAGGCTAACAAGACGCTCA
AGGAAGGCCCGGAGGCCCTTTTGAACCATCTAGTGCCAGCGGAATTCAAGCAAGATCGCT
GCAAGTCACACGGGCCTAAAGATTTCTGCATAGCGGAGACGATTCTGTCTAACATAAACA
AAACGCAGTACGGGACTTCAGACAACAACATAGAGACAATCAGCAGAGTGCTGGAGCAG
TACAATAAGCTTCACCCTTCCAACTGCACTATGAAGACGAATGGAGACGAGGAACAGCTC
CGTGCTCTCCTGATGAACCTCTACAACTGTGCCCAAGCCATCTATTCCCGTCCCCACCCTG
TCACTCATCACAAAACGACCCCAGCACTTTGA

IL-4/13B-2

ATGAAGACTGTCGCTCTCCTCTTCTCCTTTGCATTTGTGCTGGTCTTCACTGCGCCCACTAA
GACATCTGATGAGACTCATCTATTGCATATGATCATCGACGAGGTTAACAAGATCCTCAA
AGGAGGTCTGGAGTTTCAGGCCCTTTTGGACAGTCTAGTGCCGGCGGGATTCGATCAAGA
TCGCTGCAGGGCAAACGGGCCTAAAGATTTCTGCATAGCTGAGACTATTCTGTCTGCCAT
AAGCCATACGAGGCACAGGATTCCATTTAACAACATATCTAAAATCAGTAGAGTGCTGAA
GCAGTACAATAAGTTTCATCCAACCAACTGCAATGTGAAGGAGAACGGTGATGAGGAAC
AGCTCCACGATCTCCTGACAAACCTTGTCAACTGTGCCAAGGTCATCTACTCTCGTCCCCC
TCCCTGCCGGTCTTCACAAACAACCCTAGAGCTTTGCTGA

Atlantic salmon

T$_H$1-Signature Genes

T-bet (NM_001199226)

ATGGGCGGCATAGGTGGCAATCTTTACCTCAGTATGTTGAATGGGACTGATACGCAAACT
TTCGGAAAAGACACCGATATCAACAATCACCTTCAACGCAGAAAGAATTCCGTGGATTTT
AAAATGGGAATTCAGGACACCAGATTATATCACCAAGACTCAATTCAAAACGGACAGGA
CGGATCGGCACTGCCGTACCACGCAGACCAAACTGTGGCTGGATACGGAGCGCAACCCG
GGAGGTTTTACTCACCAACTCCCCACAATAGCTGCCATTTCAGCAGTGTCCGGTCGCCGAC
TAGAAACGGAACGGGACAGACTTATTTACCAACGGGAAGCGAAGGTTTCTCGACAACTA
GCAAAGAAGTTTACCCTGCCTCTGCGGAGAGTTATCCCGCACCCGTTCAACATGGGTACT
ATCGCGCGCCACTCTACCCGTTACCTGGGCTACAGGTGTGCGGGAAGACGCAGGCTCTCC
TCAACAACTATCCTCTGTGGGCGAAGTTCCACAAATATCAAACCGAAATGATCATAACGA
AACAGGGGCGGAGGATGTTCCCCTTCCTGAGTTTCAACATCAGCGTTCTGGACCCGTCAG
CCCACTACAATGTGTATGTGGACGTGGTCCTATCCGACCAGCACCACTGGAGGTACCAGG
GAGGCAAGTGGGTCCAGTGTGGCAAAGCAGAGGGTAACATGCCAGGGAACAGGAGGTAT
ATGCACCCAGACTCCCCCAACACAGGGGCTCACTGGATGAGGCAGGAGGTGTCCTTTGGC
AAGCTCAAGCTCACCAACAACAAGGGCAGCGCCAACAACGTGGGGCAGATGATCGTTCT
GCAGTCGCTTCATAAGTACCAGCCACGGCTGCACATCATCGAAGTGAAGGAGGATGGTTC
TGAGGACCTCTTCCTCACCGCCAAGGCCCAGACCTTCGTCTTCCCAGAGACCCAATTCATA

GCCGTCACAGCCTATCAGAACGCAGACATCACCCAGCTGAAAATAGACCATAACCCCTTT
GCTAAAGGTTTCCGGGACAATTATGACGCGCTGTACGCACCGCCTGACTCGGACCGTGTC
ACCCCCTCTCCCACCGAGGGCCAGCAGCTTTTGGCAGGTACCTGCTATACCCAGGGGTAC
CTGATGGACCAGTACATGAACCCTCTGCCCCAGAACCGCTTCTACAGCCGGGAGCACATG
GCAGCCATGGGCCATCAGACCAAAGACCCCTCAGCCAGCCCCCACAGCCGCTGGTACCTC
TCCCCCCAGCAAAGTGTCACCTCCAACCGGCTGGACTTCAACTCCTATGAGGGTGACTTCT
CCAGCAACAGCTTCTACAAGCCCTTCCCCCTGCAGACATCGGCCCACCACACGCTGAGCT
ATTATGAGGACAGCCCGTTCGCCACGGCCAGCGGATCCGTCTCAGGAGCCTCCTCGGCAG
CCTGGAGCACCGGCCGGCCCTCCCCTCAGTACCTCAACCATCCCTGTCCCAGTAAAGTCG
GCCCCAGTCTAGGCTGGTTCAGGCCTGTGTCCTCTACCTCTCCAGGTCACCCCAGGCTCCA
CCCCCCGTCCCTCCTGGAGCCACTCCGCCCCCCACCATCGCTGCAGGACAAGCCCAAGGA
GGCGGGGGTGGGGGGAGAGGACCCCTGGCTGGAACCCCCCTCGGTCAAATCGGTGGACT
CTGCTGACTCCGGCCTGTTTGAGGGCGGGGCAGAGGGCAAGAAGCGGCGAGTGTCACCCT
ACGCTTCCAGCACGGAGAACTCGCCGCCCACCCGCAGCGTGGAGGTCTGTGAAAAGGACT
GCGGTAGTGATGCCGGCTACTACGGCTTTTATTCCCACTGA

STAT1 (BT045567)

ATGGCCCAGTGGTGCCAGCTGCAGCTACTGGAGTCTAAATACCTGGAGCAGGTCGACCAG
CTCTACGATGACTCCTTCCCCATGGACATCAGACAGTACCTCAGCAAGTGGATTGAGAGC
ATCGACTGGGAAAATGTGGCTGTTCAGGACTCCTTAGCAACAGTACGTTTCCATGACCTC
CTAGCCCAGCTGGATGATCAACACAGCCGCTTCGCCCTGGAAAACAACTTCCTGCTACAA
CACAACATCCGCAAGATCAAGAGAAACCTCCAGGACCACTTTCAGGAGGACCCTGTTCAC
ATGGCCATGATCATCTCCAGAAACCTGAAGGAGGAGCAGAAGATTCTGGCTGCTGCCAAA
AGCATAGAGACCGACAGAGAAAACACACAGACTAGCATGGTTCTGGAGAAACAAAAGCT
GGACAACAAAGTCAAAGACATGAAAAACAAGGTTCAGGAAGCGGATCAGAATGTGAAGT
CTCTGGAATACCTTCAAGACGAGCATGATTTTAAGGAAAATACTCTTAAGAACAGAGAAC
ATGAAATGAACGGGCTGACACAGAAACAGCTGGAACACGAGAAGTTGCAGATTGCAGAG
ATGTGTTTCAAGCTGAAGTTCAAGAGAGGGGAAGTGGTTGGCCAGTTGGCGGAGGTGCTC
AACATTGCCGAGGCGGTCCAGTCAGACCTGATCTCAGAGGAGCTGGCGGAGTGGAAGAA
GAGGCAGCAGATCTCCTGTATTGGAGGACCACCCAACGCATGTCTGGACCAACTGCAGAA
CTGGTTCACAGCGGTGGCGGAAAGCCTGCAGCAGGTGAGGCAGCAGCTGAAGGAGCTCC
AGGAACTAGAGCAGAAGTACACATACGACAACGACCCAATCAAACAGCAGAAAGGCTTC
CTGGAGGGGCGGGCCCTGTCACTGTTCCGGAACCTCCTAGAACATTCCTTGGTGGTGGAG
AGACAGCCCTGTATGCCCACACATCCACAGAGACCTCTGGTGCTGAAGACACAGGTGCAG
TTCACTGTCAAGCTCAGATTTCTTGTGAAGCTCCAGGAATTCAATTATCAACTCAAAGTGA
AAGCTTTGTTTGACAAGGATGTTACAGAAAAGAAAGGGTTTCGTAAGTTCAACATATTGG
GTACCAACACAAAAGTTATGAATATGGAGGAGTCAAATGGAAGCTTGGCTGCAGAGTTTC
GCCATTTGCAACTGAAAGAGCAGAAAGTAGCCGGCAACAGAACAAATGAGGGGCCTCTC
ATTGTGACAGAGGAGCTACACTGCATCTGCTTTGAATCTGAACTCAACCAGTCAGGTCTT
GAGCTCAAGCTAGAGACCATCTCACTGCCCATCGTGGTCATCTCCAACGTCAGCCAGTTG
CCCAGTGGCTGGGCCTCCATCCTGTGGTACAACATGCTGACCAGCGAGCCCAAGAACCTG

AAGTTCTTCCTGAGCCCTCCTGCAGCGTCCTGGGGTCAGCTGTCTGAGGTGCTGAGCTGGC
AGTTCTCCTCTGTCACCAAGAGGGGACTCAATGAAGAGCAGCTAGGCATGCTGGCTGACA
AACTACTGGGCCAGAAAGCACAGAGGAATCCTGAGGGCCTTGTCCCCTGGACCAAGTTCT
GTAAGAGTCTCAGTGAGAAGAGCTTTCCCTTCTGGCTGTGGATCGAAGCTATCCTGGACC
TCATCAAGAGACATCTGCTGTCCCTGTGGAACGATGGTTGCATCCTGGGCTTTGTGAGTAA
GGAGAGGGAGAAGGCCCTGCTGACTGGGAAGTGTCCAGGCACGTTCTTGCTGCGCTTCAG
TGAGAGTAGCAGAGACGGAGCCATCACATTCACTTGGGTGGAACACGACCAATACGATA
AGCCTGTATTCCATGCAGTGGAGCCCTACACCAAGAAGGAGCTGTCTGCCGTCTCTCTGC
CTGACATCATCCGCACCTACAAGGTGATGGCAGCTGAGAACATCCCAGAGAACCCACTCC
GCTTCCTCTACCCAGACATCCCCAAAGACAAGTCCTTCGGAAAGTACTACGCTAGGGCCT
CAGAGGCATCGGAGCCTATGGATGTGGAGAGTTCCAGCAGCACAGGATACATGAAGACA
GAACTCATATCAGTGTCAGAAGTTCACCCCTCTAGACTGCAAGACAACATGATGCCCATG
TCGCCAGACGTGTTCGGAGAGTTGAAGAGGAGCGTTGCGCCAGTGTTCCAGTGTTGGACT
GGTCCTAAGGAAATTGATGCTGTGGCGTCTGATCTCGGAGAGTTTTTAGCTGAAGAATTTC
AGATGTTTGAGAACACACCTGACTTGGACCGCAACTGA

STAT4 (NM_001173670)

ATGAGTCAATGGAAACAAATTCAACAGCTCCAGATCAAATACCAGGAGCAGGTGGGCTA
CCTTTATGATGACAACTTCCCTATGGACATACGGCAGGTGCTCTCCAACTATATTGAGAGC
CAGGACTGGGATACTGCCGCAAACCATGAATCCATGGCGACAGTTCTCCTCAGCAATCTC
CTGTCTCAGCTGGACAGGCAGTGTTCTCAGGAACAGAACTTCCTACAAAGACACAACTTG
AAGTTCATCTACCAGCAGTTACAGACTAAATGCAAAGCCAGCCCTCTGCTGATGGCTGGG
GTCATTTCTTCTTGCCTCAGAGAGGAGCGCCGTATCATCTCCACAGCCAGCATTCAGGAAC
AGGGCCCATTGGAGAAGTCAATGCAGAATTCGGTGGCCTTCGAGAGACAGAAGAACATG
GACAACAGGGTGGCGGTGATCAGGAGTAGTGTGCAGATGATGGACCAGGCAGTGAAATA
CCTAGAAGACATGCAAGACGACTTTGACTTCCGCTATAAAACTCTCCAGTCTCGAGATCC
AGTGGACAGGAACACAGCGGTAATGAAACAGGAAGTGACCCGACTGCAGGAGATGCTCA
ACAACCTGGACTTTAAGAGGAAGGAGAACCTATCTAAGATGAGTGACGTGATAAAAGAG
ATTGATGCTCTGATAGTCTCCCAGCTGAACCCAGAGCTGATGGAGTGGAAACGCAGGCAG
CAGATCAACTGTATAGGTGGCCCACTGCTCACTGGACTGGACCAGCTCCAGAACTGGTTT
ACTCTGACAGCCCAAAGTCTGTTCCAGATGAAGCGTCAGCTGGACAAACTGGGGGAGCTT
ATATTAAAGGTGACATATGAGAGCGACCCCATCCCACTGCAGAGACCGCAGATGGAGGA
GCAGGTCAAATACCTCATCTACCACCTCATCAAAAGCTCCTTTGTGGTGGAGAAGCAGCC
ATGTATGCCAACCCACCCCCAGAAACCTCTCATCATCAAGACCGGGGTGCAGTTCACCAC
CAAAGTCAGATTGCTGGTCAAACTTCCAGAGGTGGATTACCAACTAAAAGTGAAGACAAT
ATTTGACAAGGACCTCCCCCCTGGCAGAGTAAATCGTCAGTTCTTCATCCTGACTACCACC
ACCAAAGTAATGGACGTGGAGGAATCATCCAATGGCTGTCTGTCTGTAGAGTTTAGACAC
TTGCAGCTGAAAGAGAAGAAATACGTTAATGGAACCAAGGTGAACGAGGGTCCTGTCTCT
GTGACGGAGGAGCTCCACTCCCTCAGCTTTGAAGCCCAGTTCAACATACAGGGCATTAAC
ATCGACCTGGAGACCTGCTCCTTTCCTCTGGTGGTCATCTCCAATGTTAGCCAGCTGCCTG
GAGGCTGGGCATCTGTCATGTGGTACAACCTGCTGACTGACGAACCCAGGAACCTGGGCT

TCTTTGCCAGTCCTCAGCGGGCCAGTTGGGGCCAGCTCTCTGAGGTACTGAGTTGGCAGTT
CTCCACCTTCGCTGGGCGGGGCCTCAACAGGGAACAGCTGTGCATGCTAGGAGAGAAACT
CTTGGGTCAGCAAGTCTCCCAAAACGAATGTCAGGTATCCTGGTCCAAATTCTGCAAGGA
GAATATTCATGGGAAGAATTTCAGTTTCTGGATGTGGCTGGATTCCATCCTGGAGCTCATC
AAGAAGCACCTACTTCCCATCTGGAACGACAACTGCATCATGGGTTTTGTCAGTAAGGAG
ACTGAGAGGGCCCGTCTGAAGGACAAGGAGCCTGGGACTTTCCTGCTGCGCTTCAGCGAG
AGCCACCTGGGTGGGATCACCTTCACCTGGGTGGAGCAGTCCGATAGTGGAGAGGCCAA
GTTCATCTCGGTGGAGCCGTACACCAAATCACGCCTCAGCGCTCTCCCGTTTGCCGACATT
ATCCGAGACTACAAGGTCATCACAGACGGAGAGGTCCCTGAGAACCCACTAAAGTTCCTC
TACCCAGACATCCCCAAAGACGAGGCCTTCGGACGACTTTACAACAGCCAGCAGAACAA
AGTGTTTCCATACATACCATCTATGCTGATCCCCATCTCAACTTTGCAAACCACACCTCCC
CCCTGCTCCTCCCCAGAACCCCCCATGTCCCCTGGGATGTTTGACATGCTCACCCAGCACC
TCAGCCCCTTGGAGATTGAGACTGCCATGAGATCTCCGTACTCCGAGTAA

IFNγ (NM_001123558)

ATGGATGTGTTATCAAGGGCTGTGATGTGTTTCTGCTTGATGGGCTGGATGACTTTAGGAT
GGAGTAATGCCGCTCAGTACACATCAATTAACATGAAGAGCAACATAGACAAACTGAAGG
TCCACTATAAGATCTCCAAGGACCAGCTGTTCAACGGAAAACCTGTTTTCCCCAAGGACAC
GTTTGAGGACAGTGAGCGGAGGGTGTGGATGAGTGTGGTTCTGGACGTGTATCGGAGTATC
TTCAACCAGATGCTGAACCAGACGGGGGACCAGGAAGTGAGGGAGAGGCTGGACCAGGTC
AAGGGGAAGGTTCAGGAGACCCAGAAACACTACTTCCTGAAAAGGATACCTGAGCTGAGG
ACACACCTGCAGAACCTGTGGGCCATCGAGACCAGTAACACCACAGTCCAGGGGAAGGCT
CTGTCCGAGTTCATTACCATCTACGAGAAAGCCTCCAAACTGGCCCTTAAGATCCATCTAA
AGAAGGACAACCGCAGGAAGAGACGGCAAGCCCAGAGGCTCAAATCAAGCATCATGTAG

T_H17-Signature Genes

IL-17A/F1a (KJ921970)

ATGGGTTCTACTTCAAACGTACACTTTTTGATGGTGCTGTGTTTAATGGGGTTATTGGAGA
TGATGATGATGGGTGCAGAGGCAGCCCCCCACGTCCATCCAGCGATGACAGGGAGATGCT
CACAGAGAAACCTTCAGGGACACAATAAGCCTGTAGAGCCTTCAGCTCCAAACACAATA
ACTGTCCCCCTGCACCTGGACCCGAGCGACTTGATTCCTTCCCGTCTCGTCAGATCCATTG
GGAACCACTCGATCTCCCCATGGACTACCAACACCACCTATGATGAGTCCCGCTTTCCCCA
AACCATCTCGGAGGTCAGGTGCTCTCTGGAGGGCTGTCTGAACGTCGCGGGTAAGGAGGA
CAGAAGCCTGGAGTCCAAACCCATTTACCACCAGATCCTGGTCCTGAGGAAGGTGATGGG
GAGCGTGGACAGATACTACTACCGGCTGGAGTCAAAGATCATCGTGGTGGGCTGCACATG
TGTCAGGCCTAGCATTGAGCACCAACAGTAA

IL-17A/F1b (KJ921971)

ATGGTATCTACTTTAAGCTTCCAGTCTTTGACGGTGCTGTGTATGAAGGGGATGTTGGTGA
TGATGATGGGGGCAGAGGTGGCCCCCCGTGCCCATTCAGAGAAGACAGAGAGCCACACA

CGGACCACACTCCAGACACACAAGAAGCCTACAGAGTCTACAGGTCCCGACACAGTAATT
CTCTCCCTGCACCTAGACCCAAATTACTTGGTTCCTTTCCATTCCATCAGACCCATTTGGA
ACCATTCAATCTCCCCGTGGACTTACAACACTACCTATGACGATCGACGCTTTCCCCCCAT
CATCTCGGAGGTCAGGTGCTCTCTGAAGGGATGTCTGAACATCAAGGGTAAGGAGGACA
GGAACCTGAAGTCCAAACCCATCTTCTACCAGATCCTAGTCCTGAGGATGGTGATGGGAA
GTGGGAAGAAATGCCACTACCGGCTGGAGTCAAAGGTCATCTCAGTGGGCTGCACCTGTG
TCAGGCCTACCATCGAACAGTAA

IL-17A/F2a (KJ921972)

ATGGAGCTCAAAAGCAACGTGTCGAAGTACCTGGTTGTGTGCTGTGTGTCTATGCTGCTG
GGCCTGACCATGGCAAAAGGAAAGAAGGGGACAAAGGAGAGGTGTAACGAAACACTGA
TCATACCTTCAGACTACTACAAGATTCCCACAGAGGAATCAGAGGGAAATGGGAACATTC
ACACACGCTCTCTGTCACCCTGGACCTGGAAAACCACTACAGTGGAGAACCGTCTTCCTC
AGACCATGTGGGAGGCCGAGTGCAGCTCTATGTACTGTGTCTACCCCACCAACAGCAGCC
AGTACGAGCAGAACTCTGTACCTATCTACCAGCAGGTCGTGGTACTCTACACTTCAGCCA
CCAGGAAGTGCTACAGCGCCACCTTCCTGTCTGTGGCCGTGGGGTGCACCTGTGCCTGGG
CAAGGACCTCTTGA

IL-17A/F2b (KJ921973)

ATGTGGAAGACATCAGAGATGGAGCTCAAAAACAACACGTCGAAATACCTGGTTGTGTG
CTGTGTGTCTATGCTGCTGGGCCTGACAATGGGCAAAGGGAAGGTGGGGGAGAAGGAGA
GGTGTGAGGACACACTGACCATTCCTTCAGACTACTACAAGACTCCTTCAGAGGAATCAG
AGGGAAACGGGAACATCAACACACGCTCCCTGTCCGCCTGGACCTGGAAACCCATTACAG
TGGAGAACCGTATTCCTTCAACCATTTGGGAGGCCAAGTGTAGCTCTATGTACTGTGTCTA
CCCCACCAACAGCAGCCAGGTCGTGGGCTACCGGCAGAACTCTGTACCTATCTACCAGAA
GGTCCTGGTGCTCCATATCTCAGCCACTAGGAAGTGCTACAGCGTCTCCTTCCTGTCCGTG
GCCGTGGGGTGCACCTGTGCCTGGGCTAGAACCTCTTGA

IL-17A/F3 (KJ921974)

ATGCTTCTGGTCATGGTCTTCAGAGGTTTGCTGGTGCTGGGTCTGATCATGTCGCTGGACG
GATGTAAGAAAGACCAGAAGACCAAACTGAAGGCAGCCAAAGGCTCCGGACGAGTCAAG
GTCTTTGGACGGAGAGTAATGACTGTAGGGTTGGTTCTGGATCCTAACCTGAAGACCGCT
GTCTTCCCTGTCCTTCATATAGCCAACAGGTCTCTCTCTCCATGGACATACAGCGACACAT
ACGATGAGACCCGAGTCCCTCAACATATCTCCCAGGCTCAGTGCCAGAGGTCTGGCTGTC
TAACCCCAGATGGAGAAGAGGACATGGGGTTGGAGTCCAAACCCATACGCCATCAGACC
TTGGTGCTCCGCAGGGTTCAGGGAGAGATGGGCAGCAGCGGAGGGAGGAGGAAGAAGG
GGAAGAAGAAGGGGTATTTCTATAAACTGGAGAGTGAGAGTGTAAACGTGGGCTGTACC
TGTGTTTTGGCCTTTCGAGGGAGGTTTTTCCTAGCCACCGTGCTTCTACACCTGCATTGCTT
GCTGTTTTGGGGTTTTAGGCTGGGTTTCTGTACAGCACTTTGA

IL-21 (EU816603, ACJ02096)

ATGAAGCTGCTTGTTTGTTGTCTCCTAGCCATCACCTGTTGTGTTTTGGCGAATGCAGACAA
CGTCGAGAGAACAATGAAGCTGACTGAAGTACTAAAGGAGTTAAGACAGCTGAACAAAAG
TGTGGAGCACAACGGCGTGATGTTGAACACTCCTACCATGGATATAGAGGAGTGCTGTTTC
CTATCTACCTTGGAGTGCTTCCAGAAGATGGTACCTCGTCTCAAGACCAAACAAAAGAAAT
TGCAGTGTAAAGTTAGCAGAAACCTCAACCCACTGACTTTTACAGGTGTAGACTCCTGCAG
TCGGGAGGAGAGAGAGAAAAAAGTCTGCCAAGGATGTGATTCCTACCCAATGAAGGACAG
CAGGGAATTTGTACAACAGTTGGAATCTCTTCTTCAAAAGGTAGCAGGTTATTATGAATAA

IL-22 (AGKD04046435, AGKD04023486)

ATGAAGTTCAGTTCGGTCCAACTCGTAGCCGCTGTGGTAGTGGTGATGAGCGTCTGTCTGC
TGCGCGAATCCGTGGCGCACTCCATCCATCGCCCGCTCAGCGCCCCCCTGCACAGCGCGG
ATACCGACACCATGGTACAGCTGGTGGCTCAACAGGCGCAAAGTTCTGACACTGATACTG
ACACTAAACTGATGCCAGACATCGATACTAAAAAGAACCACAGAGACATCTGCTGCCTGC
ATGCTAACATCCTGGACTTCTACCTGAGTAACATCCTGACCACTAAGGAGAAGCAGGACA
AGCATCATCCTAAACTCCCTGCCCTGAAGGAAGACCTGGCCCGAGTCAGCAGAGACCTGA
AGGAACACGGCTGTGCTATTAAACACTACAATGACCACCACCACAGCATTGCTTTCCGCA
AGAAGTTGGCCGAGATGGAGGAGGGGAAAGGGATAAAGAAAGCCATTGGAGAGATTGA
CATCCTCTTCACCTTCCTGAAAGACTTCTGTGTGCACGCCTAG

T_{reg}-Signature Genes

Foxp3 (NM_001198847)

ATGCCACAGACTGAGAGTGAGAGGTTGAAGAGTGGGCGGCTGAACAGTGGTAGACAGCA
GCAGCAGCGAGATCGGCGGGGTGAACACGGAGAGGAGCCAGACACACGTACCAAGCCA
AATGATTCTTTCTCACCTCGGCTCTATGCTCGTACCTCTGTTACTCAGATGGGTTTCCCATT
GATGATTAGACCTGGACGTCCACTTATGGCCTCATCTCAGCTCCAAAGCATACTGTTACAG
CAGTGTTCCAGTGAGGAGGAGGGGAAACCGTTCCTGCAGCGGGTGTCTCGGTGCACCCAG
CTGAGCCAGCACAGACCCTCTGTCCTCCGCCAGGGTGTCCTAGCTGCCCACGTACGACCA
CAGGCAGCGGCCGGTTCAGCTCAACCCATATCACTGTGCAAGGTGGAGGTGGACACAGG
GAGCCGTGGACAGTCCTCACCCCCACACTCTGAGCACTCTCCTGGGCCCACTCGTCACCC
CTCGCCCCTGAGGAGAGCCAGTCCTAAGCAGAGCTCCATCATCACCAGGCATCATGAGCC
AGGTCACCCAACCCTACGGAGCTCCAGTGCTCTGTTTCTCAACGGACTCTGCTGCTGGCCA
GGCTGCGATGCGGTATTTGAAGAATTTCCCCGTTTTTTGAAACACCTCCACTCTGACCATG
GCCATGGAGACAGAAGCATTGCACAGTGGAGGGTACAACAAGACATGGTGCAATACATG
GAGACCCAGCTGACTGTGGAAAAACAGAGACTCTTTGCAATGCAACTCCATCTGCACCTG
TCTGAACACAAGTCAACTGGCATGAAGGCAGGTTCAGATTGGCCCTACAGCCACAGCCTG
GCACTAGTCCTGCCCCAGAACCCTTCCCCGGCCCGGGCAGCAGATGGAGTGCCACGCTGC
GCCACTAAAGAGCCAGAGGAGCTGGCACAGCAGGAGTATTGGCCCGCTGCCGCTCCCCA
CCACCTACTCCCAGATCTTGTCCCGAGTGTTGAGTGTTACAAATACAACAACATCCGGCCT

CCGTACACCTACGCCTGCCTGATAAGATGGTCTATAATGGAGACTCCAGACAAGCAGCGC
TCTCTGAATGACATCTACAACTGGTTCACCACCATGTTCTTCTACTTCCGACACAACACGG
CCACATGGAAGAACGCTGTCCGTCATAATCTTAGCTTGCACAAGTGTTTTGTGCGAGTGG
AGGGAGGCAAGGGGGCTGTCTGGACAGTGGATGAAATGGAATATCAAAGGAGAAAGGG
ACAAAAATATCACAGGGATCATCATGTGAAATGGTTGGCACCCTTCTCCTTATTCCGTCCA
GAGGAACCTTGA

IL-10 (AGKD04000716)

ATGTCTCCCTCCTCTCTCCTCCTATCCGTGCTGCTGGTTGCAGCGCTGCAGTGTGAGCATGCC
CAGTGCAGACGTGCACCGTGCTCCGACCGTTGCTGCTCCTTTATAGAGGGCTTCCCCGTCAG
ACTGAAGGAACTCCGCACATCCTTCTCCACCATCAGAGATTACTATGAGGCTAATGACGAGC
TGGAGACTTCCCTGTTGGACGAAGGCATTCTACACCACTTGAAGAGCCCGGTGGGGTGTCAC
GCTATGGACAGCATCCTGAAGTTCTACCTCGACACGGTGTTGCCCACCGCCATGAACAACAG
AACGCAGAACAACCACTTTAAATCTCCCATCGACTCCATCGGAAACATCTTCCACGAGCTGA
AGAAAGAGATCGTCCTATGCAGGAACTACTTCTCCTGTAAGAAACCGTTTGACATCAACGA
GTTCATCTCCTCGTATAAGAAGATGCAGGGTAAGGGCCTTTATAAGGCAATGGGAGAGCTG
GACCTGCTCTTCAACTACATTGAGGAGTACCTGGTCTCTCAGAGACCCAAACACTGA

TGFβ1 (BT059581)

ATGAGGGCAGAATGTTTGACGCTGGTTGTCTTATTGGCGCTGGAATATATGTGTCGGAGTG
AAGCCATGTCCACCTGTAAGTCACTGGACTTGGAGCTGATGAAGAGAAAACGCATTGAGG
CCATCCGTGGACAGATACTGAGCAAGCTGCGGCTGCCCAAGGAGCCAGAGATTGACCAGG
AGGGAGACTTCGAGAAAGTCCCAACCTCCCTGATATCCATCTACAACAGCACAGTGGAGCT
GAGTGAGGAGCAGGTGCACACATGCATACCACCCACTCAGGATACAGAAGAGGAAGCATA
CTTTGCAAAGGAGGTCCACAAGTTCAACATGAAACAAAGTGAGAACAACATTAAGCACCA
AATGCTCTTCAACATGTCTGAGATGCGGTCTGTATTAGGGACAGATCGACTGCTCTCTCAA
GCTGAGCTCCGTCTCCTCATCAAAAATCGTGGCATGCAGGACAGCAGCGAGCAGAGGCTG
GAACTCTACAGAGGCGTTGGAGATAAGGCACGTTACATGAAGTCCCATTTTATCTCAAAGG
AATGGGCCAATCGCTGGATCTCCTTTGATGTTACACAGACTCTGAGGGAGTGGCTGCAAGG
GGCTGGAAAGGAGCAAGGATTCCAATTGAAGTTGCCTTGTGATTGTGGGAAAACAATGGA
AGAATTCCGCTTTGAAATAGCAGGGATGAACAAGCCCAGGGGAGATAAAGAAACTCTCTT
CATGAACATGCCAAAGCCTCACATTTTACTGATGTCACTTCCTGTGGAACGCCACAGCCAG
CTTAGCTCTCGGAAGAAACGACAAACCACTACTGAAGAGATCTGCTCAGACAAATCGGAG
AGTTGCTGTGTGCGAAAGCTTTACATTGACTTCCGTAAGGACCTGGGCTGGAAATGGATCC
ATGAACCCACTGGCTACTTTGCTAACTACTGCATGGGCCCCTGCACCTATATATGGAACAC
AGAGAACAAGTACTCCCAGGTACTGGCTCTGTATAAGCACCACAATCCTGGAGCCTCTGCC
CAACCTTGCTGTGTTCCACAGGTCTTGGAGCCCCTACCCATTCTCTACTATGTGGGTAGACA
ACACAAGGTGGAGCAGTTGTCCAATATGATTGTTAAGTCCTGCAAGTGTAGCTAG

TH2-Signature Genes

GATA3 (NM_001171800)

ATGGAAGTATCCGCCGACCAACCCCGATGGATGAGCCATCACCACCCATCGGTGCTAAAC
GAACAGCATCCCGGCTCGCACCACCCGGGCCTCGGCCACTCGTACATGGACCCCTCGCAGT
ATCCCCTCGCCGACGATGTGGATGTACTGTTTAATATCGACTCACAGGGCAACCACGTATC
TCCATACTATGGGAACTCAGTCCGAGCCGTCCAGAGGTATCCACCTCCTCCACATAGTAGT
CAGGTGTGTCGCCCCTCTCTACTGCACGGCTCTCTGCCGTGGCTGGACGGAAGCAAAGCGA
TGCAGCACCACAGCACTTCCCCGTGGAACCTGAGTCCTTTCCAGAAGAGCTCACTGCACCA
CGGTTCCCCGGCAGGTCTCTCTGTCTACCCTTCGGCGTCCTCCTCCTCCCTGTCTGGGGGCC
ACTCAAGCCCGCACCTCTTCACCTTTCCCCCCACTCCTCCGAAAGACGTGTCCCCAGACCCA
GGCATCTCCACTCCGGGCTCAAGCAGCAACAGTCGCCAACAGGAGGAGAAAGAGTGCATA
AAGTACCAGGTGTCCCTGGCCGAGAGCATGAAACTGGAGTCTCACAGCCGGAGCATGGCA
TCAATCGGAGCGCAATCCTCCGCCCACCATCATCACCCCATCGCCACATACCCATCCTATG
TCCCCGAATACGGCCCAGGCCTCTTCCCACCAAGTAGCCTGATAGGTGGGTCATCTTCTAG
TTATGGTTCCAAAACAAGACCAAAAACAAGGTCATGTTCAGAAGGTAGAGAGTGCGTTAA
CTGTGGAGCTACGTCGACCCCGCTCTGGAGGCGGGATGGCACGGGTCACTATTTGTGTAAC
GCCTGCGGACTCTATCACAAGATGAACGGGCAGAATCGGCCCCTCATCAAACCCAAGCGA
CGACTGTCTGCAGCAAGACGGGCAGGAACATCATGTGCAAACTGTCAAACGAGCACGACA
ACACTGTGGCGAAGAAACGCCAACGGGGACCCCGTGTGTAACGCTTGCGGTCTATACTAC
AAACTGCACAATATCAACCGACCTCTCACCATGAAGAAGGAGGGCATTCAGACCAGGAAC
AGGAAGATGTCCAGCAAGTCGAAGAAGAGCAAAAAGTCCCACGACAGCATGGACGACTTC
TCCAAGAGCCTGATGGAGAAGAGCAGCTCGTTCAGCCCCGCCGCTCTGTCCCGCCACATGT
CCTCCTTCCCCCCCTTCTCACACTCCGGCCACATGCTGTCCACCCCCACGCCCATGCACCCC
TCCTCCTTCGCCCCTCACCCCCACTCCAGTATGGTCTCTGCCATGGGCTAG

STAT6 (AGKD04000009, GBRB01023434)

ATGGCACAGTGGACGCAGATGAAACAGCTGATCCCCTATCTGGCCACACAGACCCTCAAC
GAGCTCTACTCCCCCTCCTTTCCCATGGACGTCAGGTGCTACCTGGCCAACTGGATCGAGG
AGCAGAGATGGGAGGACTTTGAACCAGAGAAGGTAGACCAGGAGCCCCAGGCTCTGACC
CTGCTCGATCAGATCATATCCTGCCTACAGAATCTAGCCGGGCAGAACCCCAACGTGGTG
GAGAGGATGAGGCTGCAGCATATCTCCAGGAACATGAATATGTTCCGCTCCCAACCTTTC
CAGCTGGTACTCACAGTCAGGGATGTCCTGAGGAAGGAGAGAGAGCTGCTCAGTCAAGC
GTCTACCCAGATCCCGGCCCTAGCCCTCTTCTCTCAACCCCCAGCCCAGGATTCCATGGCT
GCAGCCCCCCCCACCAGAGACGTGGACCTGCTTGTGCTCAAGGTGCTTGAGATCCAGGAC
TGCAGACAGCAGATCCACCAGCTGCAGGAGGAGCTCAACTGGGAGAGGCAGAACTATGA
GTCCATGCAGGGCACTCTCCAGCAGACCCAGACCAATGGGATGGACTCCAGCATTTCAGA
AGTCACCAAACTCCAGACTAACATCAAACAGCATGAGTACAATGGCCAAGTCATAGCCA
AGAAGCGTGTCCAGCTGCTCCAGGAGGCAGTGTCTAGTCTGGAGAAGTGTCAGTCTCGTC
TGATCAGCAGGATCAAAGCCTGGAGGTGGGAACAGCATCAAGCTACTATTGGACACCCCT
TCGATGACAACCTCAGCCCTCTGCAGACGTGGTGTGAACAACTCCTGGGGGTGAACGGGA

AGTTGAGACAGGAAGTGATGCTGGCCGGGGAACCAATCCCAGAGCTGCAAGAGAGACTG
GGACGTCTGCTGCAAACTCTAATCCAGAGCTCCCTGATGGTGGACAAGCAGCCTCCCCAG
GTGATTAAGACCCAGTCTAAGTTCTCCACCACGGTGCGCTACCTGCTGGGGGAGAAGGTG
GCACCCGGGAAACCAGTCCTCTTCAAGGCCCAGATCATCACTGAGGCCCAGGCTAGGAAC
CTCGGACAGCTGGGCAGCGTACCCAGTGAGAATGTTGGAGAGTTGATCAATAACACAGCC
ATTTTGGAACACAACACTTCCAGCAAGAGTACATGTGCCACCTTCAGGAACATGTCTATA
AAGAAGATCAAGCGAGCAGACAGAAAGGGGTCAGAGTCTGTTACAGAGGAGAAGTTTGC
GCTCTTCTTCACCACAGAGATCAGCATCACTGGCTGTGACATTCCCTACAGAATACAGAT
GATCTCTGTGCCGGTGGTGGTGATCGTTCACGGTAGTCAGGATAACAATGCCCTAGCAAC
CATCATCTGGGACTGTGCCTTTTCTGAACCGGAAAGGATTCCGTTTTTGGTTCCGGAACGC
GTTCCCTGGAAGCAGATGTGCAGGACCCTGAACAGTAAGTTCATGTCGGAGGTCGGAACG
CAGCGTTGCCTAGACAACTACAACCAACACTTCCTGGCCCAAAAGATCTTCGACAAGCCA
GACATCGCTGGAGACTACAGCAACATGCTGGTATCCTGGGCACAGTTCAACAAGGAGGTG
CTCCCTGGACGACCTTTCACCTTTTGGCAGTGGTTTGATGGTGTGATAGAGCTTACCAAGA
AGCACCTGAAGAGCTACTGGAGTGACGGGTTGATATTTGGTTTCATCGGTAAGCAGCACC
TCCACCTGATTCTGAAGGACCGGCCCAACGGGACCTTCTTACTGCGCTTCAGCGACTCTGA
GATAGGAGGCATCACCATCGCATACGTGGCTCCATCAGAGAACGGGGGCCAGAAGATCC
AGAATATTCAACCGTCCACCAAGAGAGACTTGGAGATCCGTTCCCTTGGTGACCGTATTC
AAGACATCGACTTCATCACACACGTGTATCCAGATCTGCCGAAGAATGACGTGTTCAGAA
AATACTACACAGGCAAGTCCACACACACACACACGAGTCTGAATCACTCACTCACATATG
CACACACACACACACTTCACAAGGTCTTCATCATTCTCTCTTAGGGACCAGGGGAATGGA
GCCAGTGCATCACCATTCTACTCTCTCTCTCCGAACAACCAGTTCCAAACCCCCTTCCAGC
TGGCCCCAGTACCCCTACAGGGGGGAATGTACTCTGACCCCCTCTCCAATCCCAATGGAC
TCTTCCCTGGCCCGGACATGTGAGTGA

IL-4/13A (AB574339)

ATGAAGTCGGTGATCCTTCTCTCTGTTGCGATGGTGCTATTTAACTCTGGAACTCTGCCTGCC
AACGCTGCAGCGTACCGGCAGCATAAAAATCACCATTCCAATTTAGAGGCAATTATTAGCA
TGGCGGAACAATACACACACAATCTGACAGAGGATCTTCTGAGAACATTACTCATAGATGT
TACACATTTGACTGGGACCACCACAAAATGCAAGGAGTTCTTCTGCGAAGCTGAGAAAATC
TTGGCCTCCGTGAAAAATGCCACGTTTGGAAATATTGTCCGACATCTGAGGGTTTACAACAA
ACATCAATACTGCATCGTTGTGAAGAGCCAAGACAACCAGGCGGAGGGCAACCAGGTGGA
GCTGAGGAGACTTCTCCTCGACTTAAAAGAATGTGGCCAAAAAATCAACTCCAAACCATGA

IL-4/13B1 (GBRB01017951)

ATGAAGACTCTTGCTCTCCTCTTCTCCTTTGCATTTGTGCTGGTCTTCACTGCGCCCACTAA
GACATCTGATGAGCATCATCTACTGAGGAGGATCATGATCAAGGCTAACAAGACCCGCGA
TGAAGGCCCGGAGGCCCTTTTGGACAGTCTAGTGCCAGCGCAATTCAATCAAGTTCGCTG
CAAGGAACACGGGCCTAAAGATTTCTGCATAGCGGAGAAGATTCTGTCTAACATAAACGA
AGAGCAGTACGGGACTTCAGACAACAACATTGGGACAATCAGCAGAGAGCTGAAGCAGT
ACAATATGCTTCACCCTTCCAACTGCACAGTGAAGAATAACGTTGACGAGGAACAGCTCC

GCGCTCTCCTGATGAACCTCTACGACTGTGCCCAAATCATCTATGCCCGTCCCCACCCTGT
CACTCATCACAAAACGACCCCAGCACTTTGA

IL-4/13B2 (AGKD04001089)

ATGAAGACTGTCACACTCCTCTTCTCCTTTGCATTTGTGCTGGTCTTCACTGCGCCCACTGA
TGAGACTCATCTATTGCTTACGATCATCGACGAGGCTAACAAGATCCTCAATGGAGGTTT
GGAGTTTCAGGCCCTTGTGGACAGTCTAGTGCCAGCGGGATTCGATCAAGATCGCTGCAG
GGAAAACGGGCCTGAAGATTTCTGCATAGCTGAGACTATTCTGTCTGCCATAAACCATGC
GCAGTACAGGAGTCCATTAAACAACATATCTAAAATCAGTAGAGTGCTGAAGCTGTACAA
TAAGTTTCATCCAACCAACTGCACTGTGAAGGAGAACGGTGATGAGGAACAGCTCCACGA
TCTCCTGACAAACCTCTTCAACTGTGCCCAGGCCATTTACTCTTGTCCCTCACCCTGCCGCT
CATCACAAACAACCCTATAG

References

1. Dijkstra, J.M. T_H2 and T_{reg} candidate genes in elephant shark. *Nature* **2014**, *511*, E7–E9.

© 2015 by the authors; licensee MDPI, Basel, Switzerland. This article is an open access article distributed under the terms and conditions of the Creative Commons Attribution license (http://creativecommons.org/licenses/by/4.0/).
